# Interpretable deep generative ensemble learning for single-cell omics with Hydra

**DOI:** 10.1038/s44320-026-00208-7

**Published:** 2026-04-11

**Authors:** Manoj M Wagle, Chunlei Liu, Zunpeng Liu, Yongheng Wang, Manolis Kellis, Ellis Patrick, Pengyi Yang

**Affiliations:** 1 School of Mathematics and Statistics, Faculty of Science, The University of Sydney, Camperdown, NSW Australia; 2 Computational Systems Biology Unit, Children’s Medical Research Institute, Faculty of Medicine and Health, The University of Sydney, Westmead, NSW Australia; 3 Sydney Precision Data Science Center, The University of Sydney, Camperdown, NSW Australia; 4 Computer Science and Artificial Intelligence Laboratory, Massachusetts Institute of Technology, Cambridge, MA USA; 5 Computational Biology Group, The Broad Institute of MIT and Harvard, Cambridge, MA USA; 6 Charles Perkins Center, The University of Sydney, Camperdown, NSW Australia; 7 Center for Cancer Research, Westmead Institute for Medical Research, Westmead, NSW Australia

**Keywords:** Chromatin, Transcription & Genomics, Computational Biology

## Abstract

Single-cell omics enable the dissection of cellular heterogeneity, yet the high dimensionality, inherent noise, and sparsity present significant challenges. These challenges are amplified for rare cell populations, which are often difficult to annotate reliably but can be central to development and disease. As single-cell assays increasingly capture multiple molecular layers, the integrative analysis of such multimodal data further increases complexity. Here, we propose Hydra, a deep generative framework based on an ensemble of variational autoencoders for effective learning of unimodal and multimodal single-cell omics data. Hydra implements interpretable modules for capturing cell-type-specific molecular signatures. The ensemble of such interpretable modules enables reproducible feature selection and robust cell-type annotation, with particular effectiveness for rare populations. We benchmarked Hydra on a repertoire of 21 datasets, including unimodal and multimodal single-cell omics data. Our results demonstrate that Hydra offers comparable to superior performance to several state-of-the-art methods. Finally, we highlight the utility of Hydra in robustly annotating brain cellular subtypes and preserving disease-relevant signatures using our previously published dataset that profiles Alzheimer’s disease.

## Synopsis

Hydra is an interpretable deep generative ensemble framework that jointly performs feature selection and cell type prediction in uni- and multimodal single-cell omics data across diverse biological systems.

- An ensemble of variational autoencoders with data augmentation is introduced to address class imbalance in single-cell datasets, enabling reliable annotation of rare cell populations.
- Post-hoc interpretability using Integrated Gradients identifies cell type-specific features that capture biologically meaningful molecular programs.
- Hydra was benchmarked against 13 state-of-the-art methods across 21 datasets spanning diverse single-cell and multimodal technologies, consistently achieving high performance.
- Application to medial frontal cortex datasets from Alzheimer’s disease demonstrates robust identification of 27 distinct cell populations across disease conditions.

## Abstract (web summary)

Hydra is an interpretable deep generative ensemble framework that jointly performs feature selection and cell type prediction in uni- and multimodal single-cell omics data across diverse biological systems.

## Graphical abstract

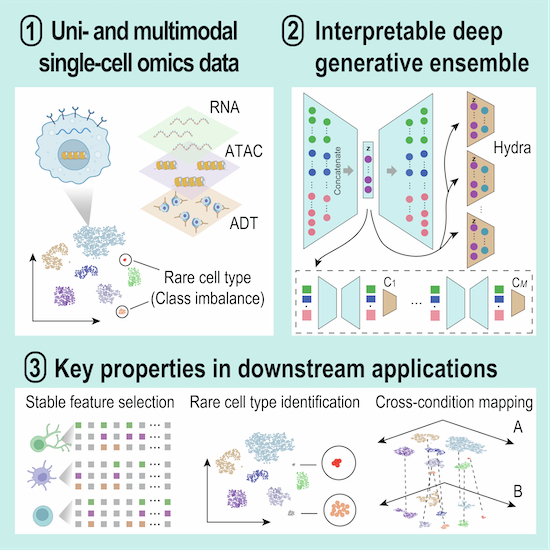

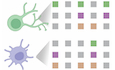

## Introduction

The establishment of single-cell transcriptomics has achieved remarkable success in revealing cell type-specific molecular variations by enabling the profiling of each individual cell (Aldridge and Teichmann, 2020; Shalek et al, 2013; Tang et al, 2009). More recently, there has been a transition toward single-cell multiomics, which allows for simultaneous probing of other modalities such as chromatin accessibility and surface proteins from the same cell in a single experiment (Baysoy et al, 2023; Buenrostro et al, 2015; Ma et al, 2020; Stoeckius et al, 2017). Such “paired data" enables a more comprehensive understanding of cellular function by capturing different layers of biological information, such as epigenetic and signaling regulation. Despite these advances, analyzing single-cell omics data presents its own set of challenges. Single-cell data is often sparse and noisy, with each cell characterized by thousands of features. This high dimensionality complicates the identification of key molecular signatures that underpin the biological variations and hinders downstream analyses such as cell type identification. Consequently, feature selection has become a useful step to identify cell-type-specific signatures and improve the signal-to-noise ratio in downstream analyses (Huang et al, 2023; Yang et al, 2021).

Accurate identification of cell types therefore requires the selected features to be reproducible and generalizable across studies and technologies. This is particularly important for supervised cell type prediction frameworks that utilize the selected features from reference datasets to identify cell types in new single-cell studies. A number of such supervised tools have been developed, including statistical and classical machine learning-based approaches, and more recently, deep learning models have gained increasing popularity in this domain due to their ability to handle the complexities of single-cell data (Clarke et al, 2021; Ma and Xu, 2022; Pasquini et al, 2021). Nevertheless, three key challenges remain. First, most existing tools are developed specifically for single-cell transcriptomic data and do not account for other single-cell omics modalities, limiting their applicability in multi-omic contexts. Second, existing methods often fail to detect smaller cell populations and are biased toward major cell types, which hinders their ability to recover rare cell populations that may nevertheless play critical roles in development and disease. Finally, deep learning-based approaches often lack interpretability, which makes it difficult to understand their predictions (Li et al, 2022; Wagle et al, 2024).

To bridge this gap, we introduce Hydra, a deep generative framework designed to effectively handle unimodal and multimodal single-cell omics data that profile gene expression, chromatin accessibility, and surface protein abundance. A key strength of Hydra is its ability to reliably recover and annotate rare cell populations, where class imbalance and weak marker signals pose challenges for existing methods. In particular, the feature ranking module of Hydra is based on training an ensemble of variational autoencoders (VAEs), each paired with a cell type classification head for post hoc feature attribution using Integrated Gradients (Sundararajan et al, 2017; Wagle et al, 2024). This ensemble deep learning approach (Cao et al, 2020) provides a ranked list of cell type-specific markers that highlight the key molecular features distinguishing each population, and is especially effective for rare cell types where existing methods often fail to recover robust markers.

By learning the probability distributions of the data via VAEs, Hydra can simulate new samples for smaller cell type populations. This enables data augmentation for learning from balanced datasets and thus addresses the issue of class imbalance. The molecular features identified by the feature ranking module of Hydra are utilized by its cell type mapping module, which again employs ensemble learning using simple neural networks to automatically predict cell types in query datasets. The architecture of Hydra is adaptable for both unimodal and paired multimodal single-cell omics data. Using 21 datasets generated from various tissue types and diverse single-cell omics technologies, we demonstrate that Hydra improves feature selection reproducibility and outperforms several popular tools, especially on rare cell type annotation. Together, these results highlight the utility and flexibility of Hydra for feature selection and cell type annotation of diverse unimodal and multimodal single-cell omics data.

## Results

### Overview of the Hydra framework

**Fig. 1:**
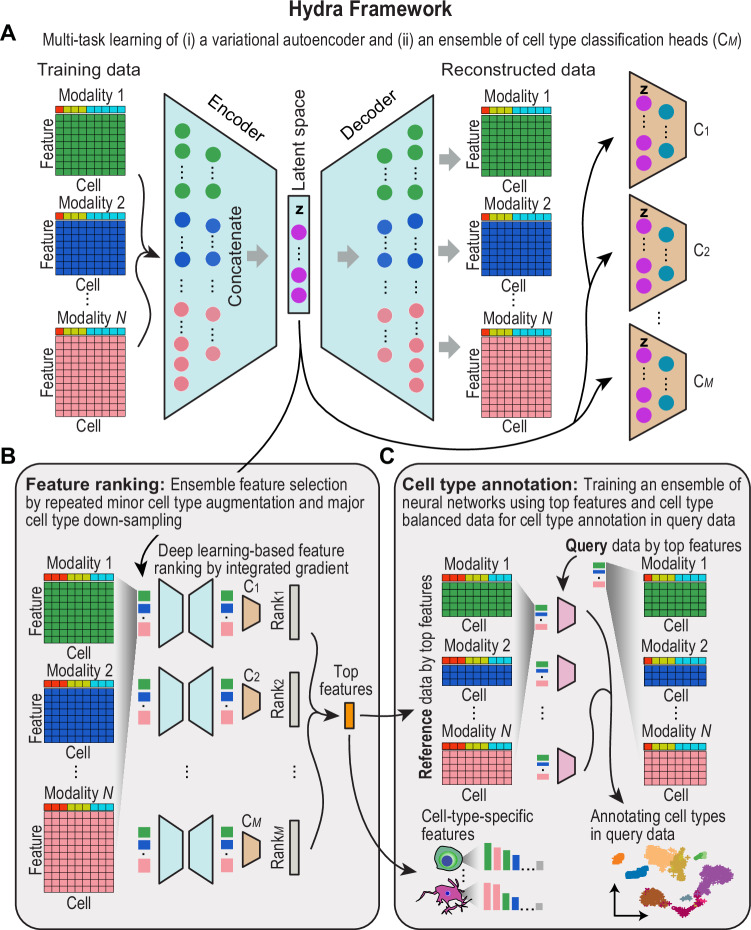
Overview of the Hydra framework for ensemble feature ranking and automated cell type annotation. (**A**) Graphical illustration of key architectural components in the Hydra framework, including the variational autoencoder (VAE) and the ensemble of cell type classification heads. (**B**) Schematics of the feature ranking module of the Hydra framework. Data augmentation and down-sampling are used to balance minor and major cell types. Multiple cell-type balanced datasets, each paired with a cell type classification head, are used for feature ranking, and the consensus is taken to derive top features. (**C**) Schematics of the cell type annotation module of the Hydra framework. An ensemble of simple neural networks is trained on dimension-reduced and cell-type-balanced datasets generated from the feature ranking module. These trained neural networks are then employed to predict cell types in any provided query datasets.

We developed Hydra, an interpretable ensemble deep learning framework for jointly identifying cell type-specific markers and mapping cell types in unimodal data, such as single-cell transcriptomes, as well as multimodal data such as those that jointly measure transcriptomes, chromatin accessibility, and protein expression in individual cells. Specifically, Hydra extends on the variational autoencoder (VAE) architecture utilizing a multi-task training procedure (Liu et al, 2023) (Fig. 1A), and contains two modules (i) an ensemble feature ranking module (Fig. 1B) and (ii) an ensemble cell type annotation module (Fig. 1C). First, the VAE architecture is jointly trained with an ensemble of cell type classification heads on the original dataset (Fig. 1A). The model is optimized by a loss function that takes into account the reconstruction loss from the decoder and the loss from the cell type classification heads. Hydra also minimizes overfitting on the training data by adopting an early stopping approach.

In the feature ranking module, we address cell type imbalance by using the trained VAE to generate synthetic cells for minor cell types and randomly downsample major cell type populations to create balanced datasets (“Methods”). We initialize models by inheriting weights from the original trained model and specific classification heads and fine-tune each model using each corresponding balanced dataset (Fig. 1B). Each fine-tuned model is used for feature ranking by employing the post hoc feature attribution approach of Integrated Gradients (Sundararajan et al, 2017). This approach accounts for post hoc interpretability and obtains cell type-specific ranking of features, considering the biological direction of molecular changes. Finally, feature rankings from individual models are combined to derive consensus top features. In the cell type annotation module, an ensemble of simple neural network classifiers is trained, each on a balanced dataset filtered by top features selected in the feature ranking module (Fig. 1C). After training, the predictions of cells to cell types for a given query dataset are averaged across individual classifiers to obtain the final cell type.

### Ensemble deep learning improves the feature stability of Hydra

**Fig. 2:**
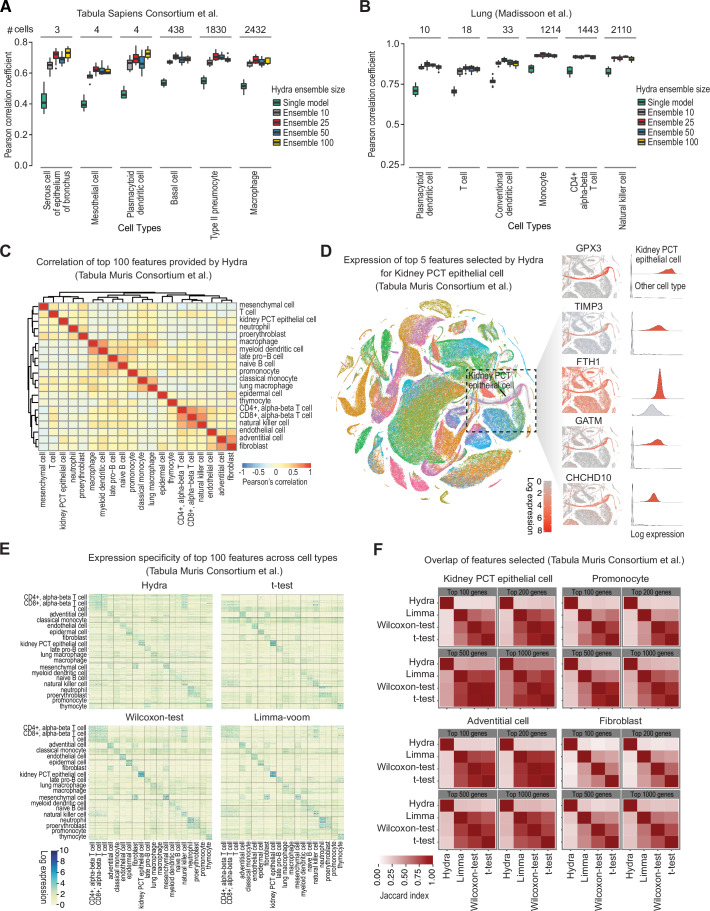
Evaluation of Hydra feature stability, cell type specificity, and correlation across different methods. (**A**, **B**) Feature selection stability of Hydra quantified using Pearson’s correlation coefficient using different ensemble sizes (*n* = 1, 10, 25, 50, 100) on (**A**) Tabula Sapiens and (**B**) Lung datasets. The three smallest and three largest cell types from each dataset are shown, arranged in the order of increasing sample count from left to right. Box plots display the median (center line), interquartile range (IQR; box bounds: 25th-75th percentile), 1.5 × IQR (whiskers), and values beyond 1.5 × IQR as outliers. (**C**) Hierarchical clustering of the cell types based on the top 100 features selected by Hydra for 20 cell types using the subsampled dataset derived from the Tabula Muris dataset. The dataset comprises ten major cell types and ten minor cell types, with an imbalance ratio of major to minor cells of 100:2. (**D**) t-SNE projection of the Tabula Muris dataset highlighting the log-normalized expression of the top five genes selected by Hydra for the kidney PCT epithelial cell type. Additionally, ridge line plots show the specific log-normalized expression of each top gene in kidney PCT epithelial cells compared to all other cell types. (**E**) Heatmap of log-normalized expression illustrates the cell type specificity of the top 100 genes selected by different methods using the subsampled datasets derived from the Mouse Cell Atlas. The dataset comprises ten major cell types and ten minor cell types, with an imbalance ratio of major to minor cells of 100:2. Columns correspond to cells, and rows represent individual genes specific to their respective cell types. (**F**) The overlap of top genes (*n* = 100, 200, 500, 1000) selected by different methods is quantified using the Jaccard index for four cell types, including kidney PCT epithelial cell, promonocyte, adventitial cell, and fibroblast. Source data are available online for this figure.

Reliable identification of cell type markers requires feature importance estimates to be consistent across dataset perturbations. To assess whether the ensemble modeling approach implemented in Hydra is useful for improving the stability of feature selection results, we evaluated ensemble sizes of 10, 25, 50, and 100 and compared their stability to that of a single model where no ensemble is used. Specifically, we performed stratified subsampling based on original cell type proportions using two independent single-cell transcriptomic (scRNA-seq) lung datasets (Consortium et al, 2022; Madissoon et al, 2019) to create different variants of the original datasets. We then performed feature selection using the above models and computed pairwise Pearson correlation coefficients of feature importance from these variants for each dataset. These correlations were used to measure the stability of the features selected. We found that the ensemble implementation of Hydra demonstrated a higher feature selection stability compared to the single model. Specifically, we did not observe any significant improvement in stability beyond an ensemble size of 25 (Fig. 2A,B; Appendix Fig. S1). Therefore, we selected an ensemble size of 25 as the default configuration for all subsequent analyses (we refer to this configuration simply as “Hydra" throughout the manuscript). In addition, we compared Integrated Gradients against three alternative attribution approaches, mainly, Saliency (Simonyan et al, 2013), GradientSHAP (Lundberg and Lee, 2017), and DeepLIFT (Shrikumar et al, 2017) using the lung dataset (Madissoon et al, 2019). While all four methods showed comparable cell type prediction performance, Integrated Gradients consistently demonstrated higher feature stability and lower variability across all cell types (Appendix Fig. S2). Given the importance of identifying stable and reproducible cell type markers for real-world applications, we adopted Integrated Gradients as the default feature attribution approach in Hydra.

### Capturing cell type-specific markers

We created a subsampled dataset from the scRNA-seq Mouse Cell Atlas (Schaum et al, 2018) consisting of 20 randomly selected cell types with a major-to-minor cell type sample imbalance ratio of 100:2. We then performed feature selection on this dataset using Hydra and three other statistical methods—Welch’s *t* test, Wilcoxon rank-sum test, and Limma-Voom (“Methods”). For each method, we computed Pearson correlations for the top 100 features from each cell type and performed hierarchical clustering. We found that the features selected by Hydra grouped biologically similar cell types together and exhibited strong correlations (Fig. 2C; Appendix Fig. S3). However, the other three methods, including *t* test, Wilcoxon, and Limma-voom, failed to group naive B-cell and late pro B-cell together. As another example, Limma-voom and Wilcoxon failed to group classical monocytes and pro-monocytes together.

To demonstrate Hydra’s ability to select cell type-specific features, we performed feature selection on the same subsampled dataset created earlier. Initially, we focused on one cell type—kidney proximal convoluted tubule (PCT) epithelial cells. We selected the top five genes (GPX3, TIMP3, FTH1, GATM, and CHCHD10) identified by Hydra for this cell type and visualized the expression of these genes in the original Mouse Cell Atlas. We found that all five genes were highly expressed in kidney PCT epithelial cells compared to all other cell types, indicating cell type specificity (Fig. 2D). Similarly, we selected and evaluated the expression of the top 100 genes for all 20 cell types from the subsampled dataset. We observed that Hydra displayed a clear diagonal pattern of expression in the heatmap, indicating strong specificity with minimal expression in other cell types (Fig. 2E). While the *t* test, Wilcoxon test, and Limma-Voom also demonstrated similar patterns, the top genes selected by these methods for a few cell types also exhibited expression in non-diagonal blocks.

Next, we assessed the overlap of the top genes selected by different methods for four example cell types—Kidney PCT epithelial cell, Promonocyte, Adventitial cell, and Fibroblast. Specifically, we computed the Jaccard index for the overlap of the top 100, top 200, top 500, and top 1000 genes. Overall, we found that Hydra selected more distinct genes and had a lower overlap with the top genes selected by other methods. In contrast, all statistical methods selected similar sets of genes, as indicated by a higher Jaccard index (Fig. 2F). Finally, to evaluate whether features selected by Hydra capture biologically meaningful molecular processes, we performed gene ontology enrichment analysis for three representative cell types—naive B cells, neutrophils, and mesenchymal cells. Specifically, we used the GSEApy implementation of Enrichr (Fang et al, 2023; Kuleshov et al, 2016) on the top 100 features from each cell type and tested for enrichment (Appendix Fig. S4). For naive B cells, some of the relevant enriched terms included B-cell activation, B-cell receptor signaling pathway, and regulation of B-cell proliferation, consistent with their adaptive immune functions. For neutrophils, enriched terms included neutrophil migration, neutrophil chemotaxis, inflammatory response, and granulocyte chemotaxis, reflecting innate immune activation processes. Mesenchymal cells showed enrichment for extracellular matrix organization, collagen fibril organization, and skeletal system development, consistent with their structural role. These results demonstrate Hydra’s ability to select functionally relevant and interpretable signatures that reflect core cell type-specific functions.

### Accurate prediction of cell types in single-cell transcriptomic data across multiple tissues

**Fig. 3:**
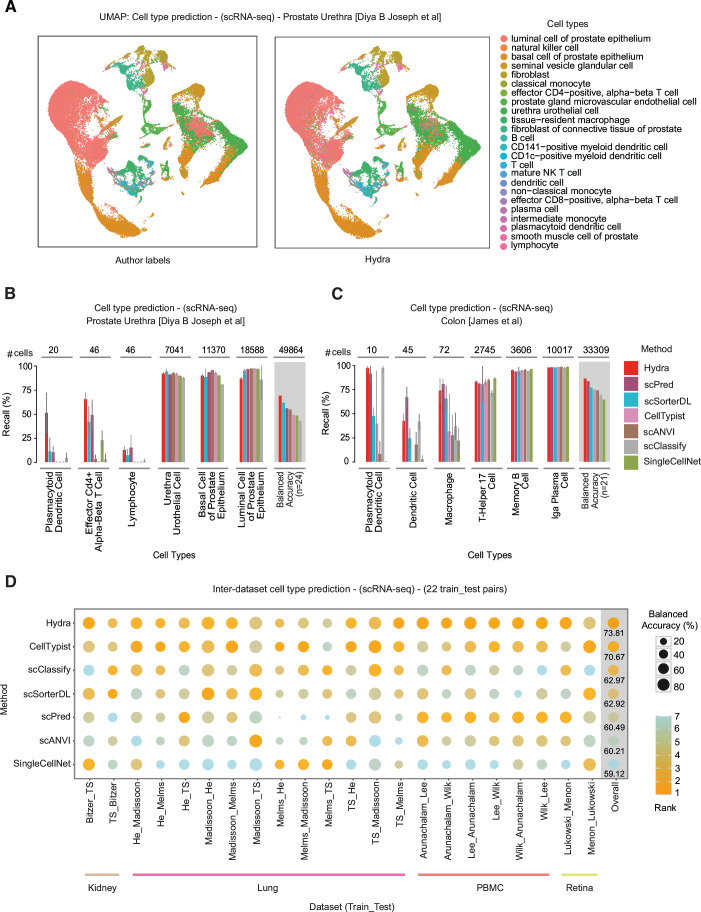
Cell-type prediction performance of Hydra against state-of-the-art methods using single-cell transcriptomic datasets. (**A**) Uniform Manifold Approximation and Projection (UMAP) plots for the single-cell transcriptomic prostate dataset, comprising 24 cell types and approximately 49,000 cells. The left panel shows the original labels as provided by the authors of the study, while the right panel shows the cell types predicted by Hydra. (**B**) Bar plot illustrating the five-time repeated random subsampling intra-dataset cell type prediction performance of various methods using the single-cell transcriptomic prostate dataset. The three smallest and three largest cell types are displayed, arranged in order of increasing sample count from left to right, followed by the overall performance across all cell types in the dataset (highlighted with a gray background). Error bars represent the standard deviation across five repeated subsampling runs. (**C**) Bar plot representing the five-time repeated random subsampling intra-dataset cell type prediction performance of different methods using a single-cell transcriptomic colon dataset comprising approximately 33,000 cells. The three smallest and three largest cell types are displayed, arranged in order of increasing sample count from left to right, followed by the overall performance across all 21 cell types in the dataset (highlighted with a gray background). Error bars represent the standard deviation across five repeated subsampling runs. (**D**) Bubble plot illustrating the inter-dataset cell type prediction performance of various methods across four tissues (kidney, lung, PBMC, and retina) and 22 train-test pairs. Methods are ranked based on their overall balanced accuracy across all train-test pairs. Source data are available online for this figure.

We next evaluated the performance of Hydra in identifying cell types across a variety of single-cell transcriptomic datasets. We utilized a total of 13 scRNA-seq datasets of varying sizes and numbers of cell types across different platforms and tissues (Appendix Table S1). We compared Hydra against six other methods commonly used for predicting cell types in scRNA-seq data - scPred (Alquicira-Hernandez et al, 2019), scSorterDL (Bai et al, 2025), CellTypist (Domínguez Conde et al, 2022), SingleCellNet (Tan and Cahan, 2019), scClassify (Lin et al, 2020), and scANVI (Xu et al, 2021). To this end, we adopted intra-dataset and inter-dataset analyses to comprehensively assess the prediction performance of methods. To account for noise, we filtered out genes expressed in less than 1% of the cells in each dataset. All methods were implemented with default parameters following the author’s official documentation. For intra-dataset cell type prediction, we conducted five times repeated random subsampling validation using two datasets—Prostate Urethra (24 cell types, query size = 49,000 cells) (Joseph et al, 2020) and Colon (24 cell types, query size = 33,000 cells) (James et al, 2020). We found that Hydra achieved the highest balanced accuracy across both datasets, 68.86% and 86.90%, respectively, outperforming all other methods (Fig. 3A–C; Appendix Figs. S5-S6). Hydra achieved a macro F1-score of 63.23% on the Prostate Urethra dataset, comparable to the top-performing method—scSorterDL (63.61%). On the Colon dataset, Hydra achieved an F1-score of 86.00% while the top-performing method scPred achieved 87.38%. For macro precision, scPred demonstrated the highest performance on both datasets (73.86% and 91.78%, respectively), while Hydra achieved 61.97% and 86.01%, respectively.

In real-world scenarios, data often come from different sources, which introduces variability, and evaluations based on splitting a dataset into train and test sets may not fully capture the generalizability of the methods. To address this, we extended our analyses to inter-dataset cell type prediction tasks where we train and test on independent datasets (e.g., batch, studies). In total, we used 10 different scRNA-seq datasets for inter-dataset benchmarking across 22 train-test pairs and 4 different tissues, including Kidney, Lung, PBMC and Retina (Arunachalam et al, 2020; Bitzer et al; Consortium et al, 2022; He et al, 2022; Jin et al, 2021; Lukowski et al, 2019; Lee et al, 2020; Melms et al, 2021; Menon et al, 2019; Madissoon et al, 2019; Program et al, 2025; Wilk et al, 2020) (Appendix Table S1). We found that Hydra achieved the highest overall balanced accuracy (73.81%) and macro F1-score (66.77%), outperforming all other methods (Fig. 3D; Appendix Fig. S7). scPred demonstrated the highest precision (77.97%), while Hydra ranked third (72.86%). However, scPred achieved a lower balanced accuracy (60.49%) and macro F1-score (61.31%) in comparison to Hydra. Taken together, these results demonstrate that Hydra achieves consistently high performance across multiple evaluation metrics, often comparable or outperforming other state-of-the-art methods.

### Joint learning and annotation in diverse single-cell multiome datasets

Hydra’s capabilities also extend to joint learning and automated annotation of single-cell multiome datasets, and can simultaneously handle three different single-cell modalities, including transcriptomic, chromatin accessibility, and protein expression data. To evaluate Hydra’s performance in this context, we utilized datasets generated from seven single-cell multiome technologies across various tissues, such as PBMC, brain, skin, kidney, and embryo (Argelaguet et al, 2022; Cao et al, 2018; Chen et al, 2019; Ma et al, 2020; Ramaswamy et al, 2021; Swanson et al, 2021; Stephenson et al, 2021) (Appendix Table S1). We compared Hydra against seven recent methods also developed for handling single-cell multiome data, including MOFA+ (Argelaguet et al, 2020), UMINT (Maitra et al, 2023), scMoMaT (Zhang et al, 2023), scGLUE (Cao and Gao, 2022), scJoint (Lin et al, 2022), totalVI (Gayoso et al, 2021), and MultiVI (Ashuach et al, 2023). For methods that primarily provide integrated embeddings, additional classifiers were trained on these embeddings to assign cell types in the query datasets.

**Fig. 4:**
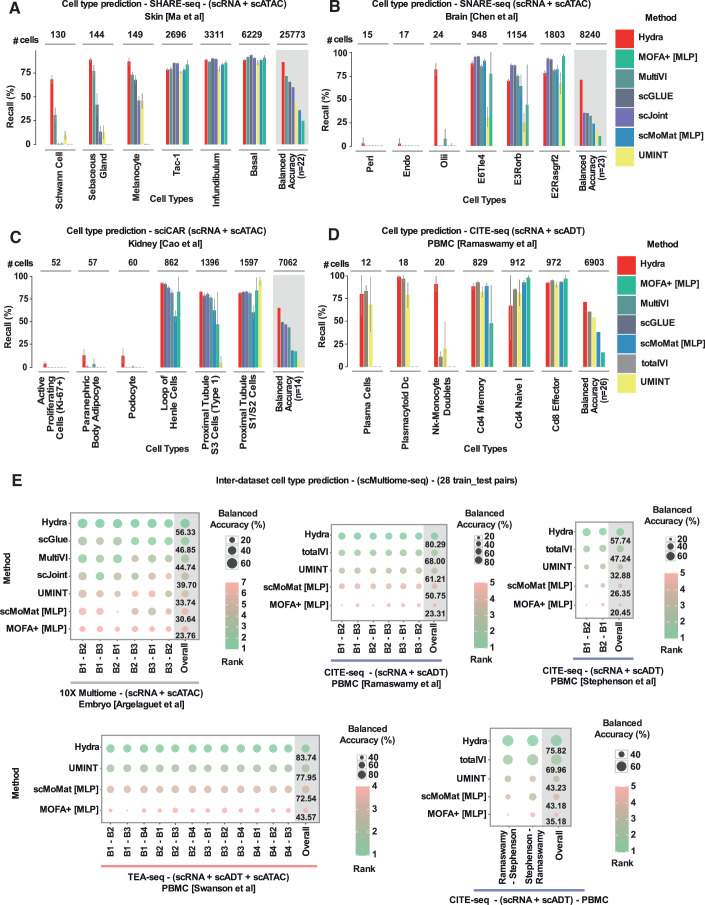
Evaluation of cell-type prediction performance of Hydra using single-cell multiome datasets. (**A**) Bar plot illustrating the five-time intra-dataset cell type prediction performance of Hydra against existing methods using the SHARE-seq (Simultaneous High-throughput ATAC and RNA Expression with sequencing) Skin dataset, comprising approximately 25,000 cells. Prediction accuracy for the three smallest and three largest cell types is displayed, followed by the overall performance across all 22 cell types in the dataset (highlighted with a gray background). Error bars represent the standard deviation across five repeated subsampling runs. (**B**) Five-time repeated random subsampling intra-dataset cell type prediction accuracy of Hydra against existing methods using the SNARE-seq (Single-Nucleus Chromatin Accessibility and mRNA Expression sequencing) Brain dataset, comprising approximately 8000 cells. The overall prediction performance across all 23 cell types is indicated with a gray background. Error bars represent the standard deviation across five repeated subsampling runs. (**C**) Comparison of five-time repeated random subsampling intra-dataset cell type prediction accuracy of Hydra against other methods using the sciCAR-seq (Single-Cell Combinatorial Indexing chromatin accessibility and RNA sequencing) Kidney dataset, comprising ~7000 cells. Overall performance across all 14 cell types in the dataset is highlighted with a gray background. Error bars represent the standard deviation across five repeated subsampling runs. (**D**) Five-time repeated random subsampling intra-dataset cell type prediction performance using the CITE-seq (Cellular Indexing of Transcriptomes and Epitopes by sequencing) PBMC dataset, comprising ~6000 cells. The overall performance across all 26 cell types in the dataset is highlighted with a gray background. Error bars represent the standard deviation across five repeated subsampling runs. (**E**) Visualization of the inter-dataset cell type prediction performance of various methods across four distinct single-cell multiome studies: 10X Multiome (10X Chromium Epi Multiome ATAC + Gene Expression), CITE-seq, and TEA-seq (Transcriptome, Epitope, and Accessibility sequencing), spanning 28 train-test splits. The bubble plot ranks methods based on their overall performance. Source data are available online for this figure.

For the intra-dataset cell type identification task, we performed five-time repeated random subsampling validation on datasets obtained from four different single-cell multiome technologies, mainly Simultaneous High-throughput ATAC and RNA Expression with sequencing (SHARE-seq), Single-Nucleus Chromatin Accessibility and mRNA Expression sequencing (SNARE-seq), Single-Cell Combinatorial Indexing chromatin accessibility and RNA sequencing (sciCAR), and Cellular Indexing of Transcriptomes and Epitopes by sequencing (CITE-seq) (Appendix Table S1). Overall, Hydra achieved the highest balanced accuracy and macro F1 score across all datasets (Fig. 4A–D; Appendix Figs. S8–S11). For most datasets, Hydra demonstrated the highest macro precision, while totalVI achieved the highest precision for PBMC CITE-seq dataset (Ramaswamy et al, 2021). Further, we conducted inter-dataset benchmarking involving 28 train-test splits (Appendix Table S1). This evaluation included datasets generated from simultaneous profiling of two modalities from CITE-seq (scRNA+scADT) and 10X Chromium Epi Multiome ATAC + Gene Expression - 10X Multiome (scRNA+scATAC) technolgies as well as all three modalities from Transcriptome, Epitope, and Accessibility sequencing—TEA-seq (scRNA+scATAC+scADT) technology. Again, Hydra outperformed other state-of-the-art methods with the highest balanced accuracy and macro F1 score (Fig. 4E; Appendix Fig. S12). Hydra achieved the highest macro precision in most datasets, except CITE-seq PBMC datasets (Ramaswamy et al, 2021; Stephenson et al, 2021), where totalVI ranked first, with Hydra demonstrating comparable performance. The superior performance of Hydra across both single-cell transcriptomic and single-cell multiome datasets can be attributed to its ability to effectively identify rare cell populations, which remain a key challenge for existing approaches.

### Hydra robustly maps cellular subtypes in Alzheimer’s disease from single-cell multiome data

**Fig. 5:**
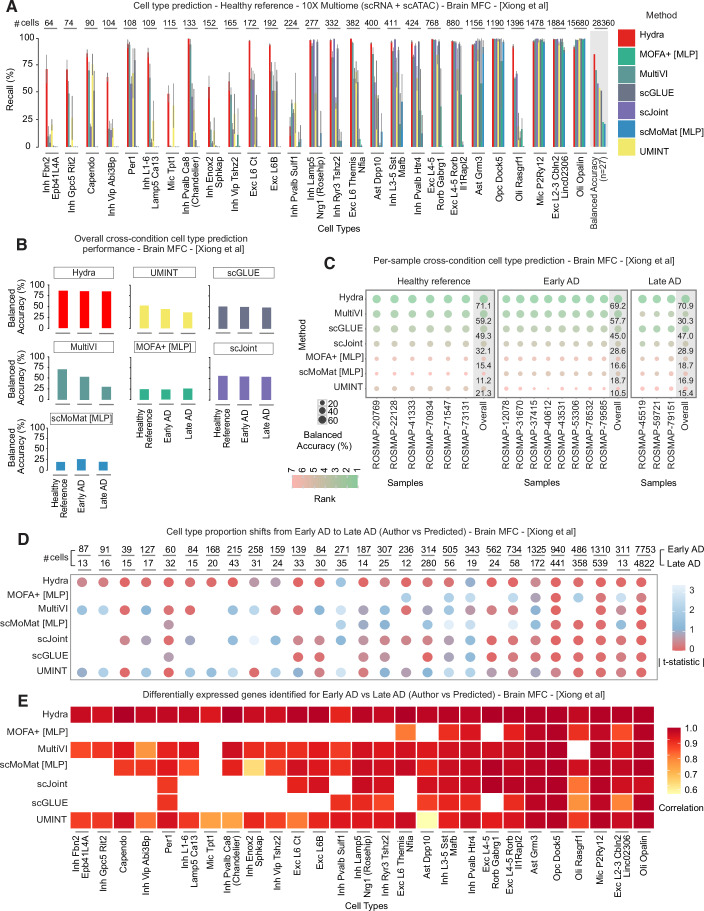
Mapping cellular subtypes of the brain medial frontal cortex (MFC) in Alzheimer’s disease. (**A**) Bar plot illustrating the five time intra-dataset cell type prediction performance of Hydra against different single-cell multiome methods using the healthy medial frontal cortex (MFC) brain dataset. This dataset profiles RNA and ATAC modalities and comprises approximately 28,000 cells across 27 cell types. Prediction performance is displayed for all cell types, followed by the overall performance across all cell types in the dataset (highlighted with a gray background). Cell types are arranged in the order of increasing sample count from left to right. Error bars represent the standard deviation across five repeated subsampling runs. (**B**) Faceted bar plots showing overall cell type prediction performance of different single-cell multiome methods across conditions trained on a healthy reference dataset, stratified by original cell type proportions. Error bars represent the variability in prediction accuracy across individual cell types. (**C**) Bubble plot illustrating per-sample cross-condition identification of cell types using different single-cell multiome methods trained on healthy samples. The plot is faceted by conditions namely—healthy reference comprising held-out healthy samples, early- and late-stage Alzheimer’s disease held-out samples. Methods are ranked based on overall performance across all conditions. (**D**) Bubble plot showing absolute t-statistics quantifying cell type proportion changes from early to late stages of Alzheimer’s disease. Each circle represents the magnitude of proportion shift differences between the author label and the predicted label for each cell type. (**E**) Heatmap illustrating Spearman correlation coefficients between differential gene expression t-statistics derived from author annotations and method predictions for early versus late AD. Source data are available online for this figure.

In complex conditions, such as Alzheimer’s disease (AD), significant changes in molecular profiles can often obscure cell identities by altering genetic and epigenetic landscapes. Such changes can make it challenging to accurately identify and distinguish cell types, especially when relying on markers established from a healthy reference. This challenge is particularly acute in the brain, where many neuronal and glial subpopulations share highly similar molecular signatures. Here, we aimed to evaluate Hydra’s robustness in learning from a healthy reference dataset and predicting cellular subtypes across conditions, particularly early- and late-stage Alzheimer’s disease cohorts. To achieve this, we utilized our previously published single-cell multiome dataset that profiled the transcriptome and epigenome of the medial frontal cortex (MFC) region of the brain (Xiong et al, 2023). First, we used an intra-dataset cell type identification approach described earlier using a healthy MFC brain dataset consisting of 27 distinct cell populations, and compared the prediction performance of Hydra against other single-cell multiome methods. We found that Hydra outperformed other methods in predicting both major and minor cell types of the brain with a balanced accuracy of ~85% (Fig. 5A). Hydra also achieved the highest macro F1 score and macro precision in this evaluation (Appendix Fig. S13). While most other methods performed reasonably well in identifying certain major cell types, their performance decreased as the size of the specialized cell populations decreased (Fig. 5A).

To further evaluate the robustness of methods, we conducted a cross-condition cell type prediction. Essentially, we trained each method on a subset of the healthy reference dataset stratified by original cell type proportions and used the trained models to predict cell types in held-out healthy reference, early- and late-stage Alzheimer’s disease datasets. Remarkably, Hydra accurately transferred annotations from the healthy reference and identified most cell types in early- and late-stage Alzheimer’s disease, with a balanced accuracy of approximately 85% and 84%, respectively. In contrast, the performance of MultiVI and UMINT decreased in early- and late-stages of Alzheimer’s disease, while other methods demonstrated mostly consistent, but lower performance in comparison to Hydra (Fig. 5B; Appendix Fig. S14). Notably, similar to the intra-dataset evaluation scenario, the benchmarked baseline scMultiome methods failed to effectively identify smaller cell populations in disease conditions. Next, to further evaluate the generalizability across disease-specific sample variations and to demonstrate cross-condition cell type prediction on unseen samples, we withheld a subset of healthy samples and trained the methods on the remaining healthy cohort. We then applied the trained models to predict cell types in held-out healthy samples as well as in the early- and late-stage of Alzheimer’s disease. We observed that the performance of Hydra decreased slightly but still achieved a considerably high cell type prediction performance across all conditions in comparison to other methods (Fig. 5C). These results show Hydra’s ability to reliably identify cell type markers from healthy samples and demonstrate its robustness to molecular-level shifts occurring in disease conditions.

Finally, to demonstrate that the predicted cell types capture biologically meaningful changes characteristic of Alzheimer’s disease progression, we performed two complementary analyses comparing cell type proportion shifts and differential gene expression patterns between early- and late-stages of AD. First, we quantified cell type proportion changes from early to late AD using sample-level t-statistics and compared the magnitude of changes captured by author annotations with individual method predictions across all cell types. Hydra demonstrated consistent performance in capturing proportion shifts across the majority of cell types, with UMINT, MultiVI, and scJoint showing moderate performance in the identified cell types. In contrast, scGLUE, scMoMat and MOFA+ showed substantial gaps, particularly failing to predict several low-abundance cell types, highlighting their limited ability to transfer annotations to rare but potentially important cellular subtypes in disease contexts (Fig. 5D). Subsequently, we assessed whether predicted cell types preserve disease-relevant transcriptional signatures by performing pseudobulk differentially expressed gene (DEG) analysis between early- and late-stages of AD. We calculated Spearman correlations between DEG t-statistics derived from author annotations and those from individual method predictions for each cell type. We found that Hydra outperformed other methods in maintaining strong correlations with author DEG signatures in the identified cell types, including rare cell populations, demonstrating its ability to preserve critical disease-relevant molecular signatures (Fig. 5E).

### Computational resource analysis

**Fig. 6:**
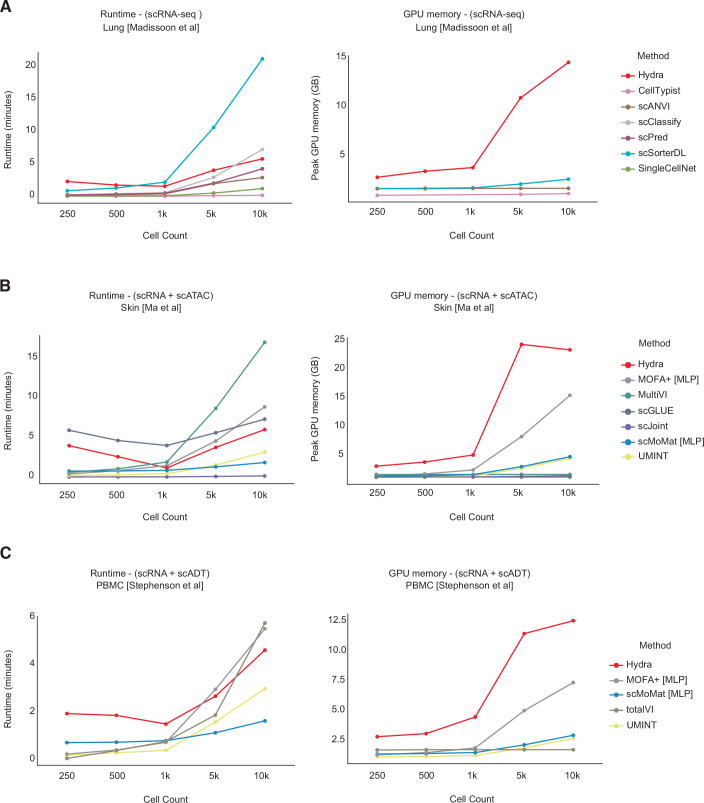
Computational resources analysis. (**A**–**C**) Line plots showing runtime (left panel) and GPU consumption (right panel) of different methods across varying cell counts (250, 500, 1000, 5000, 10000). Evaluations were performed on single-cell transcriptomic data (**A**), joint profiling of single-cell transcriptomic and chromatin accessibility data (**B**) and joint profiling of single-cell transcriptomic and surface protein data (**C**). Source data are available online for this figure.

We evaluated the computational cost of running the ensemble framework of Hydra by measuring runtime and GPU memory consumption across varying dataset sizes (n_cells = 250, 500, 1000, 5000, 10,000) and compared it against baseline methods for both single-cell transcriptomic (Madissoon et al, 2019) and single-cell multiome data (Ma et al, 2020; Stephenson et al, 2021). Across all three benchmarked datasets, Hydra completed training in under 10 min, indicating that our ensemble framework can be efficiently deployed with standard computational resources (Fig. 6A–C). We found that for most methods, runtime scaled gradually as cell count increased from 250 to 10,000 cells. Specifically, for single-cell transcriptomic data, scSorterDL exhibited longer runtimes from 1000 cells onward. For joint profiling of single-cell transcriptome and chromatin accessibility data, MultiVI required longer execution times at larger dataset sizes. For joint profiling of single-cell transcriptome and surface protein data, runtime performance varied across methods, with all methods completing training in under 10 min. Regarding GPU memory consumption, Hydra exhibited higher peak memory usage compared to baseline methods across all three datasets. This can be attributed to its ensemble architecture, which trains multiple variational autoencoder models to perform feature selection and improve prediction performance. Taken together, Hydra’s computational cost remains within practical limits and demonstrates its scalability for applications in single-cell omics analysis.

## Discussion

In this paper, we introduced Hydra as an interpretable deep generative ensemble learning framework designed to automate the process of identifying cell type-specific features and predicting cell types in both unimodal and multimodal single-cell omics datasets. By integrating multiple variational autoencoders, Hydra generates new samples from learned probability distributions to efficiently mitigate class imbalance and ensure that all cell types, including rare/minor cell populations, are adequately represented. This approach preserves essential biological signals during model training and enables Hydra to robustly capture cellular markers, especially for rare cell types. Further, the multimodal capability of Hydra represents methodological flexibility for handling diverse single-cell modalities (RNA, ADT, or ATAC) in a unified architecture, where available. The feature selection module automatically prioritizes the most informative signals across modalities to identify cell types.

Hydra attempts to address the challenge of interpretability of traditional deep learning models through post hoc feature attribution via Integrated Gradients (Sundararajan et al, 2017). We note that with post hoc interpretability, additional analyses are performed on trained neural networks to explain the model. This differs from intrinsic interpretability, where prior knowledge or biologically interpretable designs are directly incorporated into the model architecture (Lipton, 2018; Murdoch et al, 2019; Wagle et al, 2024). Further, post hoc feature attribution techniques identify the most important features influencing predictions and not how features interact within the network to produce final outputs. Thus, the attribution approach employed in Hydra provides a ranked list of biologically meaningful cell type-specific features rather than complete mechanistic explanations of the model’s internal computations. Nevertheless, these features retain practical utility for distinguishing cell populations, and the ensemble deep learning approach further improves feature stability.

We show that Hydra consistently offers superior performance compared to existing methods across 21 datasets, covering a wide range of single-cell omics modalities and tissues. Furthermore, our evaluation strategy emphasizes predictive performance for each individual cell type, which is particularly important for identifying minor cell populations often overlooked by more generalized global metrics. Finally, we demonstrate Hydra’s ability to facilitate robust reference-to-query cell type mapping in both normal and disease contexts, including independent inter-dataset tasks across different sequencing protocols.

Although Hydra demonstrates robust performance across diverse datasets and biological conditions, we acknowledge limitations inherent to its supervised framework and evaluation. First, the model is restricted to predicting cell types present in the training reference and does not currently support the discovery of novel or unannotated cell populations in query datasets. Second, as with most supervised tools, Hydra relies on the quality of input cell type labels in the reference dataset, and significant label noise could potentially affect its prediction performance. Future extensions of the framework would therefore focus on integrating approaches to detect unseen cell populations and conduct systematic sensitivity analyses to address label noise. Finally, in our single-cell multiome evaluations, the ground-truth labels are primarily derived from transcriptomic data. This can bias performance assessments as models that genuinely integrate additional modalities may deviate from such RNA-centric labels, potentially reducing apparent annotation accuracy. Thus, developing annotation strategies that incorporate multimodal reference labels would enable more rigorous evaluation of these integration methods.

As single-cell multiomics technologies become increasingly accessible, we anticipate that Hydra will serve as a valuable tool to harness such rich, multilayered information and shed light on underlying cellular heterogeneity.

## Methods

**Table:** Reagents and tools table

Reagent/resource	Reference or source	Identifier or catalog number
**Experimental models**		
**Recombinant DNA**		
**Antibodies**		
**Oligonucleotides and other sequence-based reagents**		
**Chemicals, enzymes, and other reagents**		
**Software**		
SingleCellNet	https://cahanlab-pysinglecellnet.readthedocs-hosted.com/	NA
scClassify	https://github.com/SydneyBioX/scClassify	NA
scPred	https://powellgenomicslab.github.io/scPred/	NA
scANVI	https://docs.scvi-tools.org/	NA
scSorterDL	https://github.com/kellen8hao/scSorterDL	NA
CellTypist	https://celltypist.readthedocs.io/	NA
UMINT	https://github.com/deeplearner87/UMINT	NA
MOFA+	https://biofam.github.io/MOFA2/tutorials.html	NA
scMoMat	https://github.com/PeterZZQ/scMoMaT	NA
scGLUE	https://scglue.readthedocs.io/en/latest	NA
scJoint	https://github.com/SydneyBioX/scJoint	NA
totalVI	https://docs.scvi-tools.org/	NA
MultiVI	https://docs.scvi-tools.org/	NA
GSEApy	https://gseapy.readthedocs.io/	NA
**Other**		

### Data processing and normalization

The input for Hydra can include both unimodal and multimodal single-cell data. We start with a count matrix *M* of dimensions *n* × *m*, where *n* denotes the number of cells and *m* denotes the number of features. To reduce noise and retain biologically relevant features, we filter out features *f* if the proportion of cells for which *f*_*i*_ has a zero count is greater than or equal to 99%. Feature *f*_*i*_ is retained if

$$\frac{\,{{{\rm{Number}}}}\; {{{\rm{of}}}}\; {{{\rm{zero}}}}\; {{{\rm{counts}}}}\; {{{\rm{in}}}}\,{f}_{i}}{n} \; < \; 0.99.$$

After filtering, the count matrix *M* is logarithmically transformed. Subsequently, the matrix is scaled and transformed into a PyTorch tensor for model input. All datasets used for benchmarking in this study were represented at the gene-level.

$${M}_{ij}^{{\prime} }={\log }_{2}\left({M}_{ij}+1\right).$$

### Feature ranking module

#### Model architecture

To capture the underlying heterogeneity in the single-cell data, we employ a variational autoencoder architecture with multiple cell-type classification heads in the initial training phase of the feature ranking module. For single-cell transcriptomic (RNA) and chromatin accessibility (ATAC), the encoder and decoder architecture is consistent with a latent space **z**_*i*_ of 100 neurons. In the case of single-cell surface proteins (ADT), we use fewer neurons in the hidden layers due to lower feature dimensionality. The single-cell multiome data adopts a similar architecture to unimodal data, but modality-specific encoder layers process each modality independently before concatenating and passing them through a shared latent space **z**_*i*_ of 100 neurons. This joint learning allows Hydra to capture information embedded in different single-cell modalities. For each cell *i*, let ${{{{\bf{x}}}}}_{i}^{\,{{{\rm{RNA}}}}}$, ${{{{\bf{x}}}}}_{i}^{\,{{{\rm{ATAC}}}}}$, and ${{{{\bf{x}}}}}_{i}^{\,{{{\rm{ADT}}}}}$ denote the feature vectors for RNA, ATAC, and ADT modalities, respectively, where available. Each modality has feature dimensionality *d*_RNA_, *d*_ATAC_, or *d*_ADT_.

For unimodal single-cell transcriptomic data, the input ${{{{\bf{x}}}}}_{i}^{\,{{{\rm{RNA}}}}}$ is first projected through a linear layer with weight matrix ${{{{\bf{W}}}}}_{\,{{{\rm{enc}}}}}^{{{{\rm{RNA}}}}}\in {{\mathbb{R}}}^{{h}_{{{{\rm{RNA}}}}}\times {d}_{{{{\rm{RNA}}}}}}$ and bias vector ${{{{\bf{b}}}}}_{\,{{{\rm{enc}}}}}^{{{{\rm{RNA}}}}}\in {{\mathbb{R}}}^{{h}_{{{{\rm{RNA}}}}}}$, followed by Mish activation (Misra) and batch normalization (BN) (Ioffe and Szegedy), with a dropout rate of 0.2. This is followed by a second linear transformation to reduce the dimensionality to the latent space *z*. The reparameterization trick (Kingma and Welling) is applied to sample from *z* during optimization.

$${{{{\bf{h}}}}}_{i}^{\,{{{\rm{RNA}}}}}={{{{\rm{Dropout}}}}}_{0.2}\left({{{\rm{BN}}}}\,\left({{{\rm{Mish}}}}\,({{{{\bf{W}}}}}_{\,{{{\rm{enc}}}}}^{{{{\rm{RNA}}}}}{{{{\bf{x}}}}}_{i}^{\,{{{\rm{RNA}}}}}+{{{{\bf{b}}}}}_{\,{{{\rm{enc}}}}}^{{{{\rm{RNA}}}}})\right)\right)$$

For single-cell multiome data, Hydra can handle up to three modalities (e.g., RNA, ATAC, and ADT) by employing modality-specific encoders and decoders. For a dataset simultaneously profiling all three modalities: ${{{{\bf{x}}}}}_{i}=[{{{{\bf{x}}}}}_{i}^{\,{{{\rm{RNA}}}}};{{{{\bf{x}}}}}_{i}^{\,{{{\rm{ATAC}}}}};{{{{\bf{x}}}}}_{i}^{\,{{{\rm{ADT}}}}}]\in {{\mathbb{R}}}^{{d}_{{{{\rm{RNA}}}}}+{d}_{{{{\rm{ATAC}}}}}+{d}_{{{{\rm{ADT}}}}}}$, where ${{{{\bf{x}}}}}_{i}^{\,{{{\rm{RNA}}}}}\in {{\mathbb{R}}}^{{d}_{{{{\rm{RNA}}}}}}$, ${{{{\bf{x}}}}}_{i}^{\,{{{\rm{ATAC}}}}}\in {{\mathbb{R}}}^{{d}_{{{{\rm{ATAC}}}}}}$, and ${{{{\bf{x}}}}}_{i}^{\,{{{\rm{ADT}}}}}\in {{\mathbb{R}}}^{{d}_{{{{\rm{ADT}}}}}}$ represent the feature vectors for RNA, ATAC, and ADT modalities, respectively. The input is processed by modality-specific encoders *E*^RNA^, *E*^ATAC^, and *E*^ADT^:

$${{{\bf{h}}}}_{i}^{\,{{\rm{RNA}}}} = 	 \; {E}^{{{\rm{RNA}}}}({{{\bf{x}}}}_{i}^{\,{{\rm{RNA}}}})\\ = 	 \; {{{\rm{Dropout}}}}_{0.2}\left({{\rm{BN}}}\,\left({{\rm{Mish}}}\,({{{\bf{W}}}}_{\,{{\rm{enc}}}}^{{{\rm{RNA}}}}{{{\bf{x}}}}_{i}^{\,{{\rm{RNA}}}}+{{{\bf{b}}}}_{\,{{\rm{enc}}}}^{{{\rm{RNA}}}})\right)\right)\\ {{{\bf{h}}}}_{i}^{\,{{\rm{ATAC}}}} = 	 \;{E}^{{{\rm{ATAC}}}}({{{\bf{x}}}}_{i}^{\,{{\rm{ATAC}}}})\\ = 	 \;{{{\rm{Dropout}}}}_{0.2}\left({{\rm{BN}}}\,\left({{\rm{Mish}}}\,({{{\bf{W}}}}_{\,{{\rm{enc}}}}^{{{\rm{ATAC}}}}{{{\bf{x}}}}_{i}^{\,{{\rm{ATAC}}}}+{{{\bf{b}}}}_{\,{{\rm{enc}}}}^{{{\rm{ATAC}}}})\right)\right)\\ {{{\bf{h}}}}_{i}^{\,{{\rm{ADT}}}} = 	 \; {E}^{{{\rm{ADT}}}}({{{\bf{x}}}}_{i}^{\,{{\rm{ADT}}}})\\ = 	 \; {{{\rm{Dropout}}}}_{0.2}\left({{\rm{BN}}}\,\left({{\rm{Mish}}}\,({{{\bf{W}}}}_{\,{{\rm{enc}}}}^{{{\rm{ADT}}}}{{{\bf{x}}}}_{i}^{\,{{\rm{ADT}}}}+{{{\bf{b}}}}_{\,{{\rm{enc}}}}^{{{\rm{ADT}}}})\right)\right)$$

The modality-specific representations are then concatenated and processed through a fully connected layer to create the shared latent variable **z**_*i*_ for joint learning. In the decoder, **z**_*i*_ is passed through modality-specific decoders *D*^RNA^, *D*^ATAC^, and *D*^ADT^, where each modality undergoes similar transformations to reconstruct the original inputs.

$${\widehat{{{\bf{x}}}}}_{i}^{\,{{\rm{RNA}}}} = 	 \;{D}^{{{\rm{RNA}}}}({{{\bf{z}}}}_{i})\\ = 	 \;{{{\rm{Dropout}}}}_{0.2}\left({{\rm{BN}}}\,\left({{\rm{Mish}}}\,({{{\bf{W}}}}_{\,{{\rm{dec}}}}^{{{\rm{RNA}}}}{{{\bf{z}}}}_{i}+{{{\bf{b}}}}_{\,{{\rm{dec}}}}^{{{\rm{RNA}}}})\right)\right)\\ {\widehat{{{\bf{x}}}}}_{i}^{\,{{\rm{ATAC}}}} = 	 \;{D}^{{{\rm{ATAC}}}}({{{\bf{z}}}}_{i})\\ = 	 \;{{{\rm{Dropout}}}}_{0.2}\left({{\rm{BN}}}\,\left({{\rm{Mish}}}\,({{{\bf{W}}}}_{\,{{\rm{dec}}}}^{{{\rm{ATAC}}}}{{{\bf{z}}}}_{i}+{{{\bf{b}}}}_{\,{{\rm{dec}}}}^{{{\rm{ATAC}}}})\right)\right)\\ {\widehat{{{\bf{x}}}}}_{i}^{\,{{\rm{ADT}}}} = 	 \;{D}^{{{\rm{ADT}}}}({{{\bf{z}}}}_{i})\\ = 	 \;{{{\rm{Dropout}}}}_{0.2}\left({{\rm{BN}}}\,\left({{\rm{Mish}}}\,({{{\bf{W}}}}_{\,{{\rm{dec}}}}^{{{\rm{ADT}}}}{{{\bf{z}}}}_{i}+{{{\bf{b}}}}_{\,{{\rm{dec}}}}^{{{\rm{ADT}}}})\right)\right)$$

where ${{{{\bf{W}}}}}_{\,{{{\rm{dec}}}}}^{{{{\rm{RNA}}}}}\in {{\mathbb{R}}}^{{d}_{{{{\rm{RNA}}}}}\times {d}_{z}}$, ${{{{\bf{W}}}}}_{\,{{{\rm{dec}}}}}^{{{{\rm{ATAC}}}}}\in {{\mathbb{R}}}^{{d}_{{{{\rm{ATAC}}}}}\times {d}_{z}}$, ${{{{\bf{W}}}}}_{\,{{{\rm{dec}}}}}^{{{{\rm{ADT}}}}}\in {{\mathbb{R}}}^{{d}_{{{{\rm{ADT}}}}}\times {d}_{z}}$ and ${{{{\bf{b}}}}}_{\,{{{\rm{dec}}}}}^{{{{\rm{RNA}}}}}$, ${{{{\bf{b}}}}}_{\,{{{\rm{dec}}}}}^{{{{\rm{ATAC}}}}}$, ${{{{\bf{b}}}}}_{\,{{{\rm{dec}}}}}^{{{{\rm{ADT}}}}}$ are modality-specific decoder weight matrices and bias vectors. The latent variable **z**_*i*_ is also fed into multiple cell-type classification heads. The number of classification heads *C*_*m*_ is user-configurable, with a default of 25 set empirically.

#### Initial training phase

During the initial training phase, the variational autoencoder and ensemble of cell type classification heads are jointly trained on the original training data. The model optimizes a loss function that takes into account both the reconstruction and classification tasks to capture the underlying heterogeneity in the single-cell data. The reconstruction loss L_recon_ consists of the mean squared error between the input *x* and the reconstructed data $\widehat{x}$, and the Kullback–Leibler divergence L_KL_ for the variational component:

$${L}_{{{{\rm{recon}}}}}\,=\,{{{\rm{MSE}}}}\left(x,\widehat{x}\right)\,+\,\frac{1}{N}\,{L}_{kl}\left(\mu ,\sigma \right)$$

Where *N* is the number of features, *μ* and *σ* are the mean and standard deviation from the encoder’s output. For the classification task, the model employs multiple cell-type classification heads *C*_*m*_ that operate on the latent space *z*. Predictions from all classification heads are averaged to obtain the final class $\bar{Y}$. The classification loss L_class_ is then computed using a label-smoothed cross-entropy function (Müller et al, 2019) between the averaged prediction $\widehat{y}$ and the true class labels *y*:

$${L}_{{{{\rm{class}}}}}={{{\rm{CrossEntropyLabelSmooth}}}}(\widehat{y},y)$$

The total loss function combines both reconstruction and classification objectives:

$${{{{\rm{L}}}}}_{{{{\rm{total}}}}}={{{{\rm{L}}}}}_{{{{\rm{recon}}}}}+\lambda {{{{\rm{L}}}}}_{{{{\rm{class}}}}}$$

where *λ* is a weighting parameter that balances the reconstruction and classification tasks. Based on prior empirical evaluation (Liu et al, 2023), we adopt *λ* = 0.9 as the default value, and this value is held constant across all datasets in our experiments. Finally, to optimize the training time, we also implement an early stopping approach with a patience of 10 epochs.

#### Refinement and feature ranking

During the refinement stage, we address class imbalance by generating balanced datasets from the original input single-cell data. We do this by categorizing cell types into major and minor groups based on the median sample size across all cell types. To create a balanced dataset, we randomly downsample the major cell types and generate additional samples for minor cell types using the original trained model. Specifically, for a minor cell type *c* with *N*_*c*_ samples (where *N*_*c*_ < *N*_median_), we generate *N*_syn_ = *N*_median_ − *N*_*c*_ synthetic samples, where *N*_median_ is the median sample size across all cell types. Synthetic cells are generated by sampling from the learned latent distribution of the variational autoencoder:

$${z}_{{{{\rm{syn}}}}} \sim {{{\mathcal{N}}}}({\mu }_{c},{\sigma }_{c}^{2}),\,{x}_{{{{\rm{syn}}}}}={{{\rm{Decoder}}}}({z}_{{{{\rm{syn}}}}})$$

where *μ*_*c*_ and ${\sigma }_{c}^{2}$ are the mean and variance of the latent representations computed from real cells of type *c*, and the decoder reconstructs the synthetic sample in the original feature space. For major cell types (where *N*_*c*_ ≥ *N*_median_), we perform random down-sampling to *N*_median_ samples. We then initialize a new model for refinement by inheriting the weights of the original model’s encoder, decoder, and a specific classification head *C*_*m*_. Specifically, we create a new model for each classification head in the original trained model. For each initialized model, we generate a new balanced dataset following the above procedure. The refined models are then trained on their respective balanced datasets for a varying number of epochs. After refining all the models, we apply a post hoc feature attribution technique, Integrated Gradients (IG), to compute importance scores for each feature in each cell type.

Integrated Gradients is an axiomatic feature attribution method designed to explain predictions of deep neural networks by quantifying the contribution of each input feature to the output. Specifically, it computes feature attributions by accumulating gradients along a straight-line path from a baseline input to the actual input. This approach satisfies two fundamental properties—(i) sensitivity, which assigns non-zero importance to features that influence predictions, and (ii) implementation invariance, which ensures consistent attributions for functionally equivalent networks (Ancona et al, 2018; Sundararajan et al, 2017). Mathematically, for an input **x**_*i*_ and baseline ${{{{\bf{x}}}}}_{i}^{{\prime} }$, the Integrated Gradients attribution for feature *k* is defined as:

$${{{{\rm{IG}}}}}_{k}({{{{\bf{x}}}}}_{i})=({x}_{i,k}-{x}_{i,k}^{{\prime} }){\int }_{\!\!\!\! \alpha =0}^{1}\frac{\partial F({{{{\bf{x}}}}}_{i}^{{\prime} }+\alpha \times ({{{{\bf{x}}}}}_{i}-{{{{\bf{x}}}}}_{i}^{{\prime} }))}{\partial {x}_{i,k}}d\alpha$$

where *α* is the interpolation parameter ranging from 0 to 1, *F* represents the output of the cell type classification head for a given cell type, and *x*_*i*,*k*_ denotes the *k*th feature of cell *i*. Using Riemann summation, the integral is approximated numerically over discrete steps along the interpolation path. To improve computational efficiency, we adopt a batch attribution approach on all cells belonging to a given cell type. For a given cell type *c* and model *m*, the feature importance scores ${{{{\boldsymbol{\theta }}}}}_{c}^{m}\in {{\mathbb{R}}}^{d}$ is computed by averaging the absolute values of the Integrated Gradients attributions over all cells of type *c*:

$${\theta }_{c,k}^{m}=\frac{1}{{N}_{c}}{\sum }_{i\in {{{{\mathcal{C}}}}}_{c}}\left|{{{{\rm{IG}}}}}_{k}({f}_{m},{{{{\bf{x}}}}}_{i},\,{{{\rm{target}}}}\,=c)\right|$$

where *N*_*c*_ is the total number of cells of type *c*, ${{{{\mathcal{C}}}}}_{c}=\{i:\,{{{\rm{cell}}}}\,i\,{{{\rm{has\; type}}}}\,c\}$ is the set of cell indices with type *c*, ${\theta }_{c,k}^{m}$ is the importance score for feature *k* in cell type *c* from model *m*, and *f*_*m*_ is the model comprising the encoder and classification head *C*_*m*_ from the refined model *m*. The features are then ranked considering the biological directionality. Finally, we obtain a consensus cell type-specific feature ranking by averaging the importance scores across all refined models. Specifically, for a given feature *k* in cell type *c*, the consensus importance score is computed as:

$${\bar{\theta }}_{c,k}=\frac{1}{M}{\sum }_{m=1}^{M}{\theta }_{c,k}^{m}$$

where ${\theta }_{c,k}^{m}$ represents the importance score of feature *k* for cell type *c* from refined model *m*, and *M* is the total number of ensemble models.

### Automated cell type annotation module

We train an ensemble of simple neural network classifiers to assign cell types in the query single-cell dataset. Each neural network classifier consists of an input layer, a hidden layer with the Mish activation function

$${{{\rm{Mish}}}}(x)=x\cdot \tanh \left(ln(1+{e}^{x})\right),$$

and an output layer. The forward pass through each classifier can be defined as:

$${{{{\bf{y}}}}}_{i}={W}_{2}(\,{{{\rm{Mish}}}}\,({W}_{1}{{{{\bf{x}}}}}_{i}+{{{{\bf{b}}}}}_{1}))+{{{{\bf{b}}}}}_{2}$$

where **x**_*i*_ is the input feature vector for cell *i*, **y**_*i*_ is the output logits corresponding to the cell type classes for cell *i*, *W*_1_ and **b**_1_ are the weights and biases of the hidden layer, and *W*_2_ and **b**_2_ are the weights and biases of the output layer. The ensemble size corresponds to the number of refined models from the feature ranking module. To train the classifiers, we generate balanced datasets using the refined models similar to the model refinement step. The dimensions of these datasets are reduced using the union of the top 100 ranked features from all cell types, as determined by the feature ranking module. Each neural network classifier is then trained on its respective balanced dataset for five epochs. After training, the ensemble of classifiers is used to map cell types in the query single-cell dataset. Predictions from all classifiers are aggregated by averaging their prediction probabilities to obtain the final cell type assignments. All datasets used in the present study is available at Appendix Table S1. A summary of default hyperparameters used in Hydra is available in Appendix Table S2.

### Running existing methods

#### Statistical tests

We employed three statistical methods, including Welch’s *t* test, Wilcoxon rank-sum test, and Limma-Voom. As a preprocessing step, genes expressed in less than 1% of cells were filtered out. For each cell type, we performed a one-vs-all analysis by comparing the expression levels of genes in the cell type of interest against all other cell types combined. For the *t* test and Wilcoxon rank-sum test, raw count matrices were log-transformed and scaled. The test statistics were computed, and genes were ranked considering the biological direction of expression changes. For the Limma-Voom analysis, we used the voom function to model the count data, followed by linear modeling and empirical Bayes moderation using the lmFit and eBayes functions from the Limma package. Cell type-specific genes were ranked based on moderated t-statistics, considering the biological direction.

#### SingleCellNet

SingleCellNet (SCN) was implemented for scRNA seq cell type identification evaluation following the official documentation (https://cahanlab-pysinglecellnet.readthedocs-hosted.com/). Genes expressed in less than 1% of cells were filtered out, and raw count matrices were converted into AnnData objects. The training data was normalized to total counts, log1p transformed, and 2000 highly variable genes were selected using the Seurat v3 method for SCN input. The model was trained using the train_classifier function with the following parameters—‘nTopGenes’ = 200, ‘nTopGenePairs’ = 200, ‘nRand’ = 100, and ‘n_comps’ = 30. Cell types in the query dataset were then predicted using the classify_anndata function.

#### scClassify

scClassify was implemented for scRNA seq cell type identification evaluation, following the official documentation (https://sydneybiox.github.io/scClassify/). Genes expressed in less than 1% of cells were filtered out, and raw count matrices were log-transformed and scaled. Model training and cell type prediction on the query dataset were performed using the scClassify function with the following parameters: ‘tree’ = “HOPACH”, ‘algorithm’ = “WKNN”, ‘selectFeatures’ = c(“limma”), and ‘similarity’ = c(“pearson”, “cosine”).

#### scPred

scPred was implemented for scRNA seq cell type identification evaluation, following the official documentation (https://powellgenomicslab.github.io/scPred/). Genes expressed in less than 1% of cells were filtered out, and raw count matrices were preprocessed using Seurat’s Butler et al (2018) NormalizeData, ScaleData, and FindVariableFeatures functions. The scPred input object was then created using the getFeatureSpace function of scPred. Model training and cell-type prediction on the query dataset were performed using the trainModel and scPredict functions, respectively.

#### scANVI

scANVI was implemented for scRNA seq cell-type identification evaluation, following the official documentation (https://docs.scvi-tools.org/). Genes expressed in less than 1% of cells were filtered out, and raw count matrices were converted to AnnData objects. Feature selection was performed using Scanpy Wolf et al (2018), and 2000 highly variable genes were retained using the Seurat v3 method. The setup_anndata function of scVI Lopez et al (2018) was then used to prepare the input data. An scVI model was initialized using scvi.model.SCVI and trained with the train function. The trained scVI model was then used to initialize scANVI using the scvi.model.SCANVI.from_scvi_model function. The scANVI model was trained using the train function with default parameters, and cell types in the query dataset were then predicted using the predict function.

#### scSorterDL

scSorterDL was implemented for scRNA-seq cell type identification evaluation following the official documentation (https://github.com/kellen8hao/scSorterDL). Genes expressed in less than 1% of cells were filtered out, and raw count matrices were normalized to counts per 10,000 (CPM), followed by log1p transformation. An ensemble of 300 Linear Discriminant Analysis (LDA) models was initialized using the LocalSwarmLDA function. The model was fit using model.fit function with the following hyperparameters - ‘ldareg’ = 0.000001, ‘shrinkage’ = True, ‘gene_sampling’ = “uniform” and ‘cell_sampling’ = “uniform”. Each LDA model was augmented with a deep neural network (DNN) and the ensemble of DNNs was trained with the following hyperparameters - ‘epochs’ = 100, ‘batch_size’ = 200 and ‘lr’ = 0.05. Cell types in the query dataset were predicted using the model.pred function.

#### CellTypist

CellTypist was implemented for scRNA seq cell type identification evaluation following the official documentation (https://celltypist.readthedocs.io/). Genes expressed in less than 1% of cells were filtered out, and raw count matrices were converted into AnnData objects. The data was normalized using normalize_total and log1p function of scanpy. The model was trained using the celltypist.train function with the following parameters - ‘feature_selection’ = True, ‘use_SGD’ = False, and ‘n_jobs’ = 10. Cell types in the query dataset were then predicted using the celltypist.annotate function with ‘mode’ = “best match” and ‘majority_voting’ = True.

#### UMINT

UMINT was implemented for single-cell multiome cell type identification, following the official documentation (https://github.com/deeplearner87/UMINT). Genes expressed in less than 1% of cells were filtered out, and the raw count matrices were log-transformed and scaled. The model was trained using the CombinedEncoder function with the following parameters: ‘layer_neuron’ = [128, 10], ‘mid_neuron’ = 100, ‘seed’ = 42, ‘lambda_act’ = 0.0001, ‘lambda_weight’ = 0.001, ‘epoch’ = 25, and ‘bs’ = 16. The encodings were extracted using the predict function. By default, UMINT provides a simple neural network classifier for cell type predictions, similar to the one used by Hydra. The neural network classifier was trained on the embeddings with the following parameters: ‘layer_neuron’ = [900, 1500], ‘loss’ = “categorical_crossentropy,” ‘epoch’ = 25, and ‘bs’ = 8.

#### MOFA+

MOFA+ was implemented for single-cell multiome cell type identification, following the official documentation (https://biofam.github.io/MOFA2/tutorials.html). Genes expressed in less than 1% of cells were filtered out, and the raw count matrices were log-transformed and scaled. The model options were set using the set_model_options function with the following parameters: ‘factors’ = 10, ‘spikeslab_weights’ = True, ‘ard_weights’ = True, and ‘ard_factors’ = True. The training options were set using the set_train_options function with parameters - ‘iter’ = 100, ‘convergence_mode’ = “fast”, ‘dropR2’ = 0.001, and ‘seed’ = 42. The model was then built and run using the build and run functions, respectively. The embeddings obtained were used to train a simple neural network classifier for cell type prediction on the query dataset with the following parameters: ‘loss’ = “categorical_crossentropy,” epoch = 25, and ‘bs’ = 8.

#### scMoMat

scMoMaT was implemented for single-cell multiome cell type identification, following the official documentation (https://github.com/PeterZZQ/scMoMaT). Genes expressed in less than 1% of cells were filtered out, and the raw count matrices were preprocessed using the quantile_norm and quantile_norm_log functions of scMoMaT. The dataset objects were prepared according to the authors’ pipeline. The model was trained using the scmomat_model and train_func functions with the following parameters: ‘K’ = 30 and ‘T’ = 4000. The embeddings were extracted using the extract_cell_factors function. These embeddings were used to train a simple neural network classifier for cell type prediction on the query dataset with the following parameters: ‘loss’ = “categorical_crossentropy,” ‘epoch’ = 25, and ‘bs’ = 8.

#### scGLUE

scGLUE was implemented for single-cell multiome cell type identification following the official documentation (https://scglue.readthedocs.io/en/latest). Genes expressed in less than 1% of cells were filtered out, and raw count matrices were converted into AnnData objects. For each modality, preprocessing included normalization to total counts, log1p transformation, selection of 2000 highly variable genes independently using the Seurat method, scaling, and PCA with 100 components. A guidance graph was constructed containing all features from available modalities as vertices with self-loop edges, and then filtered to a subgraph of previously selected features. The model was trained using the scglue.models.fit_SCGLUE function. Cell embeddings were extracted using the encode_data function. Cell types in the query dataset were then predicted using the scglue.data.transfer_labels function.

#### scJoint

scJoint was implemented for single-cell multiome cell type identification following the official documentation (https://github.com/SydneyBioX/scJoint). Genes expressed in less than 1% of cells were filtered out. Raw count matrices were binarized (0 or 1) by setting all values greater than zero to 1. scJoint model was trained with the following parameters: ‘batch_size’ = 256, ‘epochs’ = 20, ‘lr’ = 0.01, ‘momentum’ = 0.9, ‘p’ = 0.8, ‘center_weight’ = 1 and ‘lr_decay_epoch’ = 10. Label transfer was performed following the authors’ implementation of k-nearest neighbors classification (KNN) with ‘k’ = 30 on normalized embeddings. Final cell-type predictions on the query dataset were obtained using KNN with ‘k’ = 30 on concatenated normalized embeddings from all modalities.

#### totalVI

totalVI was implemented for single-cell multiome cell type identification evaluation, following the official documentation (https://docs.scvi-tools.org/). Genes expressed in less than 1% of cells were filtered out, and raw count matrices were converted to AnnData objects. Scanpy was used to select 4000 highly variable genes for the transcriptomic data using the Seurat v3 method. The scvi.model.TOTALVI.setup_anndata function of scVI was used to prepare the input data. A totalVI model was initialized using scvi.model.TOTALVI and trained with the train function with default parameters. The trained model was then used to extract cell embeddings using the get_latent_representation function. Following the authors’ implementation, a random forest classifier was trained with ‘class_weight’ = “balanced_subsample” to predict cell types in the query dataset.

#### MultiVI

MultiVI was implemented for single-cell multiome cell type identification evaluation, following the official documentation (https://docs.scvi-tools.org/). Genes expressed in less than 1% of cells were filtered out, and raw count matrices were converted to AnnData objects. Feature selection was performed using Scanpy, and 4000 highly variable genes were selected independently for each modality using the Seurat v3 method. The scvi.model.MULTIVI.setup_mudata function was used to prepare the input data. A MultiVI model was initialized using scvi.model.MULTIVI and the model were trained using the train function with default parameters. The trained model was then used to extract cell embeddings using the get_latent_representation function. Following the authors’ implementation, a random forest classifier was trained with ‘class_weight’ = “balanced_subsample” to predict cell types in the query dataset.

### Benchmarking tasks

#### Feature stability of Hydra model variants

We utilized stratified random sampling based on original cell type proportions to obtain five different subsets from each dataset. Different model variants of Hydra were then applied to each subset to obtain cell-type-specific features. To quantify feature stability within a dataset, we computed the Pearson correlation coefficient of importance scores between every pair of subsets. If *S*_*i*_ and *S*_*j*_ are two different subsets from a given dataset, the Pearson correlation coefficient *r*_*ij*_ was computed as:

$${r}_{ij}=\frac{{\sum }_{k=1}^{n}\left({x}_{k}^{{S}_{i}}-{\overline{x}}^{{S}_{i}}\right)\,\left({x}_{k}^{{S}_{j}}-{\overline{x}}^{{S}_{j}}\right)}{\sqrt{{\sum }_{k=1}^{n}{\left({x}_{k}^{{S}_{i}}-{\overline{x}}^{{S}_{i}}\right)}^{2}}\,\sqrt{{\sum }_{k=1}^{n}{\left({x}_{k}^{{S}_{j}}-{\overline{x}}^{{S}_{j}}\right)}^{2}}}$$

Where ${x}_{k}^{{S}_{i}}$ and ${x}_{k}^{{S}_{j}}$ are the importance scores of the *k*th feature in subsets *S*_*i*_ and *S*_*j*_, ${\overline{x}}^{{S}_{i}}$ and ${\overline{x}}^{{S}_{j}}$ are the mean importance scores in subsets *S*_*i*_ and *S*_*j*_, and *n* is the total number of features evaluated.

#### Feature correlation

We assessed the correlation of top features selected by different feature selection methods using a subsampled dataset with 20 random cell types from the Mouse Cell Atlas. Half of the cell types in the subsampled dataset were considered as major, and the remaining as minor, with a major-to-minor cell imbalance ratio of 100:2. We then applied different feature selection methods to obtain cell type-specific features. The correlation of importance scores was then computed for the top 100 features identified for each cell type. Finally, hierarchical clustering was performed on the correlation matrix to assess similarities in feature importance between cell types.

#### Selecting cell type-specific markers

Similar to feature correlation, we applied feature selection methods on the subsampled dataset from the Mouse Cell Atlas to select markers for the 20 cell types. Next, we randomly sampled 200 cells for each cell type from the original Tabula Muris dataset and examined the expression patterns of the top 100 genes from each cell type. The selection of cell type-specific genes was determined by assessing whether the top genes identified for a cell type of interest exhibited expression specific to that cell type compared to all other cell types.

#### Cell type identification

We divided the cell type identification task into intra-dataset and inter-dataset annotation tasks. For intra-dataset annotation, the dataset was split into train and test sets and evaluated with five-time repeated random subsampling validation, stratified based on original cell type proportions. We then computed the variability in classifying each cell type across different validation subsets. For inter-dataset tasks, cell type identification was evaluated by training on one dataset and testing on an independent dataset (e.g., batch, studies). The methods were applied to simultaneously predict all cell types in the query dataset. The following metrics were used to evaluate cell type identification. All metrics are reported as percentages:

**Recall:** Recall measures the proportion of cells of a given cell type *c* that were correctly identified. It is computed as:

$${{{{\rm{Recall}}}}}_{c}=\frac{{{{{\rm{TP}}}}}_{c}}{{{{{\rm{TP}}}}}_{c}+{{{{\rm{FN}}}}}_{c}}$$

where TP_*c*_ is the number of true positives and FN_*c*_ is the number of false negatives.

**Precision:** Precision measures the fraction of cells assigned as cell type *c* that truly belong to that type. It is computed as:

$${{{{\rm{Precision}}}}}_{c}=\frac{{{{{\rm{TP}}}}}_{c}}{{{{{\rm{TP}}}}}_{c}+{{{{\rm{FP}}}}}_{c}}$$

Where FP_*c*_ is the number of false positives.

**F1-score:** The F1-score represents the harmonic mean of precision and recall. It is computed as:

$${{{{\rm{F1}}}}\mbox{-}{{{\rm{score}}}}}_{c}=\frac{2\times {{{{\rm{Precision}}}}}_{c}\times {{{{\rm{Recall}}}}}_{c}}{{{{{\rm{Precision}}}}}_{c}+{{{{\rm{Recall}}}}}_{c}}$$

**Balanced accuracy:** Balanced accuracy for a given dataset is quantified as the arithmetic mean of recall across all *N* cell types. It is computed as:

$$\,{{{\rm{Balanced\; Accuracy}}}}=\frac{1}{N}{\sum }_{c=1}^{N}{{{{\rm{Recall}}}}}_{c}$$

**Macro precision:** Macro precision for a given dataset is quantified as the arithmetic mean of precision across all cell types. It is computed as:

$$\,{{{\rm{Macro\; Precision}}}}=\frac{1}{N}{\sum }_{c=1}^{N}{{{{\rm{Precision}}}}}_{c}$$

**Macro F1-score:** Macro F1-score for a given dataset is quantified as the arithmetic mean of F1-scores across all cell types. It is computed as:

$$\,{{{\rm{Macro}}}}\; {{{\rm{F1}}}}\mbox{-}{{{\rm{score}}}}=\frac{1}{N}{\sum }_{c=1}^{N}{{{{\rm{F1}}}}\mbox{-}{{{\rm{score}}}}}_{c}$$

### Computational resources analysis

We assessed runtime and GPU memory consumption across varying dataset sizes to evaluate the scalability of different methods. We created subsampled datasets by selecting 250, 500, 1000, 5000, and 10,000 cells, stratified by original cell types proportions from single-cell transcriptomic data (lung) (Madissoon et al, 2019), joint profiling of single-cell transcriptome and chromatin accessibility (skin) (Ma et al, 2020), and joint profiling of single-cell transcriptome and surface proteins (PBMC) (Stephenson et al, 2021). The number of cell types was kept constant across all subsets to control for cell type variability. For each dataset size, we measured the total runtime and peak GPU memory consumption required for training the model, including classifiers and feature selection. All analyses were performed on a high-performance computing cluster equipped with NVIDIA H100 80GB HBM3 GPU and Intel Xeon Platinum 8462Y+ processors with 32 CPU cores. We used GPU-training for methods that supported it. Runtime and GPU memory consumption were sampled every 2 seconds during execution.

## Supplementary information

Peer Review File

Appendix

Source data Fig. 2

Source data Fig. 3

Source data Fig. 4

Source data Fig. 5

Source data Fig. 6

## Data Availability

The computer code produced in this study is available in the following databases: GitHub (https://github.com/SydneyBioX/Hydra). Documentation for using the tool is available at (https://sydneybiox.github.io/Hydra/). The source data of this paper are collected in the following database record: biostudies:S-SCDT-10_1038-S44320-026-00208-7.
