# Supplementary material for: Interpretable deep generative ensemble learning for single-cell omics with Hydra
Source: Mol Syst Biol. 2026 Apr 11;22(7):1161–79. doi: 10.1038/s44320-026-00208-7 (PMC13328726; doi:10.1038/s44320-026-00208-7)
Supplement: Supplementary file 2 — Appendix [file 44320_2026_208_MOESM2_ESM.pdf]

Appendix for Interpretable deep generative ensemble learning for single-cell omics with Hydra

Table of Contents

Appendix Figure S1. Feature stability of Hydra model variants in lung datasets 1

Appendix Figure S2. Evaluation of different attribution approaches in Hydra 2

Appendix Figure S3. Hierarchical clustering of cell types using statistical feature selection 3

Appendix Figure S4. Gene Ontology enrichment analysis of Hydra-selected features 4

Appendix Figure S5. Intra-dataset prediction performance in Prostate Urethra scRNA-seq 5

Appendix Figure S6. Intra-dataset prediction performance in Colon scRNA-seq 6

Appendix Figure S7. Inter-dataset prediction performance in scRNA-seq datasets 7

Appendix Figure S8. Intra-dataset prediction performance in Skin SHARE-seq 8

Appendix Figure S9. Intra-dataset prediction performance in Brain SNARE-seq 9

Appendix Figure S10. Intra-dataset prediction performance in Kidney sciCAR-seq 10

Appendix Figure S11. Intra-dataset prediction performance in PBMC CITE-seq 11

Appendix Figure S12. Inter-dataset prediction performance in single-cell multiome datasets 12

Appendix Figure S13. Mapping cellular subtypes in healthy MFC brain multiome data 13

Appendix Figure S14. Cross-condition mapping of MFC cellular subtypes in Alzheimer’s disease 14

Appendix Table S1. Summary of all datasets used in the current study 15

Appendix Table S2. Summary of default Hydra hyperparameters 17

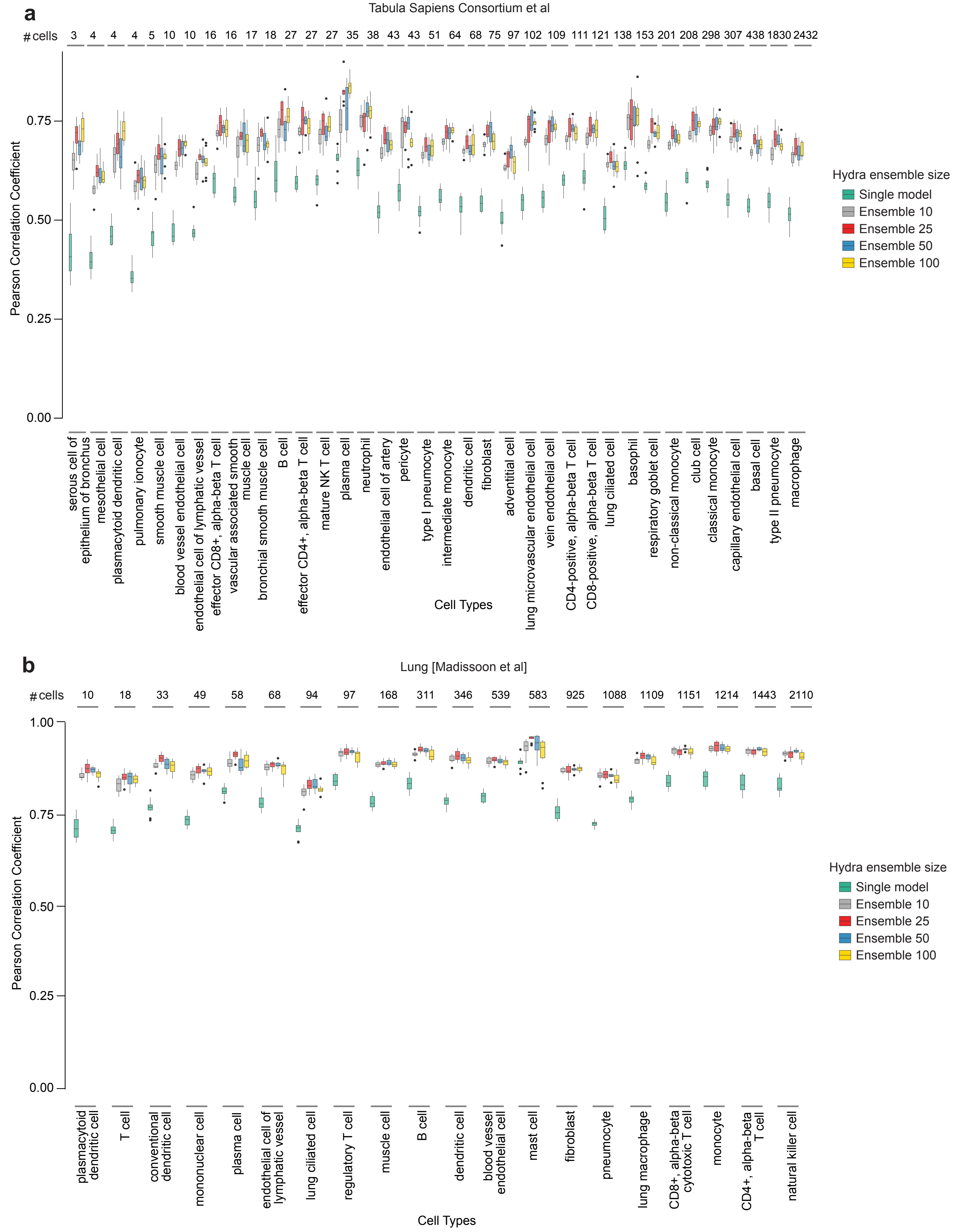

Appendix Figure S1. Feature stability of Hydra model variants (a) Stability of features selected by different model variants of Hydra (n = 1, 10, 25, 50, 100) showing all cell types using the lung dataset (36 cell types), quantified by Pearson correlation coefficients. Cell types are arranged in the order of increasing sample count from left to right. (b) Stability of features selected by different model variants of Hydra (n = 1, 10, 25, 50, 100) showing all cell types using the lung dataset (20 cell types), quantified by Pearson correlation coefficients. Cell types are arranged in the order of increasing sample count from left to right.

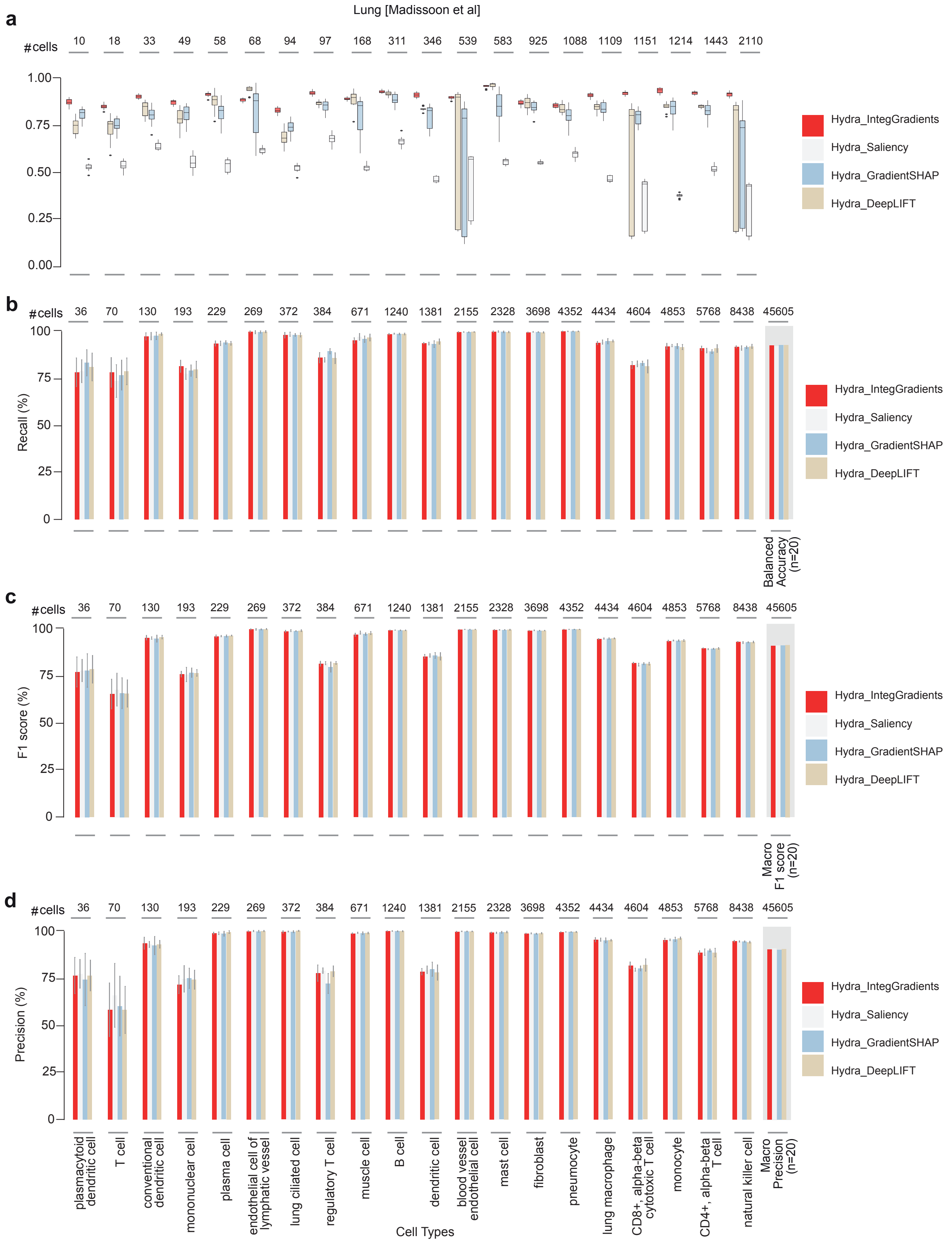

Appendix Figure S2. Evaluation of different attribution approaches of Hydra (a) Stability of features selected by different attribution approaches in Hydra (Integrated Gradients, Saliency, GradientSHAP, and DeepLIFT) for all cell types in the lung dataset (n=20), quantified by Pearson correlation coefficients. Cell types are arranged in the order of increasing sample count from left to right. (b-d) Cell type prediction performance of different attribution approaches in Hydra evaluated using recall (b), F1 score (c) and precision (d). Cell types are arranged in the order of increasing sample count from left to right, followed by the overall performance (highlighted with a gray background).

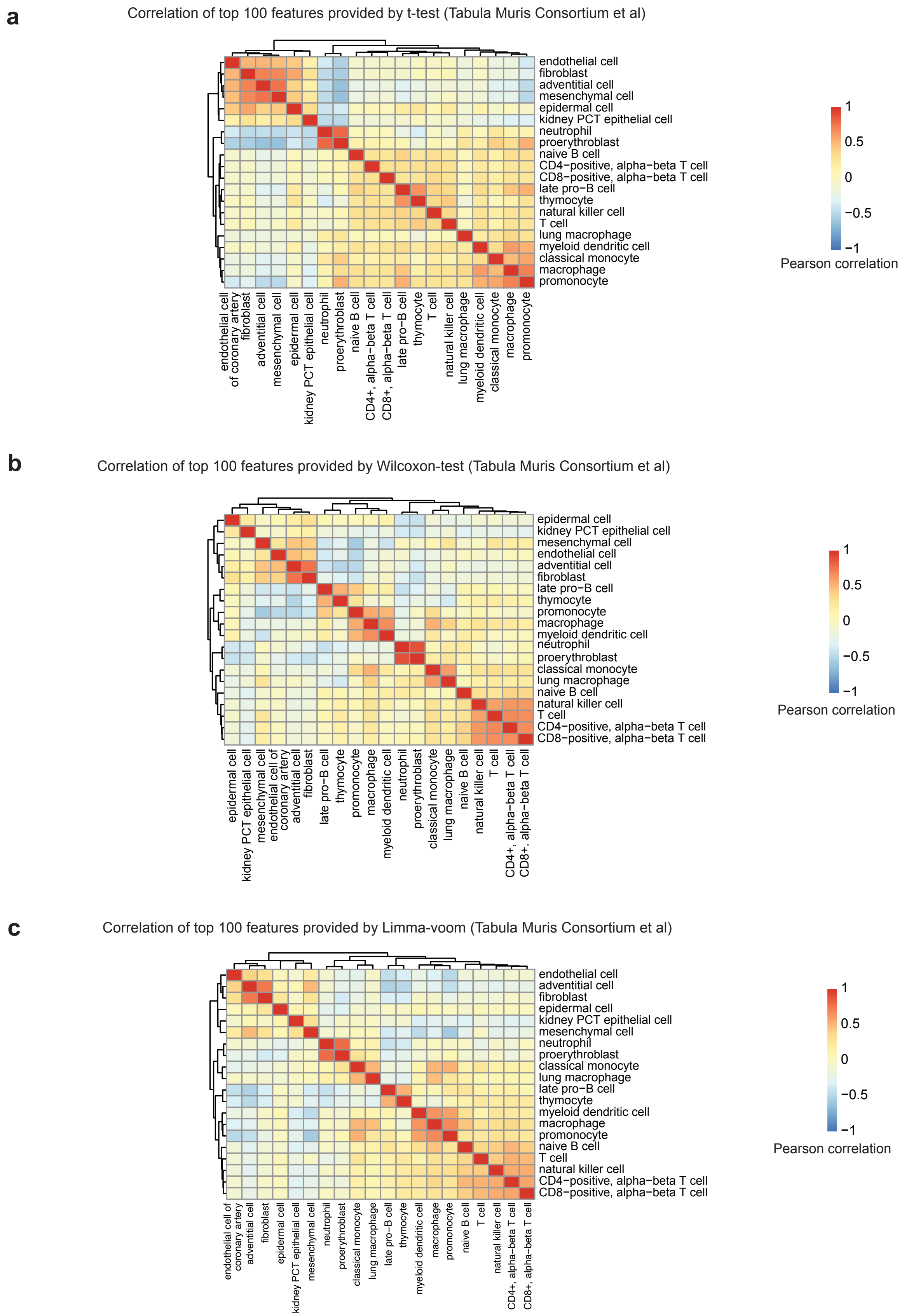

Appendix Figure S3. Hierarchical clustering of cell types using the subsampled dataset based on top features selected by statistical methods Hierarchical clustering of twenty cell types using the top 100 features selected by the t-test (a), Wilcoxon-test (b), and Limma-voom (c) on the subsampled Mouse Cell Atlas. The dataset comprises ten major cell types and ten minor cell types, with an imbalance ratio of major to minor cells of 100:2.

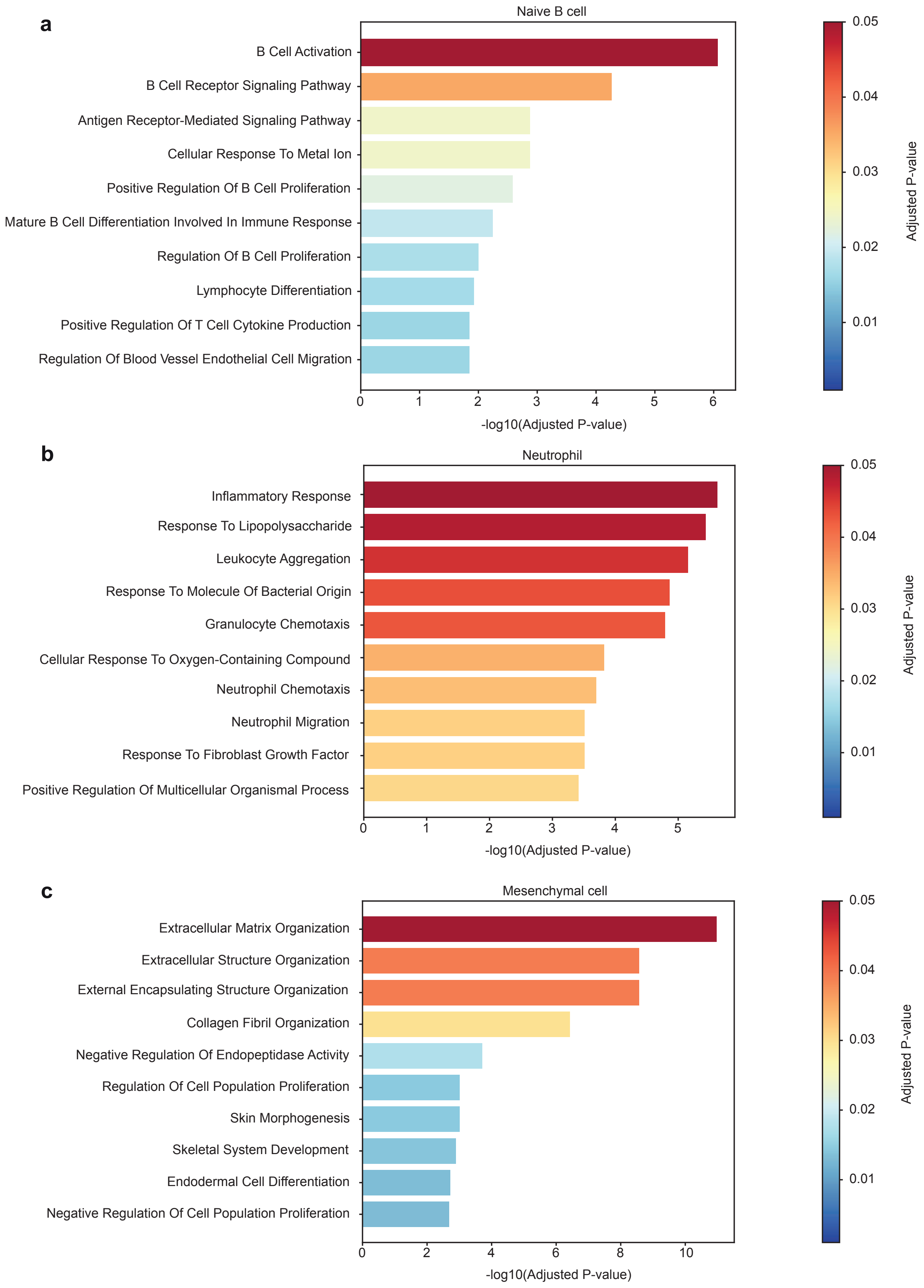

Appendix Figure S4. Gene Ontology enrichment analysis of features selected by Hydra Horizontal bar plots showing the top 10 significantly enriched terms (adjusted p-value < 0.05) for naive B cells (a), neutrophils (b), and mesenchymal cells (c). Enrichment analysis was performed using the top 100 features selected by Hydra for each cell type. Bar length represents -log10(adjusted p-value), and color intensity represents the significance level of enrichment.

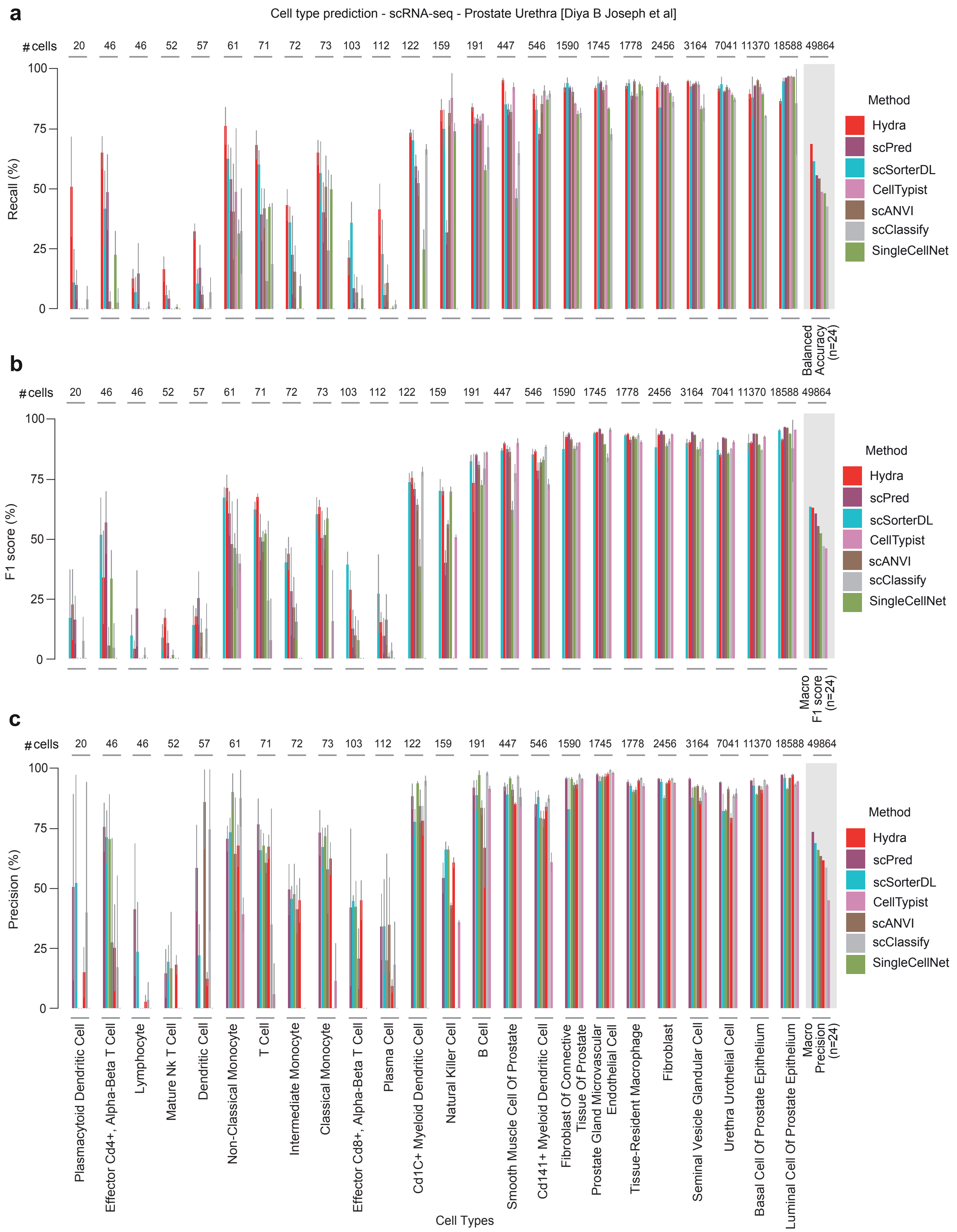

Appendix Figure S5. Intra-dataset cell type prediction performance of all cell types in Prostate Urethra scRNA-seq dataset (a-c) Bar plot with error bars illustrating the five-time repeated random subsampling intra-dataset prediction performance evaluated using recall (a), F1 score (b) and precision(c) for all cell types using the scRNA-seq Prostate Urethra dataset [n=49k cells, 24 cell types]. Cell types are arranged in the order of increasing sample count from left to right, followed by the overall performance (highlighted with a gray background).

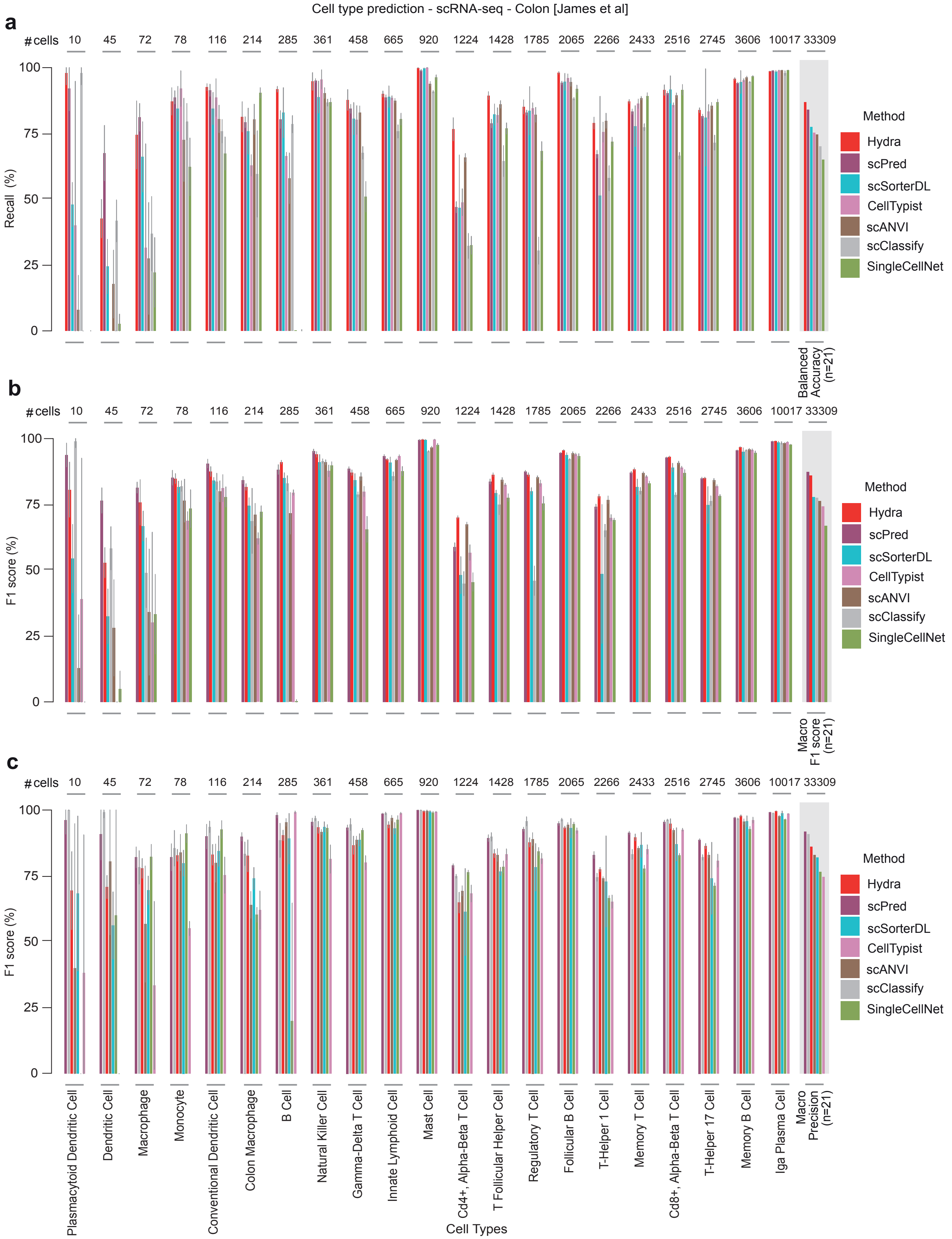

Appendix Figure S6. Intra-dataset cell type prediction performance of all cell types in Colon scRNA-seq dataset (a-c) Bar plot with error bars illustrating the five-time repeated random subsampling intra-dataset prediction performance evaluated using recall (a), F1 score (b) and precision(c) for all cell types using the scRNA-seq Colon dataset [n=33k cells, 21 cell types]. Cell types are arranged in the order of increasing sample count from left to right, followed by the overall performance (highlighted with a gray background).

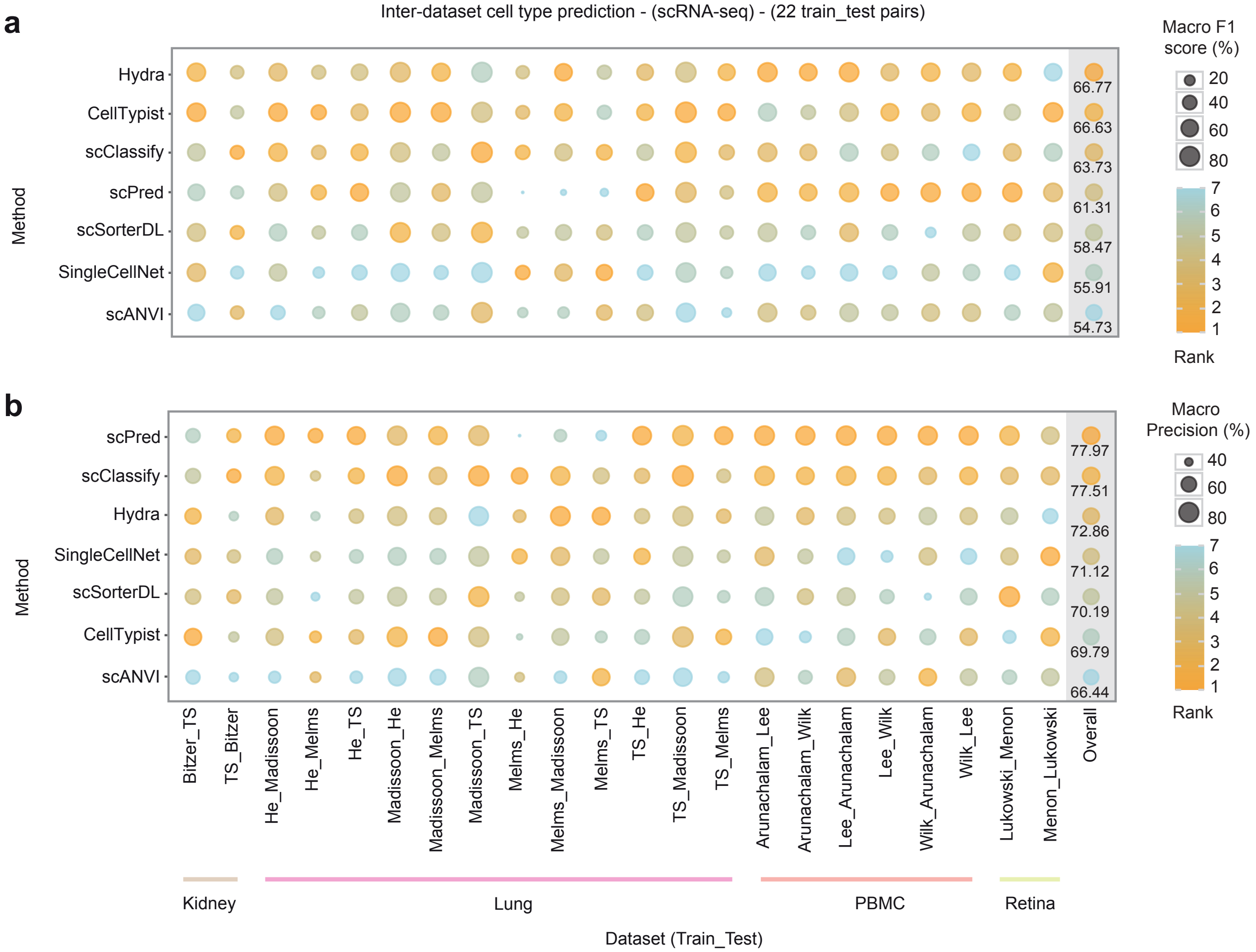

Appendix Figure S7. Inter-dataset cell type prediction performance using single-cell transcriptomic datasets (a-b) Bubble plots illustrating the inter-dataset cell type prediction performance of various methods across four tissues (Kidney, Lung, PBMC, and Retina) and 22 train-test pairs evaluated using the macro F1 score (a) and macro precision (b). Methods are ranked based on their overall performance across all train-test pairs.

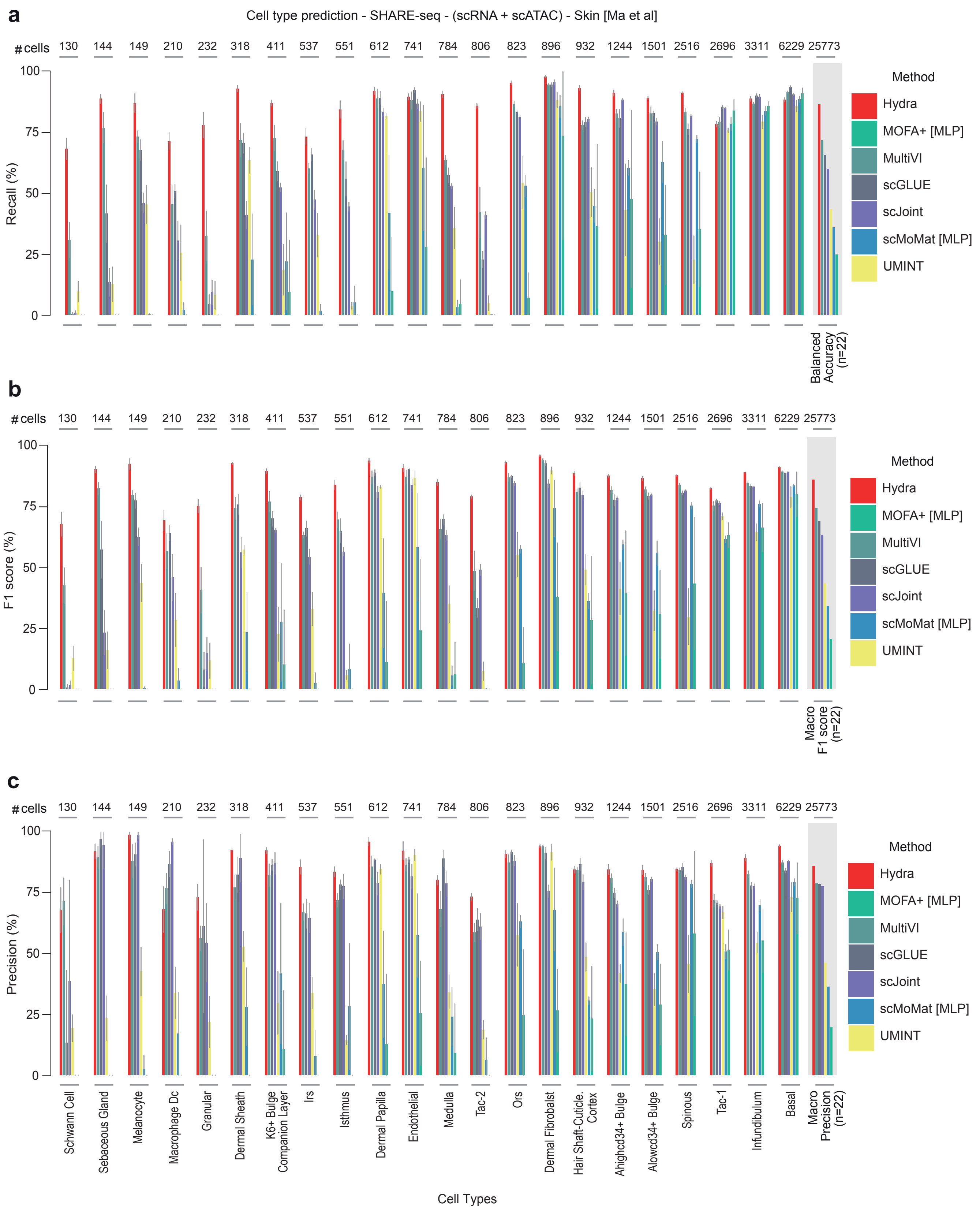

Appendix Figure S8. Intra-dataset cell type prediction performance of all cell types in Skin SHARE-seq dataset (a-c) Bar plot with error bars illustrating the five-time repeated random subsampling intra-dataset prediction performance evaluated using recall (a), F1 score (b) and precision(c) for all cell types using the SHARE-seq (Simultaneous High-throughput ATAC and RNA Expression with sequencing) Skin dataset [n=25k cells, 22 cell types]. Cell types are arranged in the order of increasing sample count from left to right, followed by the overall performance (highlighted with a gray background).

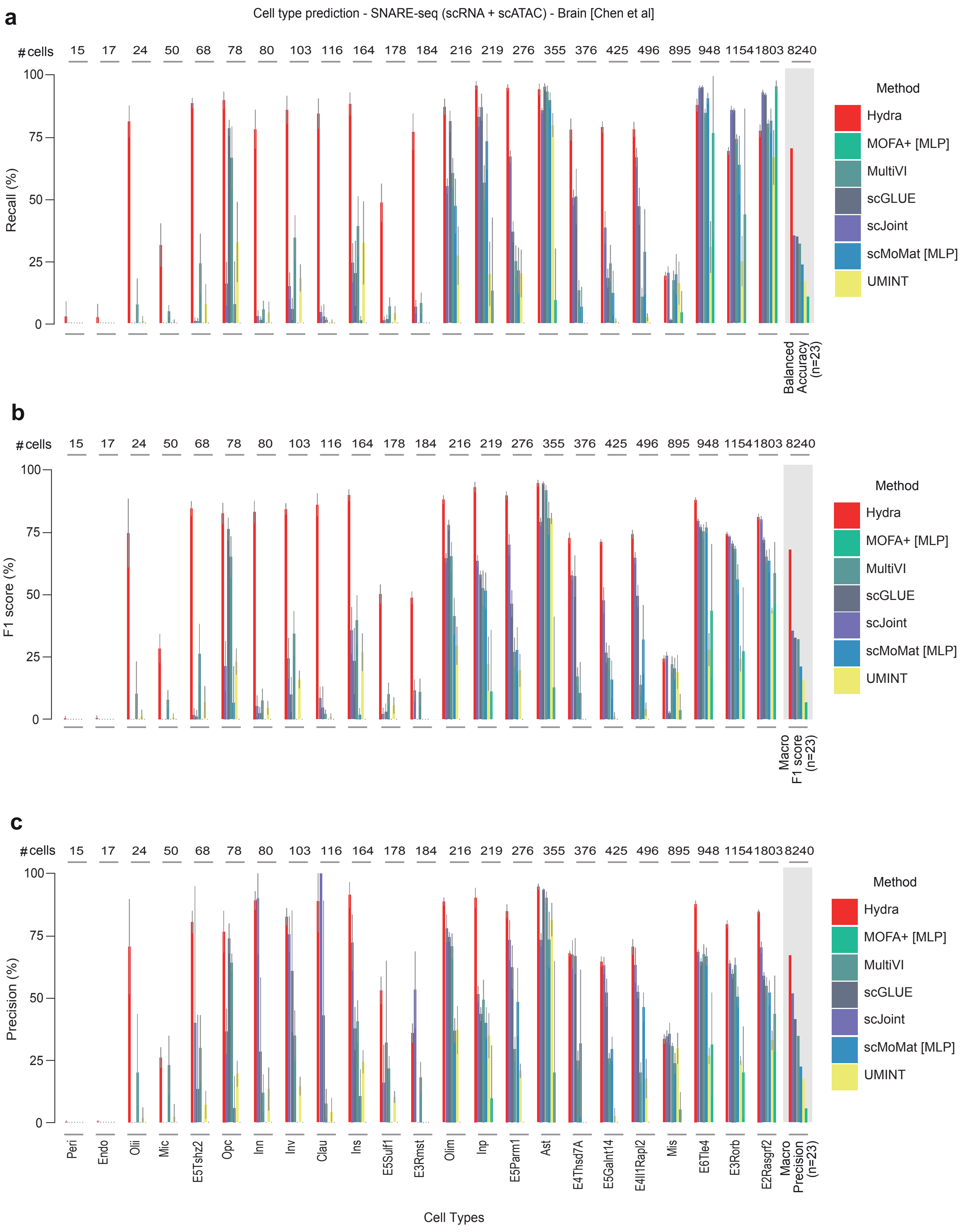

Appendix Figure S9. Intra-dataset cell type prediction performance of all cell types in Brain SNARE-seq dataset (a-c) Bar plot with error bars illustrating the five-time repeated random subsampling intra-dataset prediction performance evaluated using recall (a), F1 score (b) and precision(c) for all cell types using the SNARE-seq (Single-Nucleus Chromatin Accessibility and mRNA Expression sequencing) Brain dataset [n=8k cells, 23 cell types]. Cell types are arranged in the order of increasing sample count from left to right, followed by the overall performance (highlighted with a gray background).

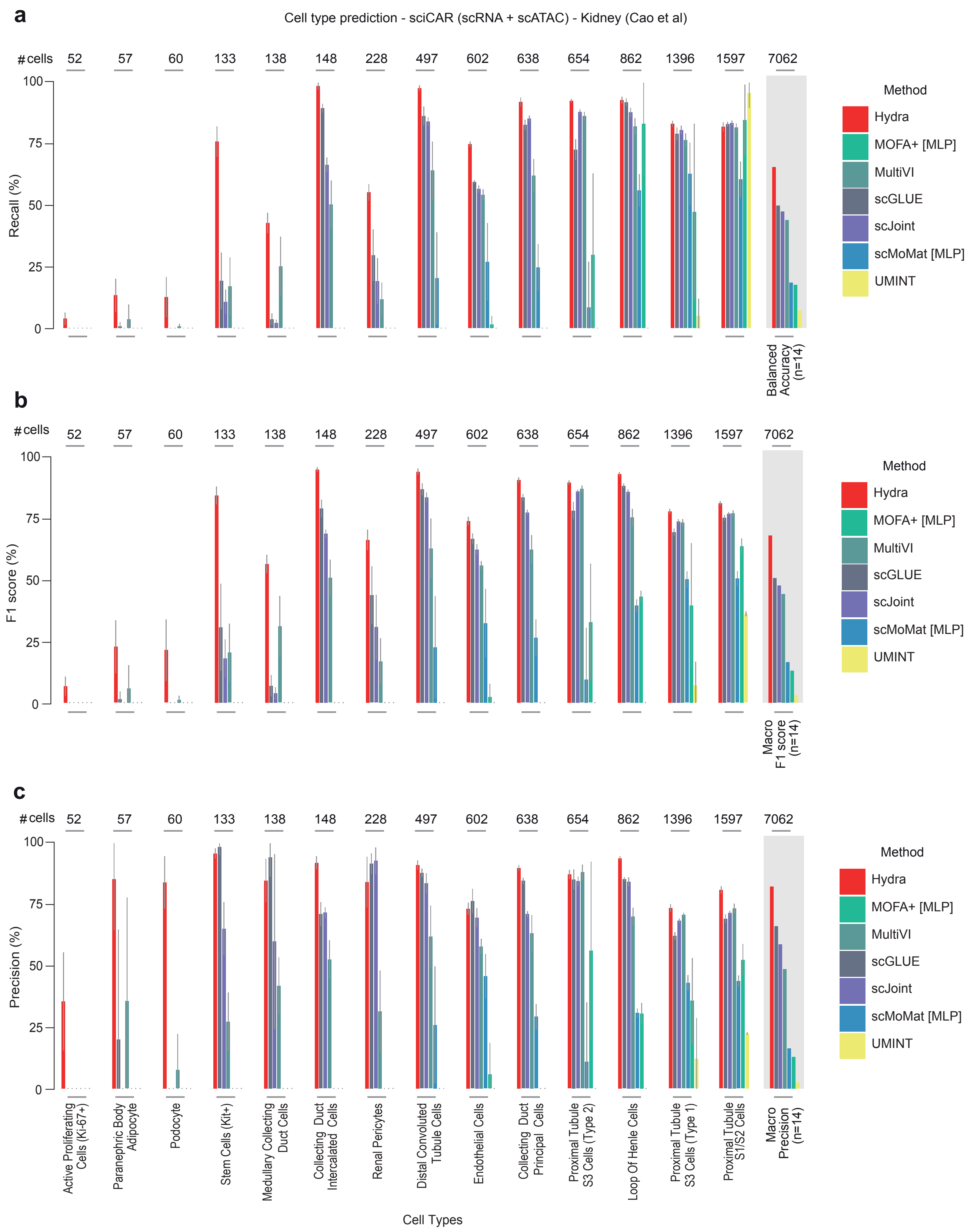

Appendix Figure S10. Intra-dataset cell type prediction performance of all cell types in Kidney sciCAR-seq dataset (a-c) Bar plot with error bars illustrating the five-time repeated random subsampling intra-dataset prediction performance evaluated using recall (a), F1 score (b) and precision(c) for all cell types using the sciCAR-seq (Single-Cell Combinatorial Indexing chromatin accessibility and RNA sequencing) Kidney dataset [n=7k cells, 14 cell types]. Cell types are arranged in the order of increasing sample count from left to right, followed by the overall performance (highlighted with a gray background).

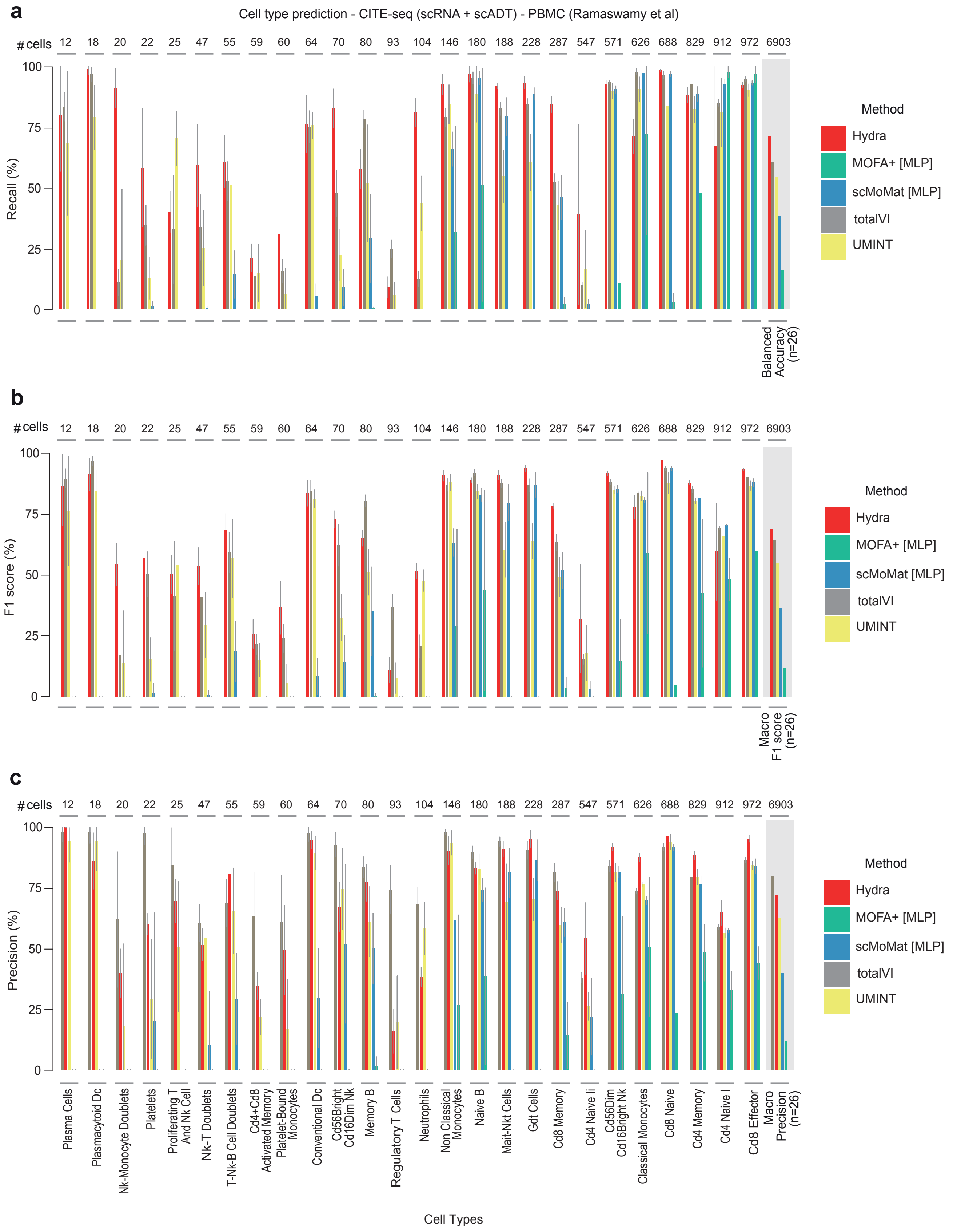

Appendix Figure S11. Intra-dataset cell type prediction performance of all cell types in PBMC CITE-seq dataset (a-c) Bar plot with error bars illustrating the five-time repeated random subsampling intra-dataset prediction performance evaluated using recall (a), F1 score (b) and precision(c) for all cell types using the CITE-seq (Cellular Indexing of Transcriptomes and Epitopes by sequencing) PBMC dataset [n=6k cells, 26 cell types]. Cell types are arranged in the order of increasing sample count from left to right, followed by the overall performance (highlighted with a gray background).

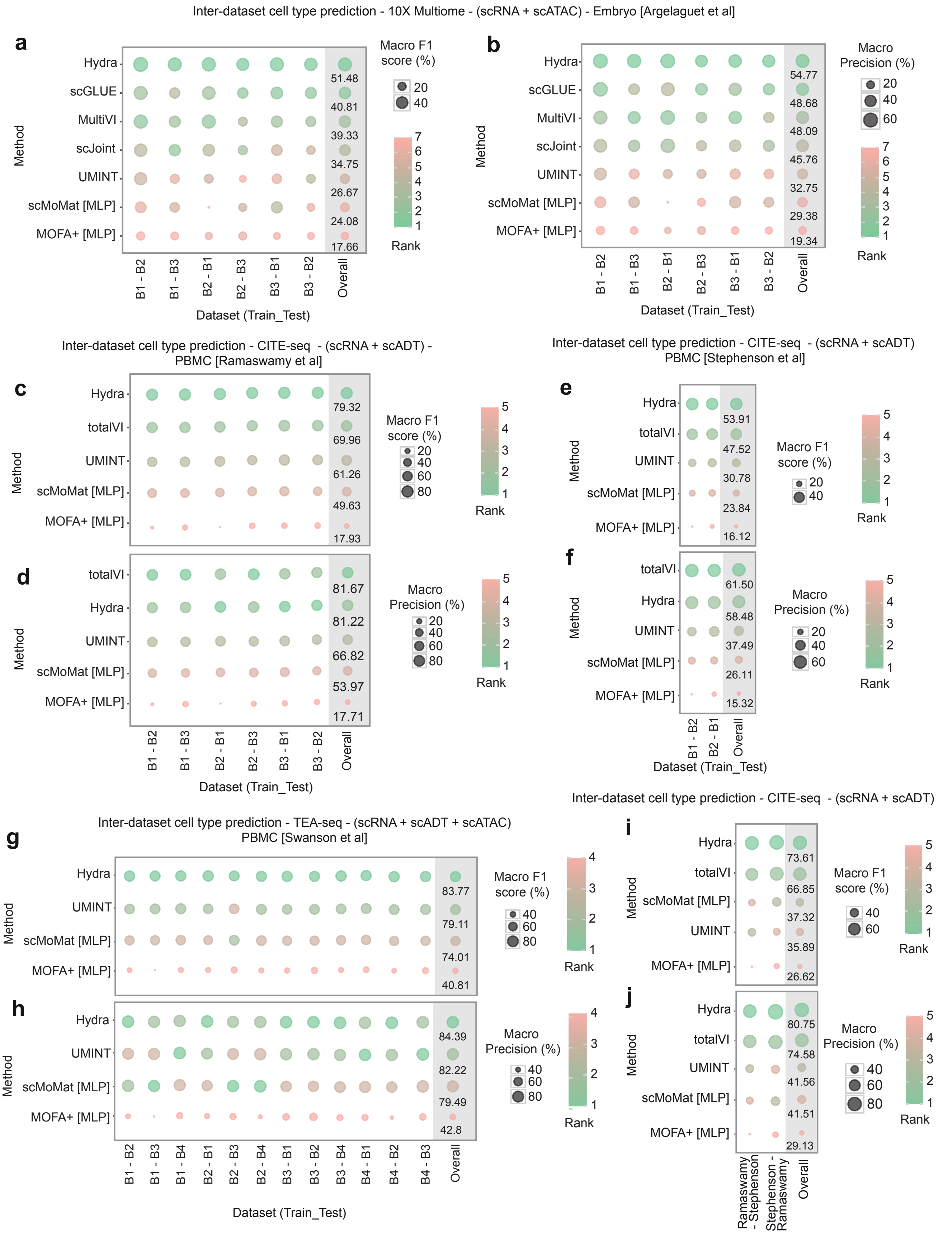

Appendix Figure S12. Inter-dataset cell type prediction performance in single-cell multiome datasets Bubble plots showing inter-dataset cell type prediction performance across various methods, evaluated using macro F1 score and macro precision.(a-b) 10X Multiome Embryo dataset, (c-d) CITE-seq PBMC [Ramaswamy et al], (e-f) CITE-seq PBMC [Stephenson et al], (g-h) TEA-seq PBMC, (i-j) CITE-seq PBMC [Ramaswamy et al; Stephenson et al] datasets. Methods are ranked by overall performance across all train-test splits.

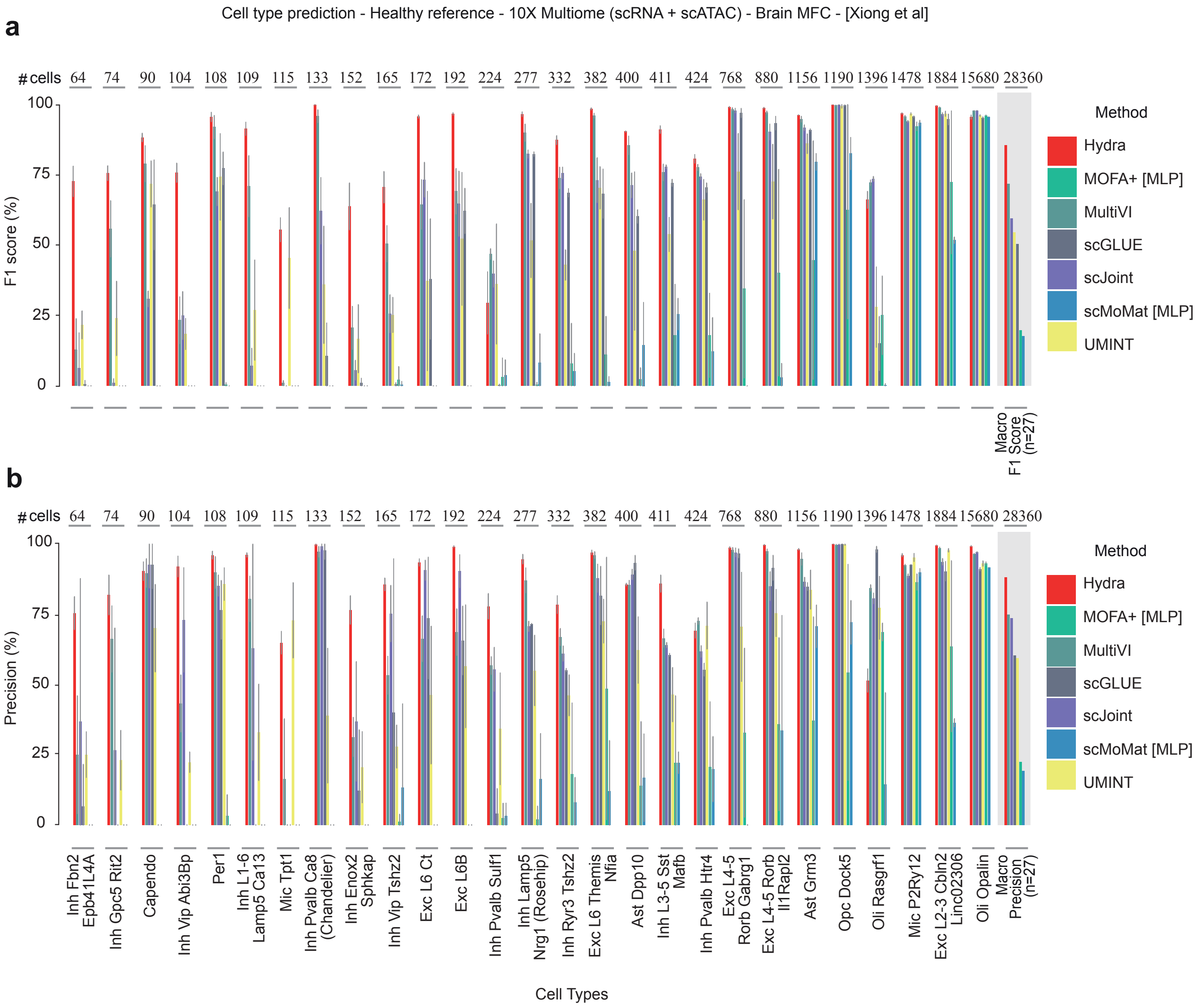

Appendix Figure S13. Mapping cellular subtypes of brain medial frontal cortex (MFC) in healthy reference (a-b) Bar plot with error bars illustrating the 5-fold intra-dataset cell type prediction performance of Hydra against different single-cell multiome methods using healthy medial frontal cortex (MFC) brain dataset. Methods are evaluated using the F1 score (a) and precision (b). Prediction performance is displayed for all cell types, followed by the overall performance across all cell types (highlighted with a gray background). Cell types are arranged in the order of increasing sample count from left to right.

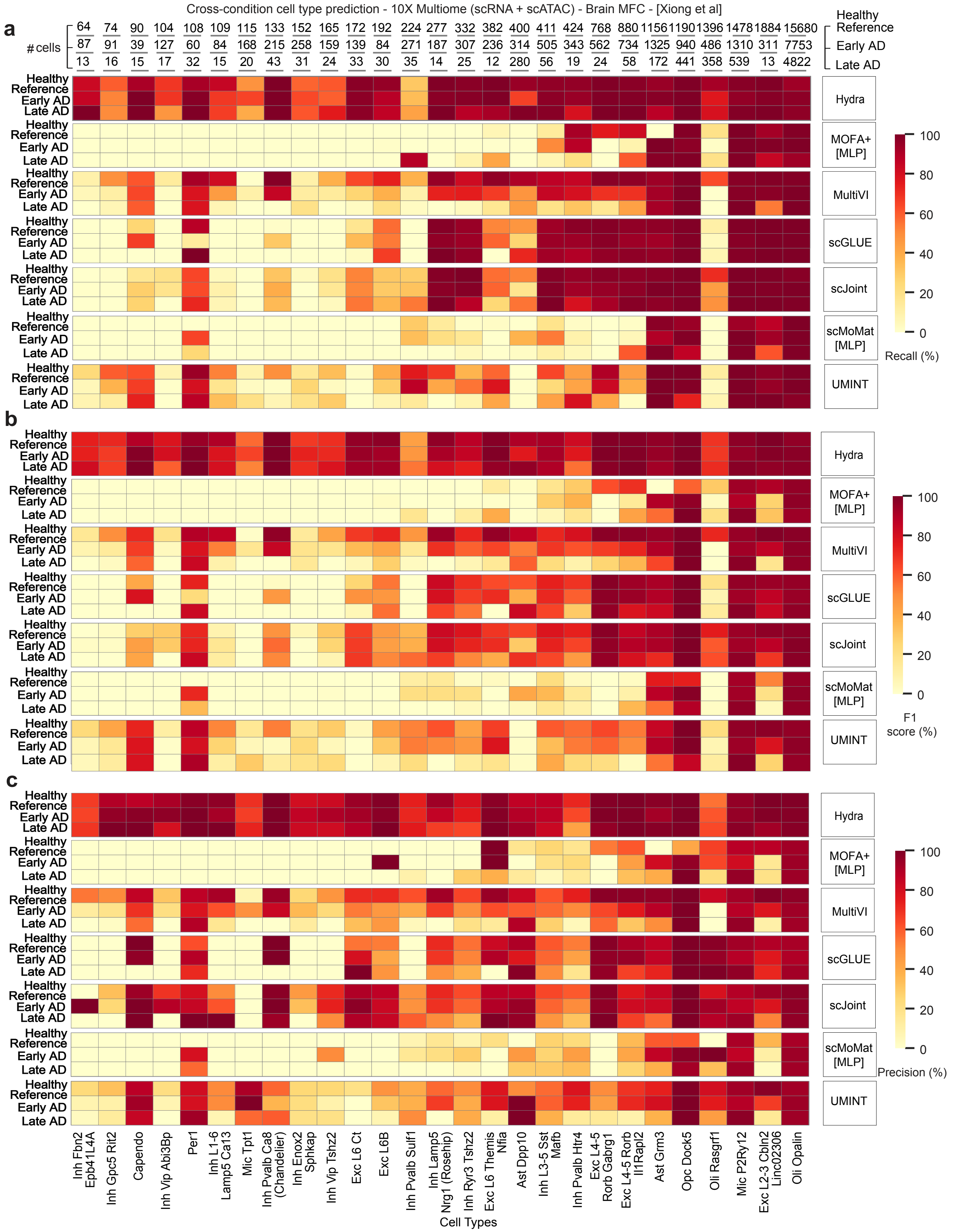

Appendix Figure S14. Cross-condition mapping of brain MFC cellular subtypes in Alzheimer's disease (a-c) Heatmaps showing cross-condition cell type prediction performance of different single-cell multiome methods using models trained on healthy reference dataset. Performance is evaluated using recall (a), F1 score (b) and precision (c). Cell types are arranged in the order of increasing sample count from left to right based on healthy reference.

No.	Dataset	Modality	Batch	Tissue	Technology	Species	No. of cell types	No. of cells
1	(Madissoon et al.)	RNA	B1	Lung	10x 3' v2	Human	20	57,019
2	(Tabula Sapiens Consortium* et al.)	RNA	B1	Lung	10x 3' v3 Smart-seq2	Human	37	35,682
		RNA	B1	Kidney	10x 3' v3 Smart-seq2		7	9,641
3	(The Tabula Muris Consortium et al.)	RNA	B1	Twenty different tissues	10x 3' v2	Mouse	30	24,540
4	(Diya B Joseph et al.)	RNA	B1	Prostate Urethra	10x 3' v2 10x 3' v3	Mouse	26	62,415
5	(James et al.)	RNA	B1	Colon Caecum Lymph node	10x 3' v2 10x 5' v2	Human	21	41,650
6	(Bitzer et al.)	RNA	B1	Kidney	10x 3' v3	Human	28	48,783
7	(He et al.)	RNA	B1	Lung	10x 5' v1	Human	77	71,752
8	(Melms et al.)	RNA	B1	Lung	10x 3' v3	Human	30	36,677
9	(Arunachalam et al.)	RNA	B1	PBMC	10x 3' v3	Human	25	25,954
10	(Wilk et al.)	RNA	B1	PBMC	Seq-Well	Human	26	15,765
11	(Lee et al.)	RNA	B1	PBMC	10x 3' v3	Human	24	16,298
12	(Menon et al.)	RNA	B1	Retina	10x 3' v3	Human	9	20,091
13	(Lukowski et al.)	RNA	B1	Retina	10x 3' v2	Human	9	17,884

Appendix Table S1. Summary of all datasets used in the current study

No.	Dataset	Modality	Batch	Tissue	Technology	Species	No. of cell types	No. of cells
14	(Ma et al.)	RNA ATAC	B1	Skin	SHARE-seq	Mouse	23	34,774
15	(Chen et al.)	RNA ATAC	B1	Brain	SNARE-seq	Mouse	23	10,309
16	(Cao et al.)	RNA ATAC	B1	Kidney	sci-CAR	Mouse	14	11
17	(Argelaguet, Lohoff, et al.)	RNA ATAC	B1	Embryo	10x Multiome	Mouse	32	8,343
			B2				34	10,968
			B3				24	3,977
18	(Xiong et al.)	RNA ATAC	B1 (Healthy reference)	Brain	10x Multiome	Human	27	35,462
			B1 (Early AD				27	17,095
			B1 (Late AD)				27	7,157
19	(Ramaswamy et al.)	RNA ADT	B1	PBMC	CITE-seq	Human	30	8,650
			B2				29	9,538
			B3				30	10,423
20	(Stephenson et al.)	RNA ADT	B1	PBMC	CITE-seq	Human	45	30,313
			B2				48	64,262
21	(Swanson et al.)	RNA ADT ATAC	B1	PBMC	TEA-seq	Human	13	6,314
			B2				12	6,548
			B3				13	6,547
			B4				12	6,756

Appendix Table S1. Summary of all datasets used in the current study

Parameter	Default value
Feature Ranking Module (VAE)	
Learning rate	0.02
Batch size	512
Epochs (Initial training)	40
Epochs (Model refinement)	30-50
Epochs (Early stopping)	10
Ensemble size	25
Latent dimension (z)	100
Hidden layer (RNA)	185
Hidden layer (ADT)	30
Hidden layer (ATAC)	185
Cell Type Annotation Module	
Learning rate	0.01
Batch size	64
Epochs	5
Hidden layer dimension	128
Ensemble size	25

Appendix Table S2. Summary of default hyperparameters for Hydra
