# Supplementary material for: Interpretable deep generative ensemble learning for single-cell omics with Hydra
Source: Mol Syst Biol. 2026 Apr 11;22(7):1161–79. doi: 10.1038/s44320-026-00208-7 (PMC13328726; doi:10.1038/s44320-026-00208-7)
Supplement: Supplementary file 1 — Peer Review File [file 44320_2026_208_MOESM1_ESM.pdf]

Interpretable deep generative ensemble learning for single-cell omics with Hydra

Manoj M Wagle, Chunlei Liu, Zunpeng Liu, Yongheng Wang, Manolis Kellis, Ellis Patrick, and Pengyi Yang

Corresponding author(s): Pengyi Yang (pengyi.yang@sydney.edu.au) , Ellis Patrick (ellis.patrick@sydney.edu.au)

Review Timeline:

Submission Date:	21st Sep 25
Editorial Decision:	25th Oct 25
Revision Received:	3rd Feb 26
Editorial Decision:	6th Mar 26
Revision Received:	13th Mar 26
Accepted:	24th Mar 26

Editor: Poonam Bheda

Transaction Report:

(Note: With the exception of the correction of typographical or spelling errors that could be a source of ambiguity, letters and reports are not edited. Depending on transfer agreements, referee reports obtained elsewhere may or may not be included in this compilation. Referee reports are anonymous unless the Referee chooses to sign their reports.)

25th Oct 2025

Manuscript Number: MSB-2025-13327-T
Title: Interpretable deep generative ensemble learning for single-cell omics with Hydra

Dear Prof Yang,

Thank you again for submitting your work to Molecular Systems Biology. We have now heard back from the three reviewers who agreed to evaluate your study. As you will see below, the reviewers find that the Hydra method is of potential interest. However, they raise a series of concerns, which we would ask you to address in a major revision.

Without repeating all the comments listed below, some of the more fundamental issues raised are the following:
- benchmarking needs to be improved with comparisons to more recently published, state-of-the-art methods, including a fairer comparison using appropriate parameters (as mentioned by all 3 reviewers)
- computational resources required and scalability of Hydra should be described (as mentioned by Reviewers 2 and 3)
- choice of integrated gradients for feature selection should be explained/justified (as mentioned by Reviewers 1 and 3)

All other issues raised would need to be satisfactorily addressed. Please let me know in case you would like to discuss in further detail any of the comments, I would be happy to schedule a call.

When submitting your revised manuscript, please carefully review the instructions that follow below. We perform an initial quality control of all revised manuscripts before re-review; failure to include requested items will delay the evaluation of your revision.

We require:

1) A .docx formatted version of the manuscript text (including legends for main figures, EV figures and tables). Please make sure that the changes are highlighted to be clearly visible. Alternatively you may choose to submit your manuscript as a LaTeX file.

2) Individual production quality figure files as .eps, .tif, .jpg (one file per figure). For guidance, download the 'Figure Guide PDF' (https://www.embopress.org/page/journal/17574684/authorguide#figureformat).

3) At EMBO Press we ask authors to provide source data for the main figures. Our source data coordinator will contact you to discuss which figure panels we would need source data for and will also provide you with helpful tips on how to upload and organize the files.

4) A .docx formatted letter INCLUDING the reviewers' reports and your detailed point-by-point responses to their comments. As part of the EMBO Press transparent editorial process, the point-by-point response is part of the Peer Review File (PRF), which will be published alongside your paper.

5) A complete author checklist, which you can download from our author guidelines (https://www.embopress.org/page/journal/17574684/authorguide#submissionofrevisions). Please insert information in the checklist that is also reflected in the manuscript. The completed author checklist will also be part of the PRF.

6) Please note that all corresponding authors are required to supply an ORCID ID for their name upon submission of a revised manuscript.

7) It is mandatory to include a 'Data Availability' section after the Materials and Methods. Before submitting your revision, primary datasets produced in this study need to be deposited in an appropriate public database, and the accession numbers and database listed under 'Data Availability'. Please remember to provide a reviewer password if the datasets are not yet public (see https://www.embopress.org/page/journal/17574684/authorguide#dataavailability).

In case you have no data that requires deposition in a public database, please state so in this section as follows: "This study includes no data deposited in external repositories". Note that the Data Availability Section is restricted to new primary data that are part of this study.

8) All Materials and Methods need to be described in the main text using our 'Structured Methods' format, which is required for all research articles. According to this format, the Methods section includes a Reagents and Tools Table (listing key reagents, experimental models, software and relevant equipment and including their sources and relevant identifiers) followed by a Methods and Protocols section describing the methods using a step-by-step protocol format. The aim is to facilitate adoption of the methodologies across labs. Please upload the Reagents and Tools table as a separate document when submitting your revised manuscript. More information on how to adhere to this format as well as a downloadable template (.docx) for the

Reagents and Tools Table can be found in our author guidelines:
https://www.embopress.org/page/journal/17444292/authorguide#structuredmethods

An example of a Method paper with Structured Methods can be found here:
https://www.embopress.org/doi/10.15252/msb.20178071.

9) For data quantification: please specify the name of the statistical test used to generate error bars and p-values, the number (n) of independent experiments (specify technical or biological replicates) underlying each data point and the test used to calculate p-values in each figure legend. The figure legends should contain a basic description of n, p-values and the test applied. Graphs must include a description of the bars and the error bars (s.d., s.e.m.). Please provide exact p-values (in either the figure or figure legend).

10) Our journal encourages inclusion of *data citations in the reference list* to directly cite datasets that were re-used and obtained from public databases. Data citations in the article text are distinct from normal bibliographical citations and should directly link to the database records from which the data can be accessed. In the main text, data citations are formatted as follows: "Data ref: Smith et al, 2001" or "Data ref: NCBI Sequence Read Archive PRJNA342805, 2017". In the Reference list, data citations must be labeled with "[DATASET]". A data reference must provide the database name, accession number/identifiers and a resolvable link to the landing page from which the data can be accessed at the end of the reference. Further instructions are available at .

11) We replaced Supplementary Information with Expanded View (EV) Figures and Tables that are collapsible/expandable online. EV Figures should be cited as 'Figure EV1, Figure EV2" etc... in the text and their respective legends should be included in the main text after the legends of regular figures.
- For the figures that you do NOT wish to display as Expanded View figures, they should be bundled together with their legends in a single PDF file called *Appendix*, which should start with a short Table of Content. Appendix figures should be referred to in the main text as: "Appendix Figure S1, Appendix Figure S2" etc.
- Additional Tables/Datasets should be labeled and referred to as Table EV1, Dataset EV1, etc. Legends should be provided in a separate tab in case of .xls files. Alternatively, the legend can be supplied as a separate text file (README) and zipped together with the Table/Dataset file.
See detailed instructions here:
https://www.embopress.org/page/journal/17574684/authorguide#expandedview

12) Author contributions: CRediT has replaced the traditional author contributions section because it offers a systematic machine-readable author contributions format that allows for more effective research assessment. Please remove the Authors Contributions from the manuscript and use the free text boxes beneath each contributing author's name in our system to add specific details on the author's contribution. More information is available in our guide to authors.

13) Disclosure statement and competing interests: We updated our journal's competing interests policy in January 2022 and request authors to consider both actual and perceived competing interests. Please review the policy https://www.embopress.org/competing-interests and update your competing interests if necessary.

14) Every published paper now includes a 'Synopsis' to further enhance discoverability. Synopses are displayed on the journal webpage and are freely accessible to all readers. They include a short stand first (maximum of 300 characters, including space) as well as 2-5 one-sentences bullet points that summarizes the paper. Please write the bullet points to summarize the key NEW findings. They should be designed to be complementary to the abstract - i.e. not repeat the same text. We encourage inclusion of key acronyms and quantitative information (maximum of 30 words / bullet point). Please use the passive voice. Please attach these in a separate file or send them by email, we will incorporate them accordingly.

Please also suggest a striking image or visual abstract to illustrate your article as a PNG file 550 px wide x 300-600 px high.

Please note that these would be the final versions and changes during proofing are usually not allowed.

15) As part of the EMBO Publications transparent editorial process initiative (see our policy here: https://www.embopress.org/transparent-process#Review_Process), Molecular Systems Biology will publish online a Peer Review File (PRF) to accompany accepted manuscripts.
In the event of acceptance, this file will be published in conjunction with your paper and will include the anonymous referee reports, your point-by-point response and all pertinent correspondence relating to the manuscript. Let us know whether you agree with the publication of the PRF and as here, if you want to remove or not any figures from it prior to publication.
Please note that the Author checklist will be published at the end of the PRF.

Molecular Systems Biology has a "scooping protection" policy, whereby similar findings that are published by others during review or revision are not a criterion for rejection. Should you decide to submit a revised version, I do ask that you get in touch after three months if you have not completed it, to update us on the status.

Yours sincerely,

Poonam Bheda, PhD
Scientific Editor
Molecular Systems Biology

Reviewer #1:

This manuscript introduces Hydra, a deep generative ensemble framework for cell type annotation and feature discovery in single-cell omics data. The study tackles a timely and important problem-the integration of multiple single-cell modalities and the recovery of rare cell types-and combines several interesting components, including ensemble VAEs and gradient-based feature ranking. The idea is creative and could make a meaningful contribution, especially in the rapidly evolving area of multi-omic data analysis.
That said, a number of points need further clarification and support. Some of the central claims, particularly those around interpretability, feature attribution, and the fairness of the benchmarking, are not yet fully convincing. The evaluation metrics are also rather limited given the scope of the conclusions. Overall, this is a promising and well-motivated study, but the work would benefit from stronger methodological justification and a more comprehensive evaluation to fully substantiate its key claims.

Major concerns:
1. The manuscript emphasizes interpretability as one of Hydra's main innovations. However, the framework remains a deep neural network whose internal operations are not inherently interpretable. The claimed interpretability arises only post hoc, through Integrated Gradients-based feature attribution, which generates ranked lists of genes or features associated with each cell type. This output is conceptually similar to standard differential expression (DE) analyses that have long provided interpretable marker sets grounded in explicit statistical models. In this sense, Hydra's interpretability is relative-improved compared to typical black-box neural networks, but not beyond what conventional DE-based approaches already offer. The manuscript would benefit from reframing interpretability as a supporting feature rather than a central methodological innovation.
2. The feature selection component relies exclusively on Integrated Gradients (IG) to identify cell type-specific features, yet no justification is provided for this particular choice. Given the central role of these ranked features in downstream annotation, the decision is non-trivial. The authors should demonstrate why IG is preferred over other attribution methods such as SHAP, DeepLIFT, or simpler gradient saliency techniques. An ablation or comparative analysis showing the effect of these alternatives on feature stability, biological relevance, and annotation accuracy would substantiate the use of IG. Without such evidence, the gradient-based feature selection appears somewhat arbitrary rather than empirically motivated.

3.Although Hydra is benchmarked against multiple existing methods across numerous datasets, the benchmarking design raises concerns about fairness and interpretability. It is unclear whether all baseline models were optimized under comparable conditions; the manuscript notes that default parameters were used for several tools, which may not reflect their optimal performance. Furthermore, the evaluation relies solely on overall accuracy, which can obscure performance differences among rare or ambiguous cell types. Including per-class precision, recall, and F1-scores would provide a clearer picture of the model's strengths and weaknesses. Finally, the computational cost of Hydra's ensemble framework is not reported, leaving scalability and runtime feasibility unaddressed.

4.Hydra's central claim lies in its ability to integrate and annotate multi-omic data. However, the multimodal benchmarking includes only MOFA+, UMINT, and scMoMaT-tools primarily designed for unsupervised embedding or factorization rather than supervised annotation or marker discovery. Several high-impact, state-of-the-art frameworks directly relevant to multi-omic integration and cell type annotation are omitted. These include scGLUE (Nat Biotechnol 2022), scCross (Genome Biol 2024), scJoint (Nat Biotechnol 2022), totalVI / MultiVI (Nat Methods 2020/2023). Benchmarking against these methods would provide a more rigorous and contemporary comparison, allowing Hydra's multi-omic claims to be evaluated in the context of current standards.

5. The evaluation framework relies almost entirely on overall cell type classification accuracy. While this metric is intuitive, it is insufficient to support the breadth of claims made in the manuscript. Overall accuracy tends to be dominated by abundant populations and does not capture performance on rare or underrepresented cell types-one of Hydra's stated strengths. Reporting per-class precision, recall, or F1-scores would yield a more balanced assessment. For cross-dataset evaluations, metrics such as Adjusted Rand Index (ARI) or Normalized Mutual Information (NMI) would better quantify label concordance and generalization. Moreover, given Hydra's emphasis on multi-omic integration, standard integration-quality measures such as LISI, silhouette width, or graph connectivity should be included to assess modality alignment. Finally, for interpretability, a quantitative comparison of Hydra-identified markers against known cell-type gene sets or enrichment results would strengthen biological validation.

6. The manuscript does not clearly describe how the reference cell-type labels were defined across multi-omic datasets. If these annotations originate primarily from the RNA modality-as is typical for multimodal assays such as 10x Multiome or CITE-seq-then the evaluation is inherently biased toward RNA-driven models. This introduces a conceptual problem: models that genuinely integrate additional modalities (e.g., ATAC or ADT) may deviate from RNA-based labels even when capturing more comprehensive biological information. Consequently, better integration of multi-omic signals could paradoxically reduce apparent "annotation accuracy," since the ground-truth labels reflect only one modality. The authors should clarify the provenance of the reference annotations and discuss the potential for such bias. An analysis showing how each modality contributes to Hydra's predictions would help disentangle genuine cross-modal integration from RNA-centric agreement.

Minor comments:
The equations for the variational autoencoder (VAE) objective are structurally correct but inconsistently formatted. The manuscript alternates between x, x(m), and xi to denote input data without clear definition. Each modality (e.g., RNA, ATAC, ADT) should be explicitly specified in the loss formulation, and the relationship between modality-specific encoders and decoders should be clarified for precision.

The loss term LHydra=LVAE+λLCE includes a weighting parameter λ, but its value and tuning procedure are not described. Because this parameter balances reconstruction and classification, even modest changes could alter the results. The manuscript should explain how λ\lambdaλ was chosen-whether by grid search, cross-validation, or fixed heuristics-and whether it was held constant across datasets
.
3.Hydra's ensemble strategy is a central part of the framework, yet the manuscript does not specify how outputs from multiple VAEs are combined. It should be clarified whether ensemble predictions are averaged, majority-voted, or weighted, and how feature rankings are consolidated across ensemble members. A concise mathematical expression or pseudocode would improve clarity.

4. The process for generating synthetic samples from minority cell types is described conceptually but lacks implementation detail. Information about how many synthetic cells are generated per cluster, how latent variables are sampled, and how variance is controlled would make this component more transparent. Including a schematic or short equation would be beneficial.

5. Figure 4 uses dataset abbreviations such as "10x-MO," "CITE," and "TEA" without defining them in the caption. These abbreviations should be expanded or defined once in the main figure legend to ensure clarity for readers unfamiliar with the datasets.

6. Key training parameters-including optimizer type, learning rate, batch size, number of epochs, ensemble size, and any regularization factors-are not described systematically. These details are essential for reproducibility and fair comparison. A concise summary table of all hyperparameters would be helpful, ideally placed in the Supplementary Notes or Methods Appendix to maintain readability in the main text.

7. Minor grammatical and stylistic edits are recommended for precision and tone (e.g., "rare population" → "rare populations"; "a deep model" → "deep models"). The writing could also be slightly moderated-phrases such as "significantly outperforms" should be reserved for results supported by explicit statistical testing.

Reviewer #2:

Summary:
The authors present Hydra, a new deep learning framework for analyzing single-cell omics data. The work aims to tackle two significant challenges: the difficulty of annotating rare cell types and the "black box" nature of many deep learning models. The method uses an ensemble of variational autoencoders (VAEs) to learn the data's structure and then generates synthetic data for rare cell populations to create balanced datasets for training. It incorporates a technique called Integrated Gradients to identify the key molecular features defining each cell type, which makes the model interpretable. Finally, an ensemble of simple neural networks is trained on these top ranking features. The authors tested Hydra on 21 different datasets, showing it generally performs as well as or better than other state-of-the-art tools, with a clear advantage in identifying rare cells. The study is rounded out with a case study on Alzheimer's disease data, demonstrating the tool's robustness for complex biological questions.

The authors have performed a comprehensive and rigorous evaluation of Hydra. Using 21 distinct datasets that span multiple technologies, modalities, and tissues provides strong evidence for the method's generalizability and robustness. The paper is well-written and the figures clearly illustrate the framework.

Major issues:
1. The comparison baselines for the scRNA-seq annotation task could be updated to strengthen the claims of superiority. Among the baselines, the most recent method, scClassify, was published in 2020. The field is fast-moving, and including comparisons against more recent, state-of-the-art annotation methods would make the performance claims more persuasive.
2. The use of deep ensemble models raises significant questions about computational efficiency. The manuscript lacks an analysis of the computational resources (CPU/GPU time, memory) required to train and run Hydra, especially in comparison to the benchmarked methods. This is a critical practical limitation for its adoption by the research community.
3. A cornerstone of the method is the VAE-based generation of synthetic cells to augment rare populations. However, the quality of these synthetic cells is not evaluated. It is essential to demonstrate that the generated data faithfully recapitulates the biological identity of the rare cells without introducing artifacts.
4. The tool is set up for supervised annotation, meaning it can only find cell types it was trained on. A major real-world challenge is the presence of novel or unexpected cell types in a query sample. The manuscript does not discuss how Hydra would handle such cases or address this limitation.

Minor issues:
1. The authors should point out that it's a "cell type classification head" in Figure 1. The current description in Figure 1 and in the session "Overview of Hydra framework" is not clear for the classification head.
2. What are the differences between ensemble models 1 to M?
3. Since Integrated Gradients (IG) is a key concept in the feature ranking module, the authors should introduce this concept briefly in the Methods section.
4. A thorough proofread is recommended to catch minor typos. For example, there is a typo in "... a single model where no ensemble is not used."

Reviewer #3:

Hydra presents an ensemble VAE framework for interpretable single-cell feature selection and annotation. The method performs well empirically, especially on rare cell types, but the manuscript suffers from conceptual and methodological ambiguities which have to be clarified.
1. Sampling of Rare Populations
The approach to sampling rare cell populations for intra-dataset evaluation is not entirely clear. If it oversampled rare cell types, does this risk overfitting to rare cell types? Have the authors compared to across dataset and study evaluations?
2. Rank Aggregation via IG Method
The choice of the IG (Integrated Gradients) method for rank aggregation is intriguing. Could the authors elaborate on why this particular method was selected
3. Multi-Omics Data Processing
For multi-omics data, please clarify how the tool handles multiple modalities. Are modalities concatenated or linked through shared features? Does the model employ separate encoder-decoder structures for each modality, or a shared architecture?
4. Interpretability and Top-Ranked Features
The interpretability aspect seems primarily based on identifying top-ranked features. Interpretability for deep learning models based on purely feature ranking seems an over-claim: the challenging of interpret deep learning models with existing techniques are not about feature selection (SHAP-value, etc, can be used seamlessly) but how they interact to produce the final predictions.
5. Comparison with Recently Proposed Methods
Several recently proposed methods, such as the following, may serve as valuable benchmarks for comparison:
o https://www.biorxiv.org/content/10.1101/2025.05.06.652331v1.abstract
o https://assets-eu.researchsquare.com/files/rs-5754185/v1_covered_91fcffb4-96c9-430d-8c9d-ea32682e66cf.pdf
6. Inter-Dataset Comparison and Dataset Division
The inter-dataset comparison setup appears somewhat unclear. Based on Table 1, if training is performed on batch 1, each query dataset may still have partial overlap with the training data. Can the authors perform prediction on a completely unseen dataset to better evaluate cross-dataset generalization? If this is not the intended setup, please clarify the division scheme.
7. Biological Meaningfulness
It would strengthen the work to provide clearer biological interpretations or examples demonstrating that the identified patterns or features are biologically meaningful.
8. Scalability
Please comment on the computational scalability of the proposed method. How does runtime or memory usage scale with increasing cell numbers or modalities compared with baseline methods?
9. Label Robustness
How robust is the method to label noise or annotation errors? Since many real-world datasets contain imperfect labels, an experiment introducing controlled label perturbations could be informative.
10. Clarification on scVI vs. scANVI
Since scVI does not utilize labels, how are predictions generated for labeled tasks? Is the comparison actually with scANVI?

11. Single-Model Ablation Study and Downsampling
The section on single-model ablation could be clarified. Did the authors perform downsampling scheme? It may help to compare one model trained with downsampling and another without (original dataset) to avoid the ambiguity

Response Letter

Reviewer #1:

This manuscript introduces Hydra, a deep generative ensemble framework for cell type annotation and feature discovery in single-cell omics data. The study tackles a timely and important problem-the integration of multiple single-cell modalities and the recovery of rare cell types-and combines several interesting components, including ensemble VAEs and gradient-based feature ranking. The idea is creative and could make a meaningful contribution, especially in the rapidly evolving area of multi-omic data analysis. That said, a number of points need further clarification and support. Some of the central claims, particularly those around interpretability, feature attribution, and the fairness of the benchmarking, are not yet fully convincing. The evaluation metrics are also rather limited given the scope of the conclusions. Overall, this is a promising and well-motivated study, but the work would benefit from stronger methodological justification and a more comprehensive evaluation to fully substantiate its key claims.

Response: We thank the reviewer for their thoughtful review and positive assessment of our manuscript. Please find the responses to the points raised in detail below.

1. The manuscript emphasizes interpretability as one of Hydra's main innovations. However, the framework remains a deep neural network whose internal operations are not inherently interpretable. The claimed interpretability arises only post hoc, through Integrated Gradients-based feature attribution, which generates ranked lists of genes or features associated with each cell type. This output is conceptually similar to standard differential expression (DE) analyses that have long provided interpretable marker sets grounded in explicit statistical models. In this sense, Hydra's interpretability is relatively-improved compared to typical black-box neural networks, but not beyond what conventional DE-based approaches already offer. The manuscript would benefit from reframing interpretability as a supporting feature rather than a central methodological innovation.

Response:

We thank the reviewer for their insightful comment. We acknowledge that Hydra utilizes a post-hoc interpretability approach of Integrated Gradients rather than being intrinsically interpretable, and that this distinction was not clearly articulated in the original manuscript. We have now clarified this in the Discussion section of the manuscript to differentiate between these two approaches and to better position the role of post-hoc feature attribution within Hydra's framework. The revised text reads as follows:

"*Hydra attempts to address the challenge of interpretability of traditional deep learning models through post-hoc feature attribution via Integrated Gradients (Sundararajan et al., 2017). We note that with post-hoc interpretability, additional analyses are performed on trained neural networks to explain the model. This differs from intrinsic interpretability, where prior knowledge or biologically interpretable designs are directly incorporated into the model architecture (Lipton*

et al., 2018, Murdoch et al., 2019, Wagle et al., 2024). Further, post-hoc feature attribution techniques identify the most important features influencing predictions and not how features interact within the network to produce final outputs. Thus, the attribution approach employed in Hydra provides a ranked list of biologically meaningful cell type-specific features rather than complete mechanistic explanations of the model's internal computations. Nevertheless, these features retain practical utility for distinguishing cell populations and the ensemble deep learning approach further improves feature stability."

2. The feature selection component relies exclusively on Integrated Gradients (IG) to identify cell type-specific features, yet no justification is provided for this particular choice. Given the central role of these ranked features in downstream annotation, the decision is non-trivial. The authors should demonstrate why IG is preferred over other attribution methods such as SHAP, DeepLIFT, or simpler gradient saliency techniques. An ablation or comparative analysis showing the effect of these alternatives on feature stability, biological relevance, and annotation accuracy would substantiate the use of IG. Without such evidence, the gradient-based feature selection appears somewhat arbitrary rather than empirically motivated.

Response:

We thank the reviewer for their comment. To address this concern, we performed a comparative analysis of the Integrated Gradient (Sundararajan et al., 2017) feature attribution approach of Hydra against three other commonly used attribution techniques, including Saliency (Simonyan et al., 2013), GradientSHAP (Lundberg et al., 2017) and DeepLIFT (Shrikumar et al., 2017). We evaluated these techniques for the stability of selected features and cell type prediction performance. Our analysis reveals that all four attribution approaches provide comparable cell type prediction performance (Appendix Figure S2). However, Integrated Gradients consistently showed higher feature stability and lower variability across all cell types compared to Saliency, GradientSHAP, and DeepLIFT (Appendix Figure S2). Given its superiority in identifying reproducible cell type markers, we have adopted Integrated Gradients as the default feature selection approach in Hydra. We have now incorporated this in the Results section of the manuscript.

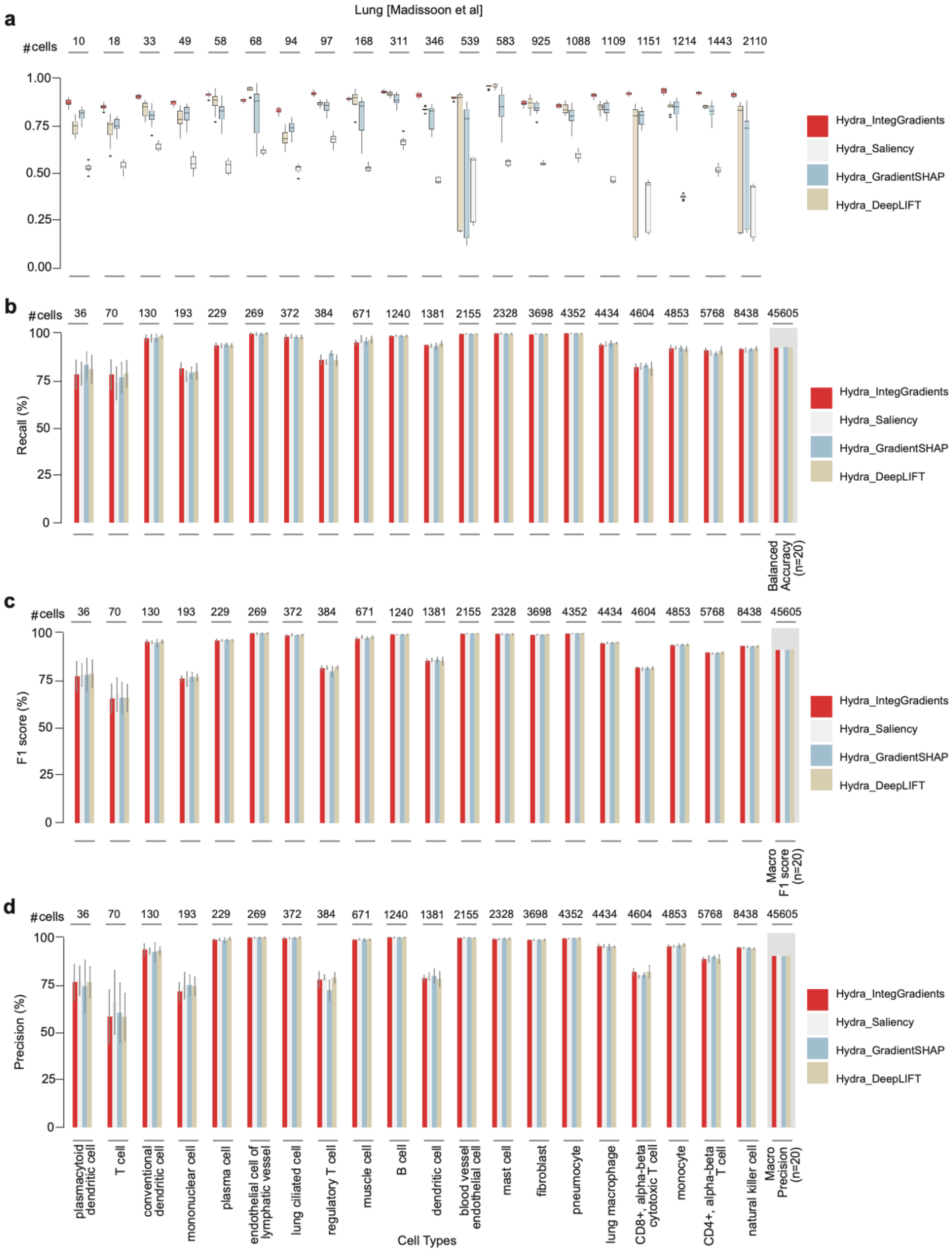

Lung [Madissoon et al]
a
b
c
d
#cells
Recall (%)
F1 score (%)
Precision (%)
Balanced Accuracy (n=20)
Macro F1 score (n=20)
Macro Precision (n=20)
Hydra_IntegGradients
Hydra_Saliency
Hydra_GradientSHAP
Hydra_DeepLIFT
plasmacytoid dendritic cell
T cell
conventional dendritic cell
mononuclear cell
plasma cell
endothelial cell of lymphatic vessel
lung ciliated cell
regulatory T cell
muscle cell
B cell
dendritic cell
blood vessel endothelial cell
mast cell
fibroblast
pneumocyte
lung macrophage
CD8+, alpha-beta cytotoxic T cell
monocyte
CD4+, alpha-beta T cell
natural killer cell
Cell Types

(a) Stability of features selected by different attribution approaches in Hydra (Integrated Gradients, Saliency, GradientSHAP, and DeepLIFT) for all cell types in the lung dataset (n=20), quantified by Pearson correlation coefficients. Cell types are arranged in the order of increasing sample count from left to right.

(b-d) Cell type prediction performance of different attribution approaches in Hydra evaluated using recall (b), F1 score (c) and precision (d). Cell types are arranged in the order of increasing sample count from left to right, followed by the overall performance (highlighted with a gray background)

3. Although Hydra is benchmarked against multiple existing methods across numerous datasets, the benchmarking design raises concerns about fairness and interpretability. It is unclear whether all baseline models were optimized under comparable conditions; the manuscript notes that default parameters were used for several tools, which may not reflect their optimal performance. Furthermore, the evaluation relies solely on overall accuracy, which can obscure performance differences among rare or ambiguous cell types. Including per-class precision, recall, and F1-scores would provide a clearer picture of the model's strengths and weaknesses. Finally, the computational cost of Hydra's ensemble framework is not reported, leaving scalability and runtime feasibility unaddressed.

Response:

We thank the reviewer for their suggestion. All baseline methods were implemented using their default parameters as recommended in the original publications and official documentation. These defaults reflect settings that were selected and validated by the original authors and are widely used in practice. To ensure fairness, we likewise fixed Hydra's hyperparameters across all datasets and evaluations, rather than tuning them on a per-dataset basis. This design avoids dataset- and method-specific overfitting and ensures that performance differences reflect methodological characteristics rather than differential parameter optimization.

We clarify that the “accuracy” reported in earlier versions of the manuscript corresponds to balanced accuracy, which is computed as the mean of per-class recalls. For each cell type, performance is calculated as True Positives / (True Positives + False Negatives), i.e. the proportion of correctly classified cells among all cells belonging to that type. This metric explicitly accounts for class imbalance and ensures that rare and abundant populations contribute equally to the overall score. To avoid ambiguity, we have now reworded this metric as *recall* throughout the manuscript and explicitly defined balanced accuracy as the average per-class recall. Additionally, to provide a more comprehensive evaluation of model performance, we have now included precision and F1-scores. The computations of these are included in the methodology section in the revised manuscript.

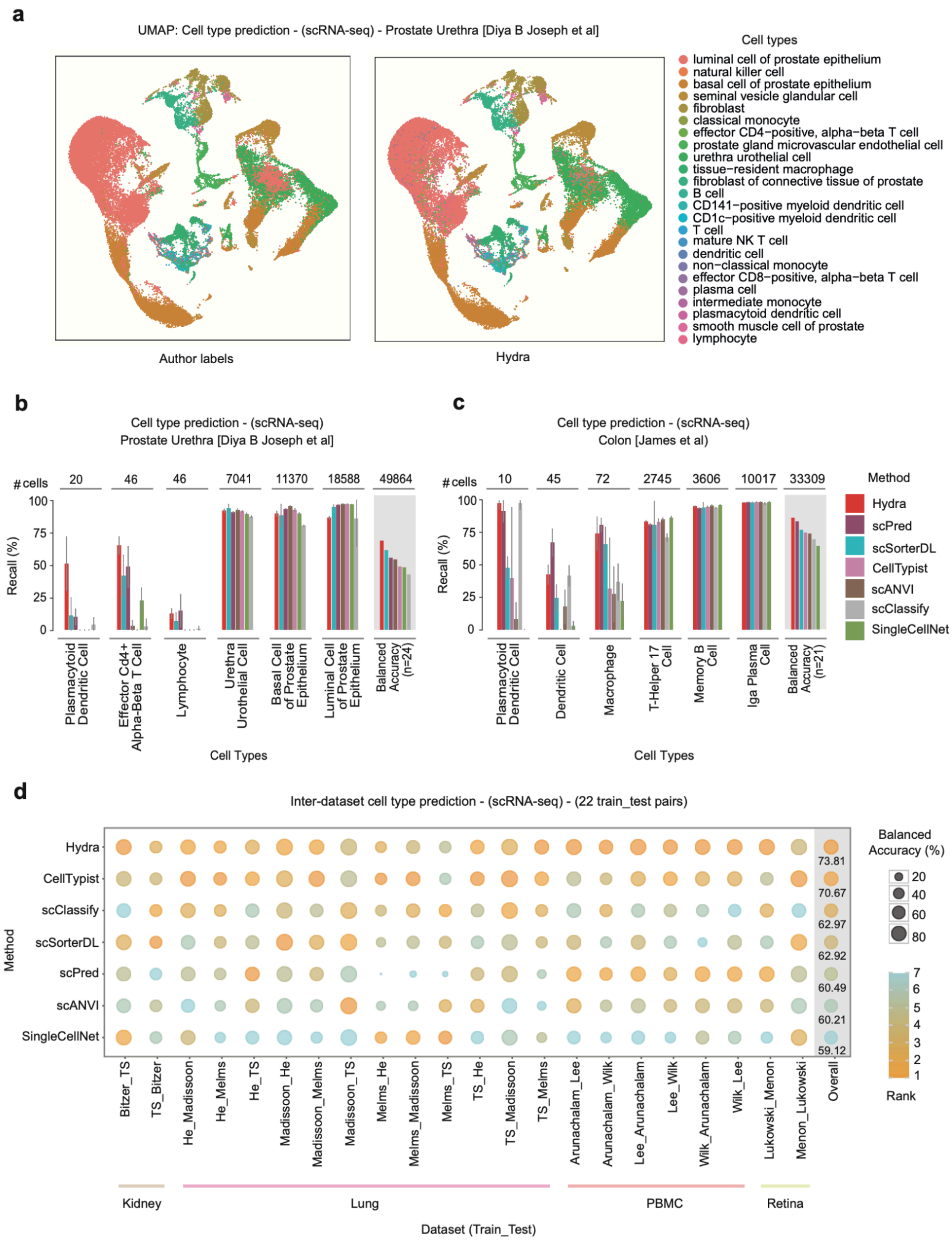
a
UMAP: Cell type prediction - (scRNA-seq) - Prostate Urethra [Diya B Joseph et al]
Author labels
Hydra
Cell types
luminal cell of prostate epithelium
natural killer cell
basal cell of prostate epithelium
seminal vesicle glandular cell
fibroblast
classical monocyte
effector CD4−positive, alpha−beta T cell
prostate gland microvascular endothelial cell
urethra urothelial cell
tissue−resident macrophage
fibroblast of connective tissue of prostate
B cell
CD141−positive myeloid dendritic cell
CD1c−positive myeloid dendritic cell
T cell
mature NK T cell
dendritic cell
non−classical monocyte
effector CD8−positive, alpha−beta T cell
plasma cell
intermediate monocyte
plasmacytoid dendritic cell
smooth muscle cell of prostate
lymphocyte
b
Cell type prediction - (scRNA-seq)
Prostate Urethra [Diya B Joseph et al]
cells
20
46
46
7041
11370
18588
49864
Recall (%)
Plasmacytoid Dendritic Cell
Effector Cd4+ Alpha-Beta T Cell
Lymphocyte
Urethra Urothelial Cell
Basal Cell of Prostate Epithelium
Luminal Cell of Prostate Epithelium
Balanced Accuracy (n=24)
Cell Types
c
Cell type prediction - (scRNA-seq)
Colon [James et al)
cells
10
45
72
2745
3606
10017
33309
Recall (%)
Plasmacytoid Dendritic Cell
Dendritic Cell
Macrophage
T-Helper 17 Cell
Memory B Cell
Iga Plasma Cell
Balanced Accuracy (n=21)
Cell Types
Method
Hydra
scPred
scSorterDL
CellTypist
scANVI
scClassify
SingleCellNet
d
Inter-dataset cell type prediction - (scRNA-seq) - (22 train_test pairs)
Method
Hydra
CellTypist
scClassify
scSorterDL
scPred
scANVI
SingleCellNet
73.81
70.67
62.97
62.92
60.49
60.21
59.12
Bitzer_TS
TS_Bitzer
He_Madissoon
He_Melms
He_TS
Madissoon_He
Madissoon_Melms
Madissoon_TS
Melms_He
Melms_Madissoon
Melms_TS
TS_He
TS_Madissoon
TS_Melms
Arunachalam_Lee
Arunachalam_Wilk
Lee_Arunachalam
Lee_Wilk
Wilk_Arunachalam
Wilk_Lee
Lukowski_Menon
Menon_Lukowski
Overall
Kidney
Lung
PBMC
Retina
Dataset (Train_Test)
Balanced Accuracy (%)
20
40
60
80
Rank
7
6
5
4
3
2
1

(a) Uniform Manifold Approximation and Projection (UMAP) plots for the single-cell transcriptomic prostate dataset, comprising 24 cell types and approximately 49,000 cells. The left panel shows the original labels as provided by the authors of the study, while the right panel shows the cell types predicted by Hydra.

(b) Bar plot with error bars illustrating the five-time repeated random subsampling intra-dataset cell type prediction performance of various methods using the single-cell transcriptomic prostate dataset. The three smallest and three largest cell types are displayed, arranged in order of increasing sample count from left to right, followed by the overall performance across all cell types in the dataset (highlighted with a gray background).

(c) Bar plot with error bars representing the five-time repeated random subsampling intra-dataset cell type prediction performance of different methods using a single-cell transcriptomic colon dataset comprising approximately 33,000 cells. The three smallest and three largest cell types are displayed, arranged in order of increasing sample count from left to right, followed by the overall performance across all 21 cell types in the dataset (highlighted with a gray background).

(d) Bubble plot illustrating the inter-dataset cell type prediction performance of various methods across four tissues (Kidney, Lung, PBMC, and Retina) and 22 train-test pairs. Methods are ranked based on their overall balanced accuracy across all train-test pairs.

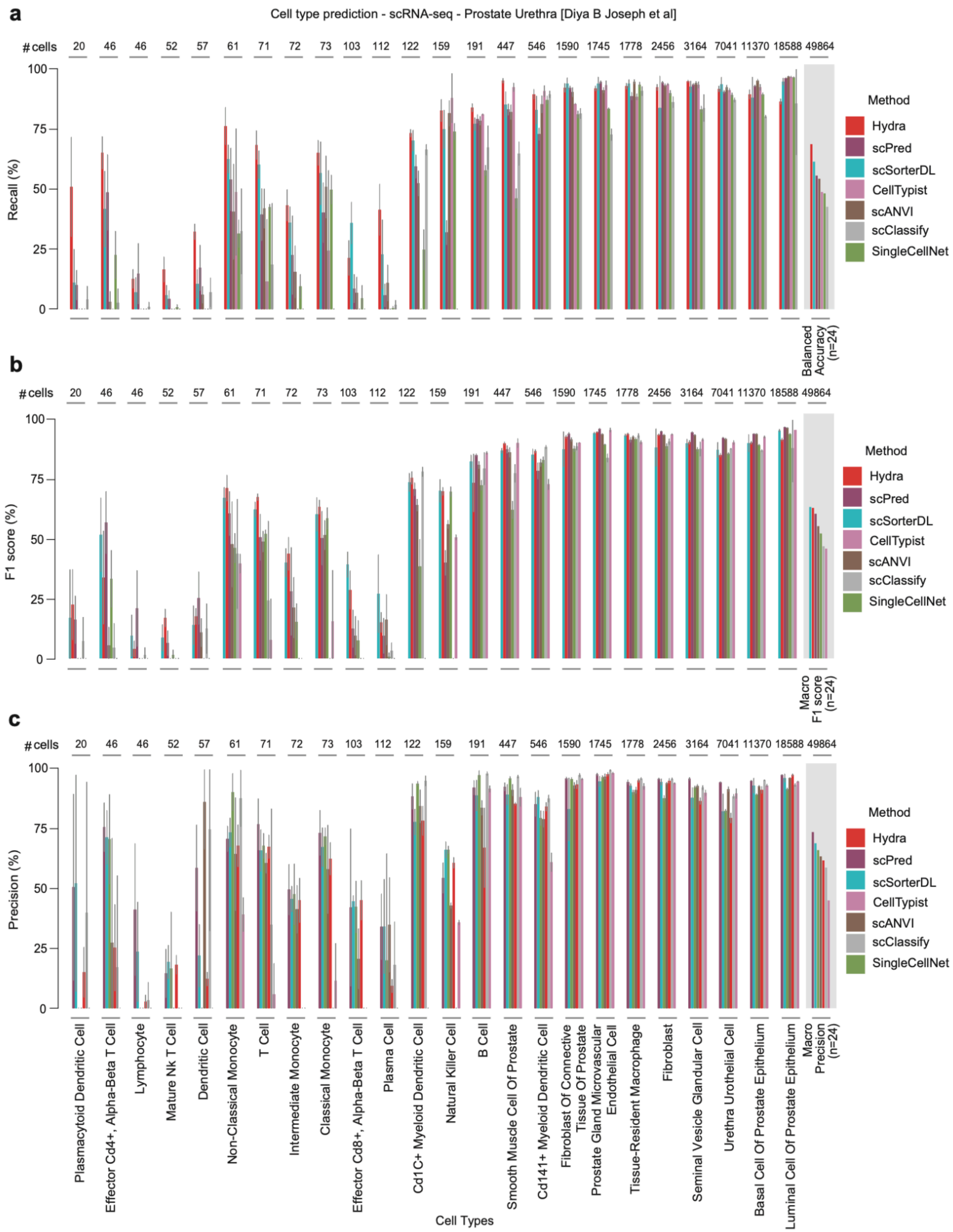
a
Cell type prediction - scRNA-seq - Prostate Urethra [Diya B Joseph et al]
cells
Recall (%)
Balanced Accuracy (n=24)
b
F1 score (%)
Macro F1 score (n=24)
c
Precision (%)
Macro Precision (n=24)
Method
Hydra
scPred
scSorterDL
CellTypist
scANVI
scClassify
SingleCellNet
Plasmacytoid Dendritic Cell
Effector Cd4+, Alpha-Beta T Cell
Lymphocyte
Mature Nk T Cell
Dendritic Cell
Non-Classical Monocyte
T Cell
Intermediate Monocyte
Classical Monocyte
Effector Cd8+, Alpha-Beta T Cell
Plasma Cell
Cd1C+ Myeloid Dendritic Cell
Natural Killer Cell
B Cell
Smooth Muscle Cell Of Prostate
Cd141+ Myeloid Dendritic Cell
Fibroblast Of Connective Tissue Of Prostate
Prostate Gland Microvascular Endothelial Cell
Tissue-Resident Macrophage
Fibroblast
Seminal Vesicle Glandular Cell
Urethra Urothelial Cell
Basal Cell Of Prostate Epithelium
Luminal Cell Of Prostate Epithelium
Cell Types

(a-c) Bar plot with error bars illustrating the five-time repeated random subsampling intra-dataset prediction performance evaluated using recall (a), F1 score (b) and precision(c) for all cell types using the scRNA-seq Prostate Urethra dataset [n=49k cells, 24 cell types]. Cell types are arranged in the order of increasing sample count from left to right, followed by the overall performance (highlighted with a gray background).

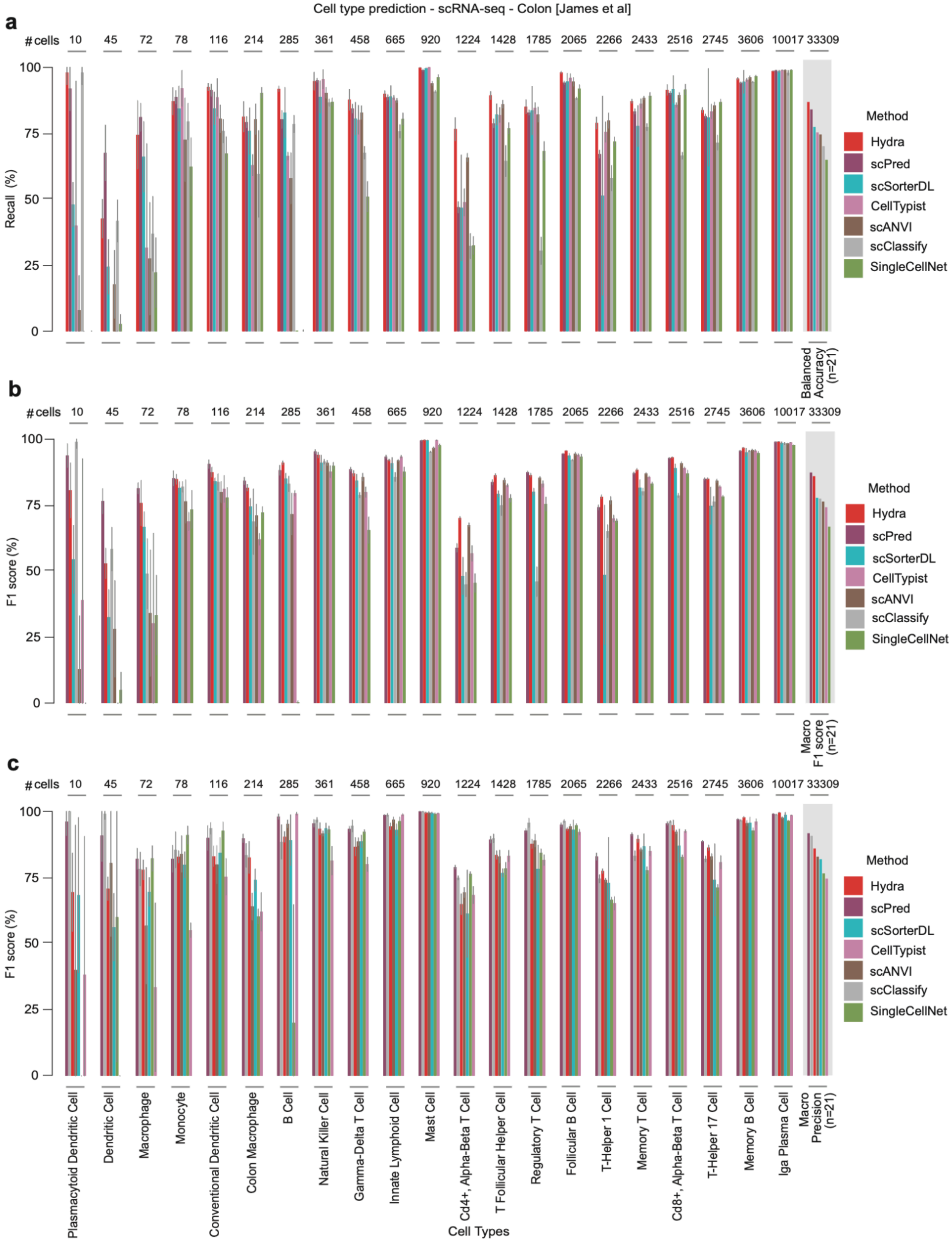
Cell type prediction - scRNA-seq - Colon [James et al]
a
b
c
cells
10 45 72 78 116 214 285 361 458 665 920 1224 1428 1785 2065 2266 2433 2516 2745 3606 10017 33309
Recall (%)
F1 score (%)
Balanced Accuracy (n=21)
Macro F1 score (n=21)
Macro Precision (n=21)
Method
Hydra
scPred
scSorterDL
CellTypist
scANVI
scClassify
SingleCellNet
Plasmacytoid Dendritic Cell
Dendritic Cell
Macrophage
Monocyte
Conventional Dendritic Cell
Colon Macrophage
B Cell
Natural Killer Cell
Gamma-Delta T Cell
Innate Lymphoid Cell
Mast Cell
Cd4+, Alpha-Beta T Cell
T Follicular Helper Cell
Regulatory T Cell
Follicular B Cell
T-Helper 1 Cell
Memory T Cell
Cd8+, Alpha-Beta T Cell
T-Helper 17 Cell
Memory B Cell
Iga Plasma Cell
Cell Types

(a-c) Bar plot with error bars illustrating the five-time repeated random subsampling intra-dataset prediction performance evaluated using recall (a), F1 score (b) and precision(c) for all cell types using the scRNA-seq Colon dataset [n=33k cells, 21 cell types]. Cell types are arranged in the order of increasing sample count from left to right, followed by the overall performance (highlighted with a gray background).

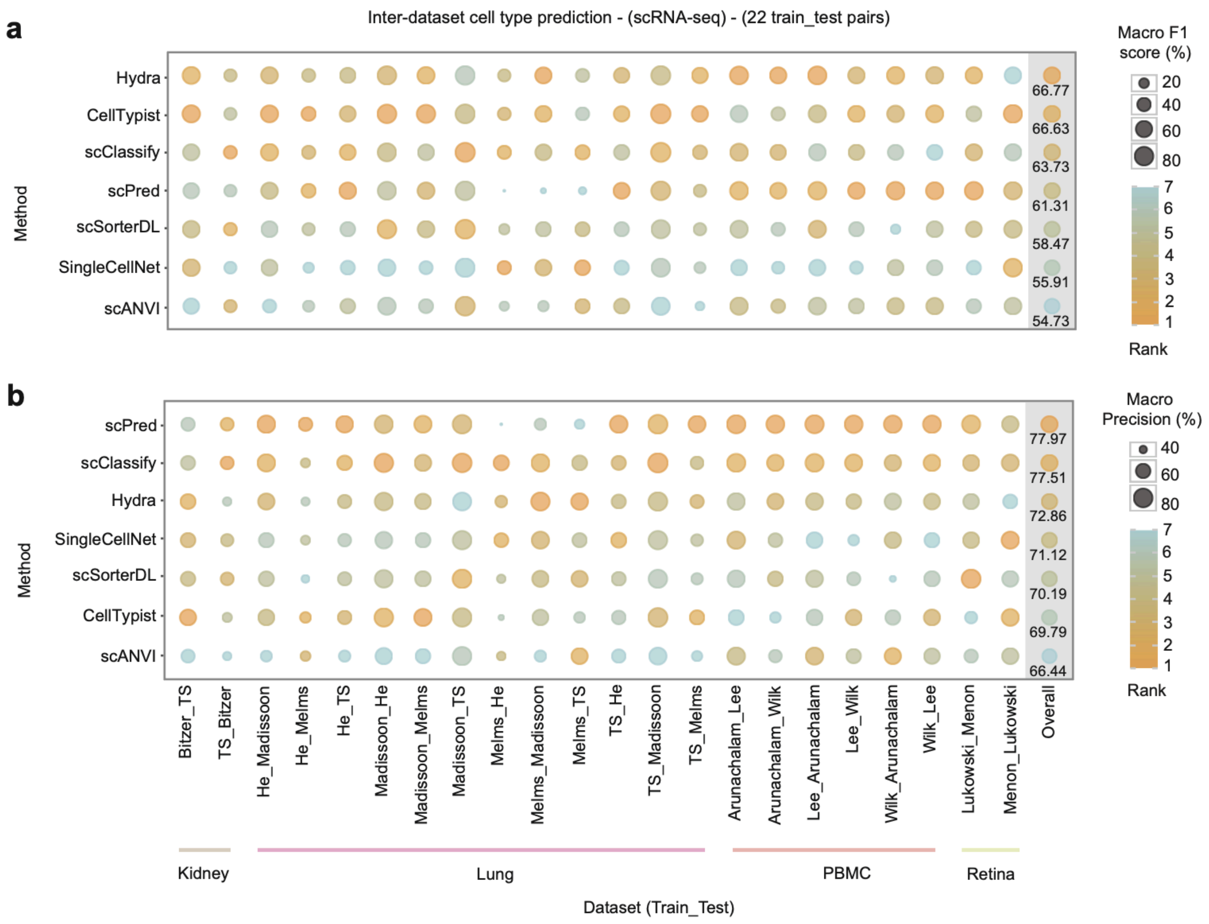

(a-b) Bubble plots illustrating the inter-dataset cell type prediction performance of various methods across four tissues (Kidney, Lung, PBMC, and Retina) and 22 train-test pairs evaluated using the macro F1 score (a) and macro precision (b). Methods are ranked based on their overall performance across all train-test pairs.

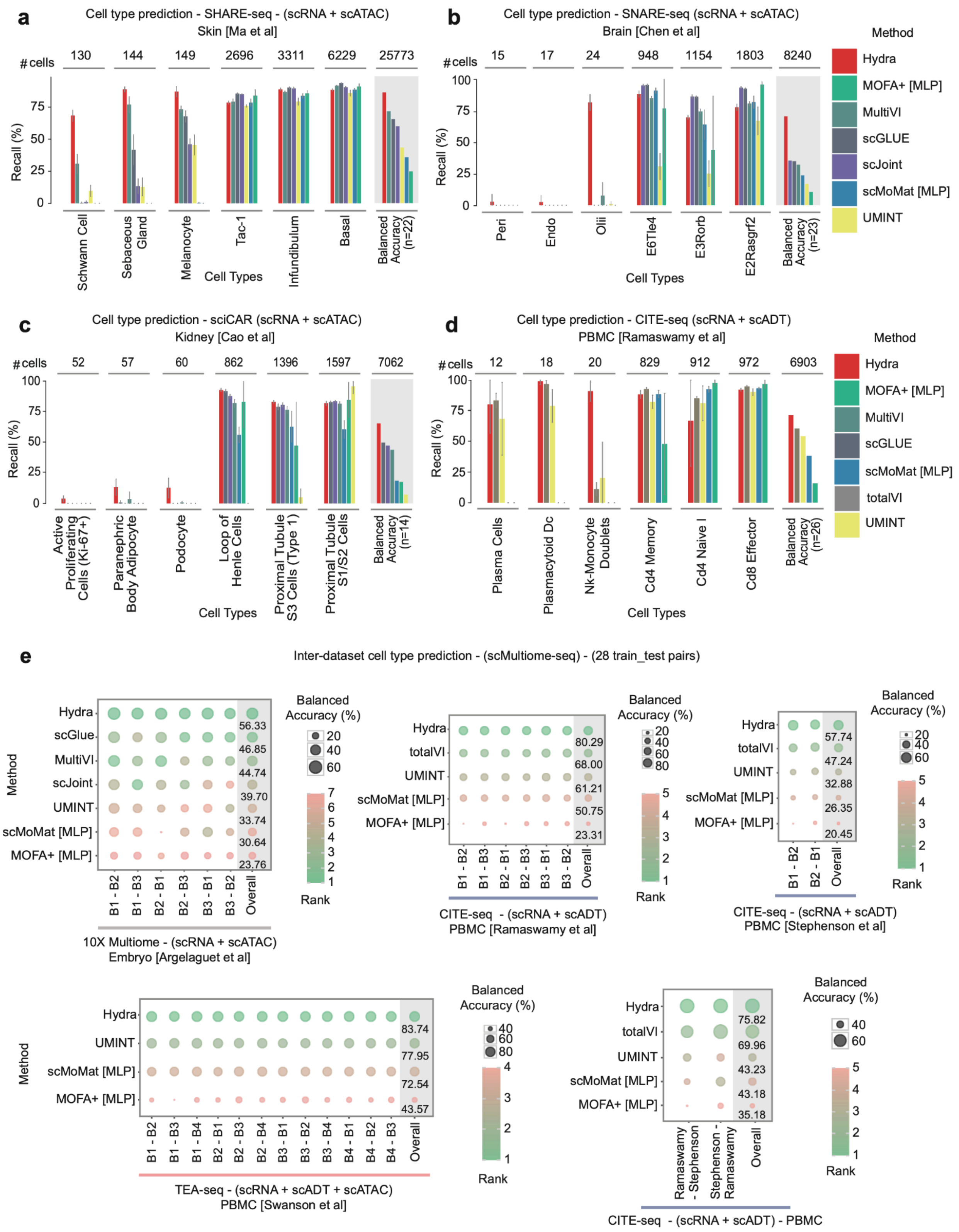

a
Cell type prediction - SHARE-seq - (scRNA + scATAC)
Skin [Ma et al]
cells
130
144
149
2696
3311
6229
25773
Recall (%)
Schwann Cell
Sebaceous Gland
Melanocyte
Tac-1
Infundibulum
Basal
Balanced Accuracy (n=22)
Cell Types
b
Cell type prediction - SNARE-seq (scRNA + scATAC)
Brain [Chen et al]
cells
15
17
24
948
1154
1803
8240
Recall (%)
Peri
Endo
Oli
E6Tle4
E3Rorb
E2Rasgrf2
Balanced Accuracy (n=23)
Cell Types
Method
Hydra
MOFA+ [MLP]
MultiVI
scGLUE
scJoint
scMoMat [MLP]
UMINT
c
Cell type prediction - sciCAR (scRNA + scATAC)
Kidney [Cao et al]
cells
52
57
60
862
1396
1597
7062
Recall (%)
Active Proliferating Cells (Ki-67+)
Paranephric Body Adipocyte
Podocyte
Loop of Henle Cells
Proximal Tubule S3 Cells (Type 1)
Proximal Tubule S1/S2 Cells
Balanced Accuracy (n=14)
Cell Types
d
Cell type prediction - CITE-seq (scRNA + scADT)
PBMC [Ramaswamy et al]
cells
12
18
20
829
912
972
6903
Recall (%)
Plasma Cells
Plasmacytoid Dc
Nk-Monocyte Doublets
Cd4 Memory
Cd4 Naive I
Cd8 Effector
Balanced Accuracy (n=26)
Cell Types
Method
Hydra
MOFA+ [MLP]
MultiVI
scGLUE
scMoMat [MLP]
totalVI
UMINT
e
Inter-dataset cell type prediction - (scMultiome-seq) - (28 train_test pairs)
Method
Hydra
scGlue
MultiVI
scJoint
UMINT
scMoMat [MLP]
MOFA+ [MLP]
56.33
46.85
44.74
39.70
33.74
30.64
23.76
B1 - B2
B1 - B3
B2 - B1
B2 - B3
B3 - B1
B3 - B2
Overall
Balanced Accuracy (%)
Rank
10X Multiome - (scRNA + scATAC)
Embryo [Argelaguet et al]
Hydra
totalVI
UMINT
scMoMat [MLP]
MOFA+ [MLP]
80.29
68.00
61.21
50.75
23.31
CITE-seq - (scRNA + scADT)
PBMC [Ramaswamy et al]
Hydra
totalVI
UMINT
scMoMat [MLP]
MOFA+ [MLP]
57.74
47.24
32.88
26.35
20.45
CITE-seq - (scRNA + scADT)
PBMC [Stephenson et al]
Hydra
UMINT
scMoMat [MLP]
MOFA+ [MLP]
83.74
77.95
72.54
43.57
B1 - B4
B2 - B4
B3 - B4
B4 - B1
B4 - B2
B4 - B3
TEA-seq - (scRNA + scADT + scATAC)
PBMC [Swanson et al]
Hydra
totalVI
UMINT
scMoMat [MLP]
MOFA+ [MLP]
75.82
69.96
43.23
43.18
35.18
Ramaswamy - Stephenson
Stephenson - Ramaswamy
CITE-seq - (scRNA + scADT) - PBMC

(a) Bar plot with error bars illustrating the 5-fold intra-dataset cell type prediction performance of Hydra against existing methods using the SHARE-seq (Simultaneous High-throughput ATAC and RNA Expression with sequencing) Skin dataset, comprising approximately 25,000 cells. Prediction accuracy for the three smallest and three largest cell types is displayed, followed by the overall performance across all 22 cell types in the dataset (highlighted with a gray background).

(b) Five-time repeated random subsampling intra-dataset cell type prediction accuracy of Hydra against existing methods using the SNARE-seq (Single-Nucleus Chromatin Accessibility and mRNA Expression sequencing) Brain dataset, comprising approximately 8,000 cells. The overall prediction performance across all 23 cell types is indicated with a gray background.

(c) Comparison of five-time repeated random subsampling intra-dataset cell type prediction accuracy of Hydra against other methods using the sciCAR-seq (Single-Cell Combinatorial Indexing chromatin accessibility and RNA sequencing) Kidney dataset, comprising approximately 7,000 cells. Overall performance across all 14 cell types in the dataset is highlighted with a gray background.

(d) Five-time repeated random subsampling intra-dataset cell type prediction performance using the CITE-seq (Cellular Indexing of Transcriptomes and Epitopes by sequencing) PBMC dataset, comprising approximately 6,000 cells. The overall performance across all 26 cell types in the dataset is highlighted with a gray background.

(e) Visualization of the inter-dataset cell type prediction performance of various methods across four distinct single-cell multiome studies: 10X Multiome (10X Chromium Epi Multiome ATAC + Gene Expression), CITE-seq, and TEA-seq (Transcriptome, Epitope, and Accessibility sequencing), spanning 28 train-test splits. The bubble plot ranks methods based on their overall performance.

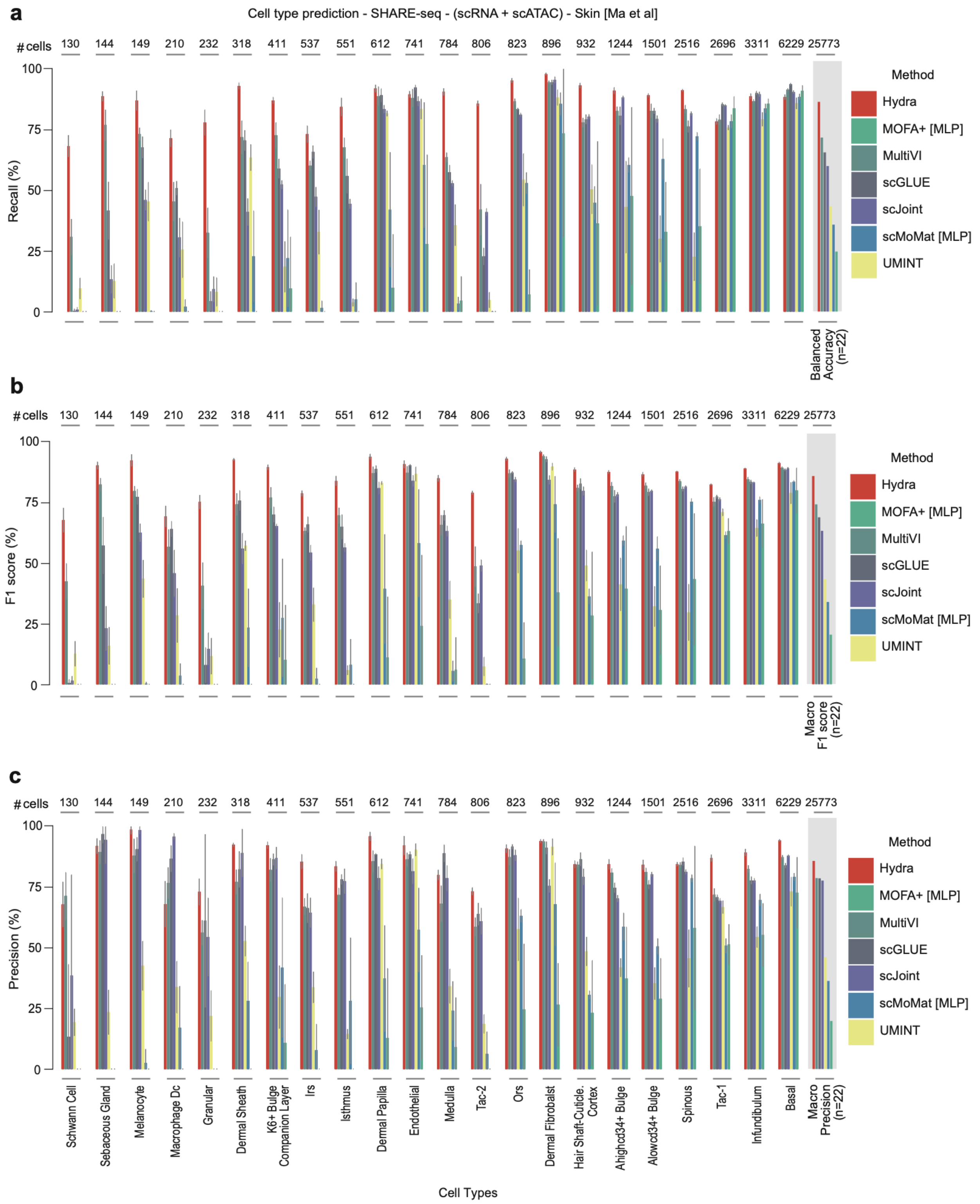

(a-c) Bar plot with error bars illustrating the five-time repeated random subsampling intra-dataset prediction performance evaluated using recall (a), F1 score (b) and precision (c) for all cell types using the SHARE-seq (Simultaneous High-throughput ATAC and RNA Expression with

sequencing) skin dataset [n=25k cells, 22 cell types]. Cell types are arranged in the order of increasing sample count from left to right, followed by the overall performance (highlighted with a gray background)

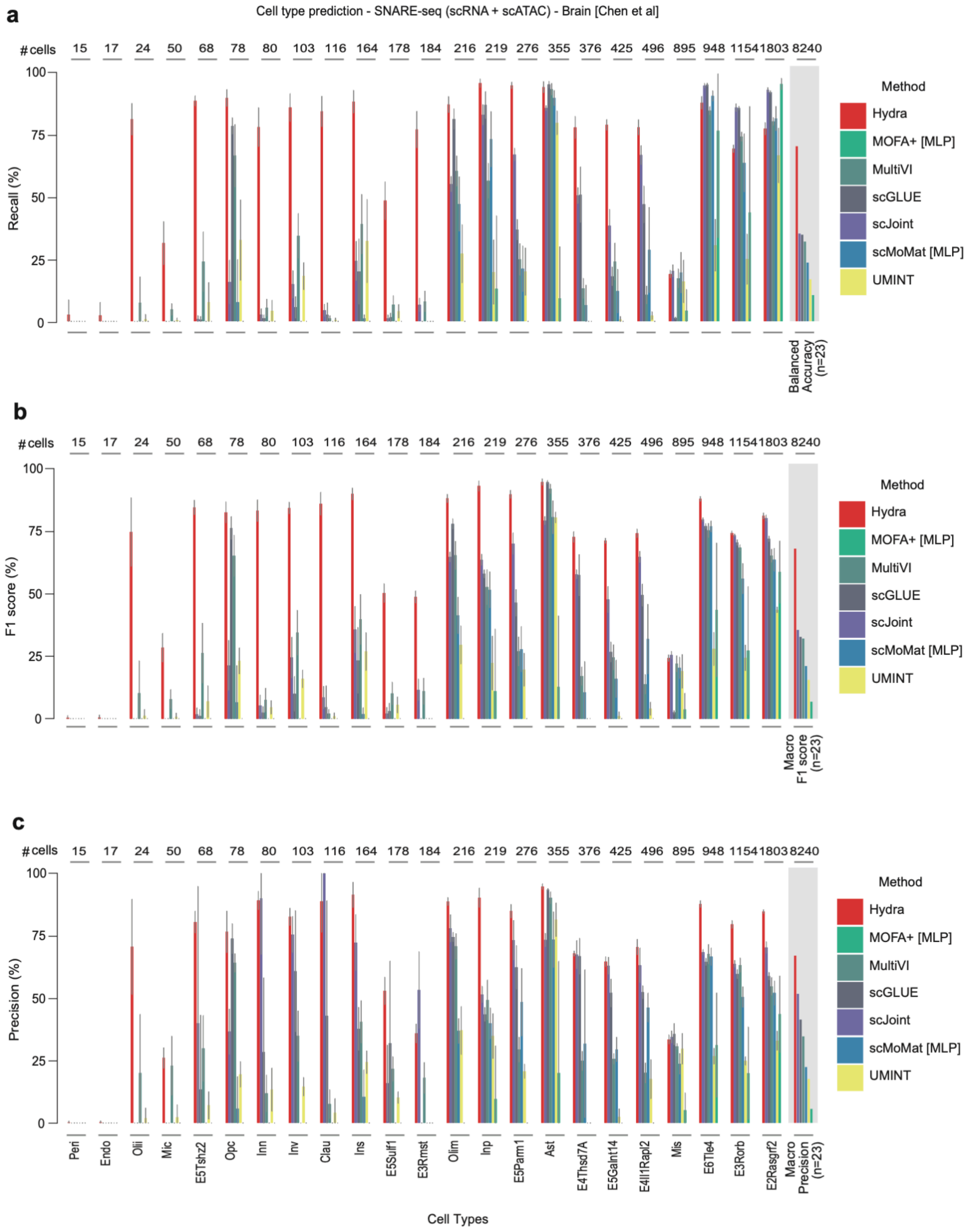

(a-c) Bar plot with error bars illustrating the five-time repeated random subsampling intra-dataset prediction performance evaluated using recall (a), F1 score (b) and precision (c) for all cell types

using the SNARE-seq (Single-Nucleus Chromatin Accessibility and mRNA Expression sequencing) Brain dataset [n=8k cells, 23 cell types]. Cell types are arranged in the order of increasing sample count from left to right, followed by the overall performance (highlighted with a gray background)

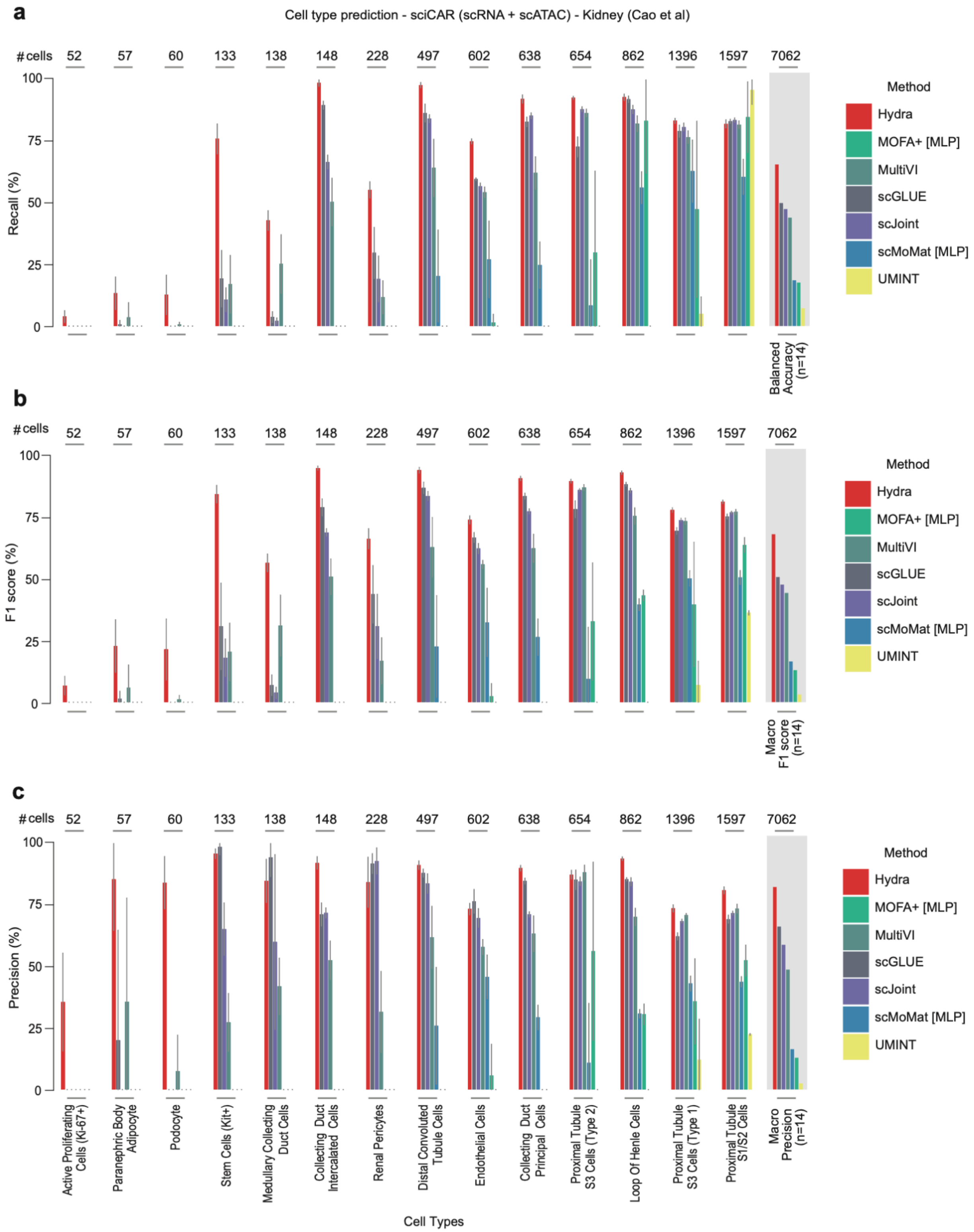

(a-c) Bar plot with error bars illustrating the five-time repeated random subsampling intra-dataset prediction performance evaluated using recall (a), F1 score (b) and precision (c) for all cell types

using the sciCAR-seq (Single-Cell Combinatorial Indexing chromatin accessibility and RNA sequencing) Kidney dataset [n=7k cells, 14 cell types]. Cell types are arranged in the order of increasing sample count from left to right, followed by the overall performance (highlighted with a gray background).

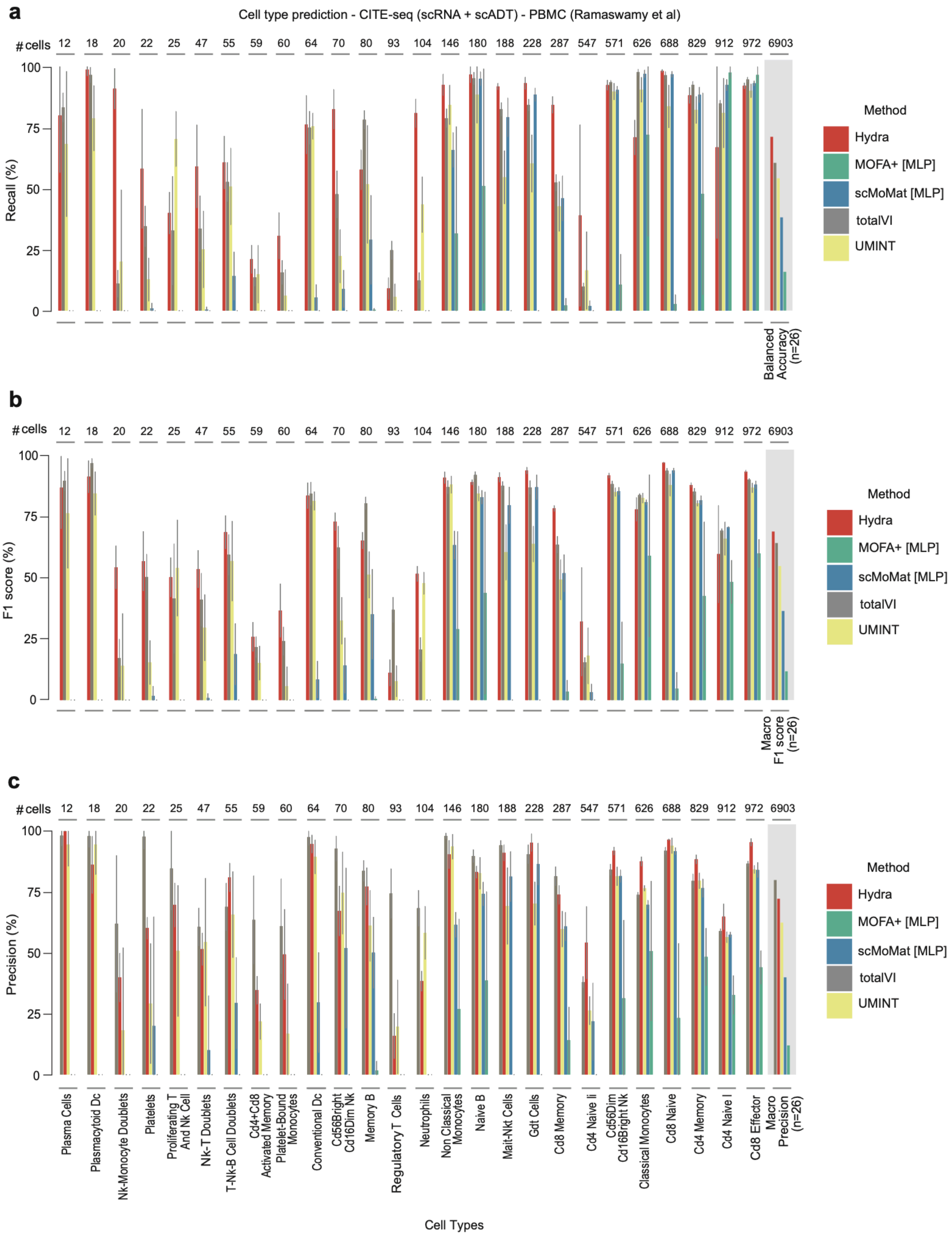

(a-c) Bar plot with error bars illustrating the five-time repeated random subsampling intra-dataset prediction performance evaluated using recall (a), F1 score (b) and precision(c) for all cell types

using the CITE-seq (Cellular Indexing of Transcriptomes and Epitopes by sequencing) PBMC dataset [n=6k cells, 26 cell types]. Cell types are arranged in the order of increasing sample count from left to right, followed by the overall performance (highlighted with a gray background).

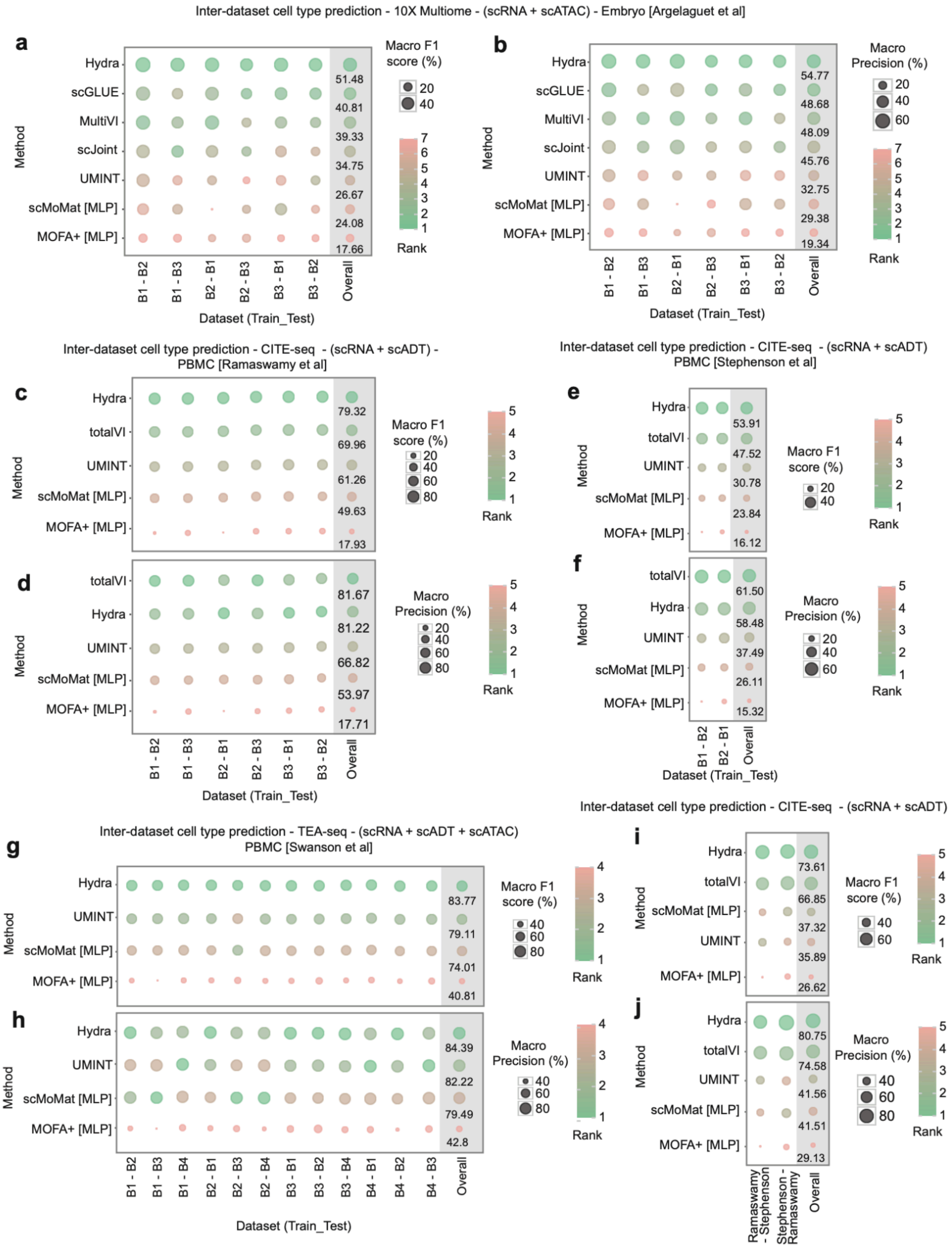
Inter-dataset cell type prediction - 10X Multiome - (scRNA + scATAC) - Embryo [Argelaguet et al]
a
Hydra
scGLUE
MultiVI
scJoint
UMINT
scMoMat [MLP]
MOFA+ [MLP]
Method
51.48
40.81
39.33
34.75
26.67
24.08
17.66
B1 - B2
B1 - B3
B2 - B1
B2 - B3
B3 - B1
B3 - B2
Overall
Dataset (Train_Test)
Macro F1 score (%)
20
40
Rank
b
Hydra
scGLUE
MultiVI
scJoint
UMINT
scMoMat [MLP]
MOFA+ [MLP]
54.77
48.68
48.09
45.76
32.75
29.38
19.34
Macro Precision (%)
20
40
60
Rank
Inter-dataset cell type prediction - CITE-seq - (scRNA + scADT) - PBMC [Ramaswamy et al]
c
Hydra
totalVI
UMINT
scMoMat [MLP]
MOFA+ [MLP]
79.32
69.96
61.26
49.63
17.93
Macro F1 score (%)
20
40
60
80
Rank
d
totalVI
Hydra
UMINT
scMoMat [MLP]
MOFA+ [MLP]
81.67
81.22
66.82
53.97
17.71
Macro Precision (%)
20
40
60
80
Rank
Inter-dataset cell type prediction - CITE-seq - (scRNA + scADT) PBMC [Stephenson et al]
e
Hydra
totalVI
UMINT
scMoMat [MLP]
MOFA+ [MLP]
53.91
47.52
30.78
23.84
16.12
Macro F1 score (%)
20
40
Rank
f
totalVI
Hydra
UMINT
scMoMat [MLP]
MOFA+ [MLP]
61.50
58.48
37.49
26.11
15.32
Macro Precision (%)
20
40
60
Rank
B1 - B2
B2 - B1
Overall
Dataset (Train_Test)
Inter-dataset cell type prediction - CITE-seq - (scRNA + scADT)
i
Hydra
totalVI
scMoMat [MLP]
UMINT
MOFA+ [MLP]
73.61
66.85
37.32
35.89
26.62
Macro F1 score (%)
40
60
Rank
j
Hydra
totalVI
UMINT
scMoMat [MLP]
MOFA+ [MLP]
80.75
74.58
41.56
41.51
29.13
Macro Precision (%)
40
60
80
Rank
Ramaswamy - Stephenson
Stephenson - Ramaswamy
Overall
Inter-dataset cell type prediction - TEA-seq - (scRNA + scADT + scATAC) PBMC [Swanson et al]
g
Hydra
UMINT
scMoMat [MLP]
MOFA+ [MLP]
83.77
79.11
74.01
40.81
Macro F1 score (%)
40
60
80
Rank
h
Hydra
UMINT
scMoMat [MLP]
MOFA+ [MLP]
84.39
82.22
79.49
42.8
Macro Precision (%)
40
60
80
Rank
B1 - B2
B1 - B3
B1 - B4
B2 - B1
B2 - B3
B2 - B4
B3 - B1
B3 - B2
B3 - B4
B4 - B1
B4 - B2
B4 - B3
Overall
Dataset (Train_Test)

Bubble plots showing inter-dataset cell type prediction performance across various methods, evaluated using macro F1 score and macro precision in 10X Multiome (10X Chromium Epi Multiome ATAC + Gene Expression) Embryo dataset (a-b), CITE-seq (Cellular Indexing of Transcriptomes and Epitopes by sequencing) PBMC [Ramaswamy et al] dataset (c-d), CITE-seq PBMC [Stephenson et al] dataset (e-f), TEA-seq (Transcriptome, Epitope, and Accessibility sequencing) PBMC dataset (g-h) and CITE-seq PBMC [Ramaswamy et al; Stephenson et al] datasets (i-j). Methods in each bubble plot are ranked by their overall performance.

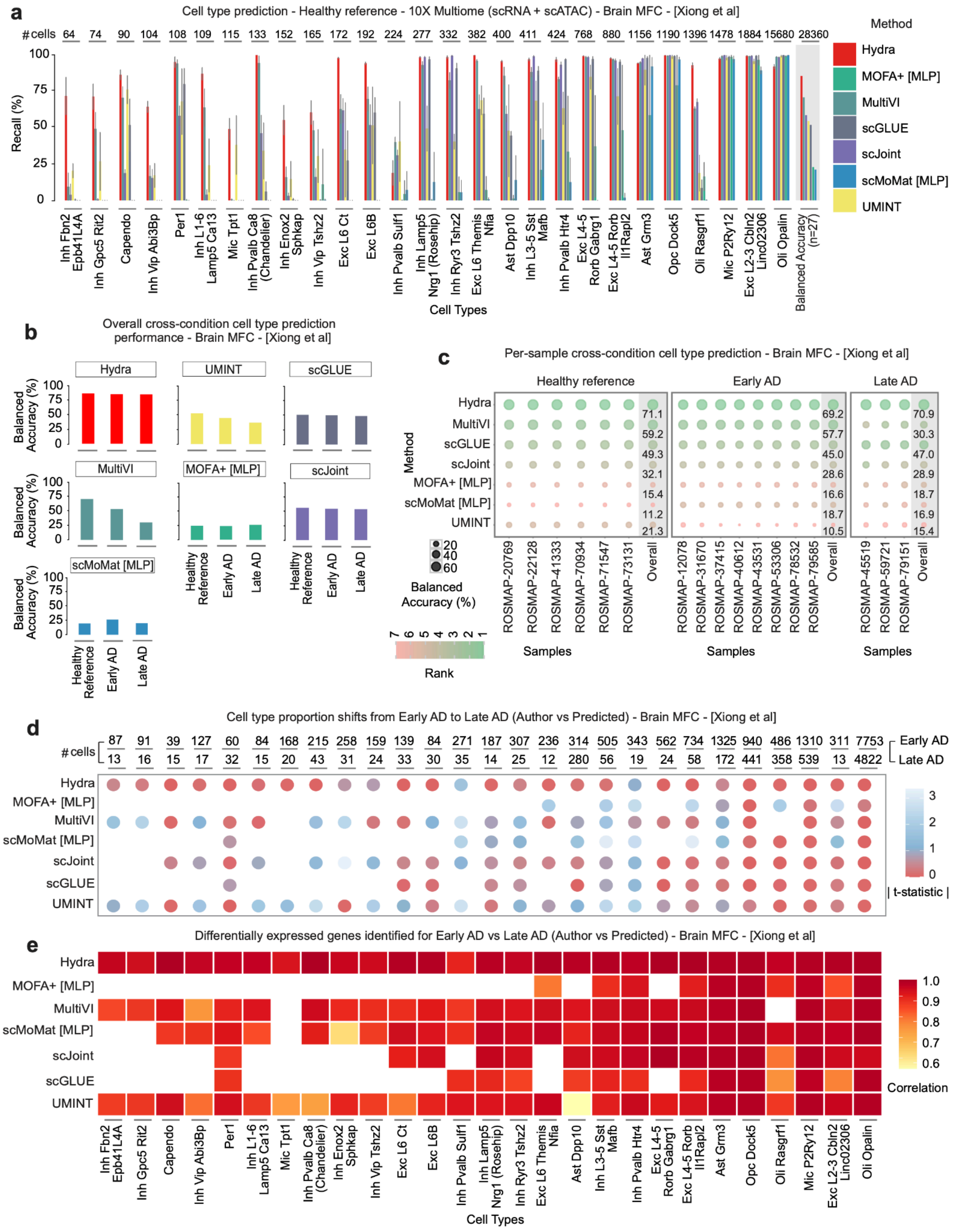

(a) Bar plot with error bars illustrating the 5-fold intra-dataset cell type prediction performance of Hydra against different single-cell multiome methods using healthy medial frontal cortex (MFC)

brain dataset. This dataset profiles RNA and ATAC modalities and comprises approximately 28,000 cells across 27 cell types. Prediction performance is displayed for all cell types, followed by the overall performance across all cell types in the dataset (highlighted with a gray background). Cell types are arranged in the order of increasing sample count from left to right.

(b) Faceted bar plots showing overall cell type prediction performance of different single-cell multiome methods across conditions trained on a healthy reference dataset, stratified by original cell type proportions. Error bars represent the variability in prediction accuracy across individual cell types.

(c) Bubble plot illustrating per-sample cross-condition identification of cell types using different single-cell multiome methods trained on healthy samples. The plot is faceted by conditions namely - healthy reference comprising held out healthy samples, early- and late-stage Alzheimer’s disease held out sample. Methods are ranked based on overall performance across all conditions.

(d) Bubble plot showing absolute t-statistics quantifying cell type proportion changes from early to late stages of Alzheimer's disease. Each circle represents the magnitude of proportion shift differences between author label and predicted label for each cell type.

(e) Heatmap illustrating Spearman correlation coefficients between differential gene expression t-statistics derived from author annotations and method predictions for early versus late AD.

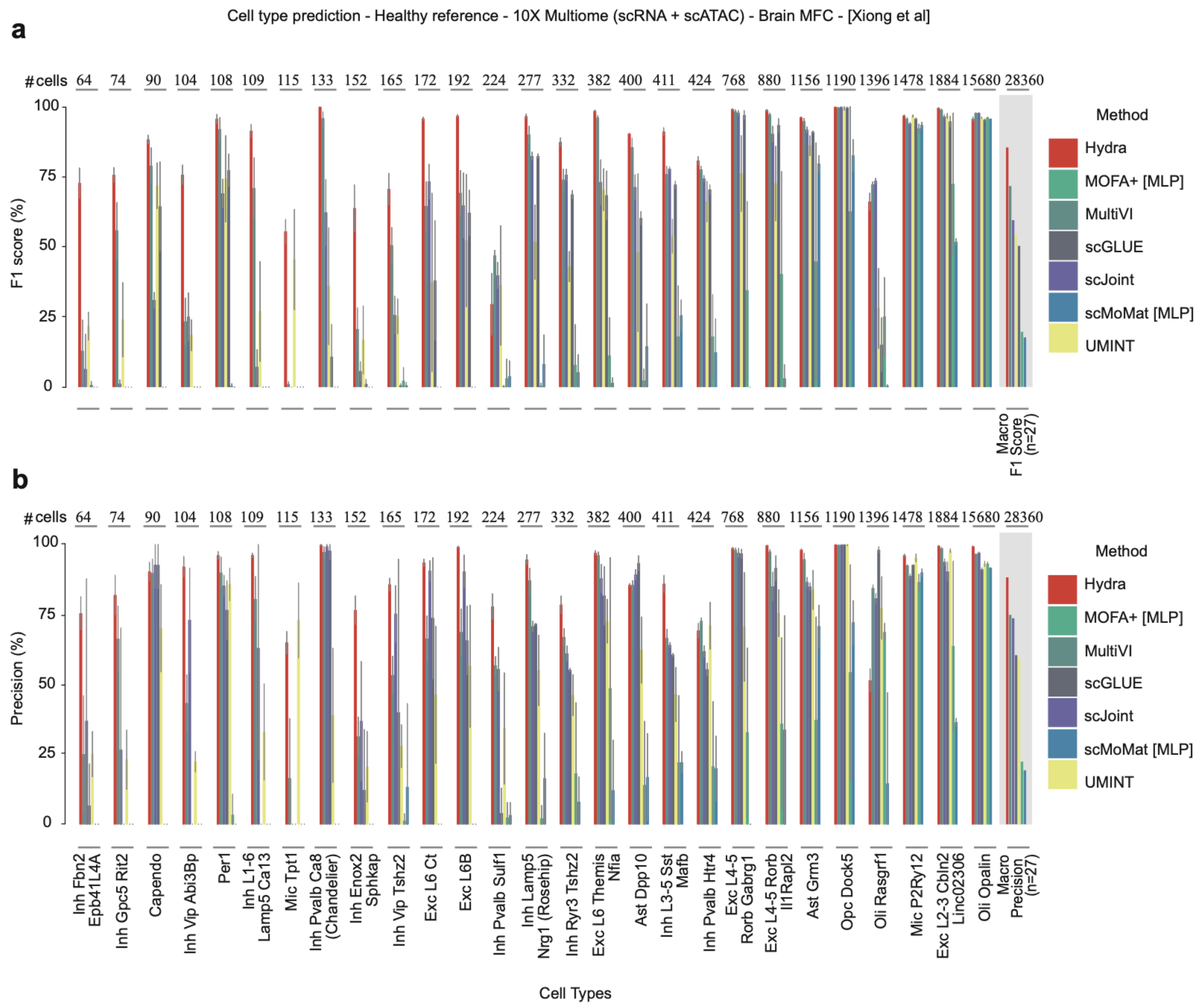

(a-b) Bar plot with error bars illustrating the 5-fold intra-dataset cell type prediction performance of Hydra against different single-cell multiome methods using healthy medial frontal cortex (MFC) brain dataset. Methods are evaluated using the F1 score (a) and precision (b). Prediction performance is displayed for all cell types, followed by the overall performance across all cell types (highlighted with a gray background). Cell types are arranged in the order of increasing sample count from left to right.

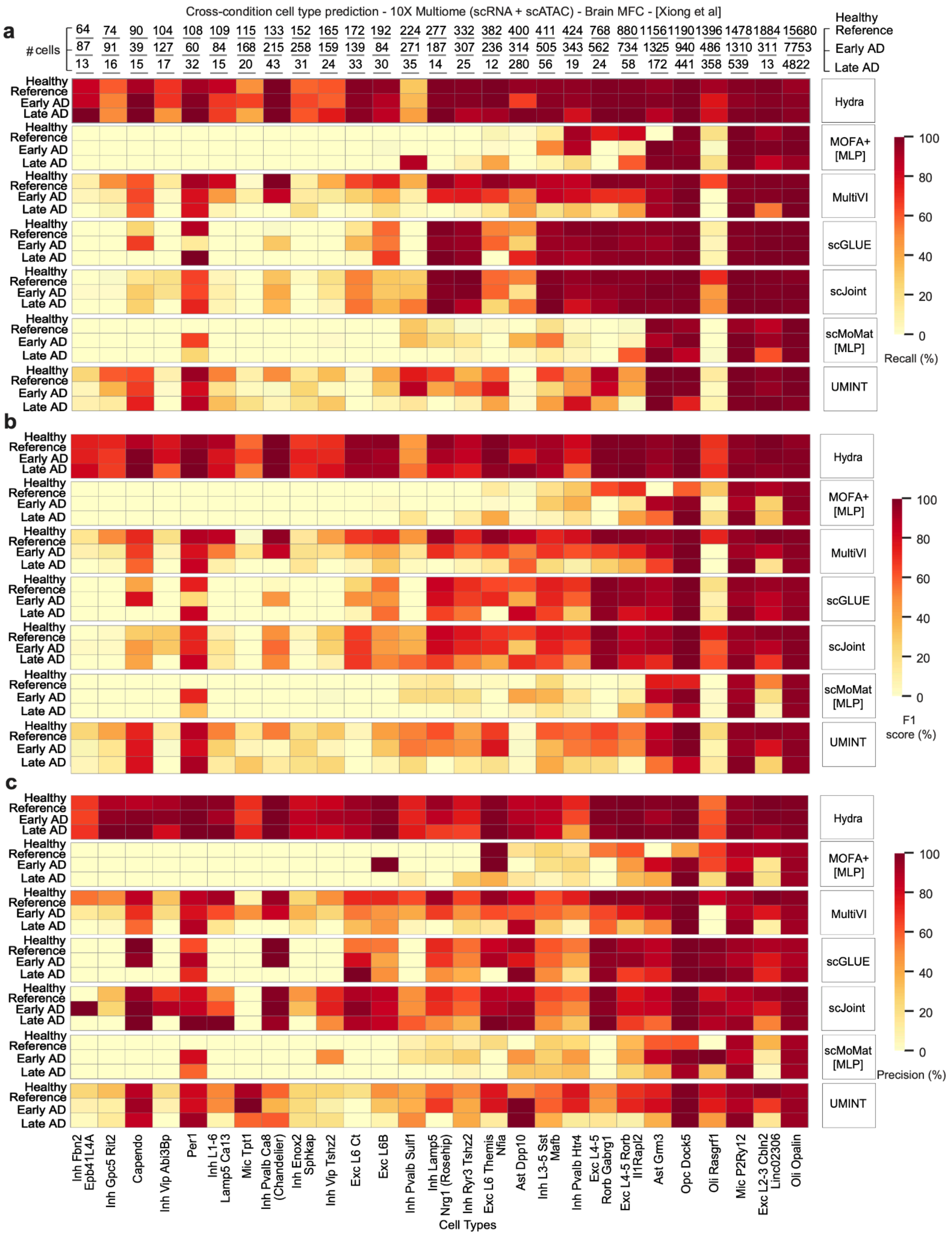

(a-c) Heatmaps showing cross-condition cell type prediction performance of different single-cell multiome methods using models trained on healthy reference dataset. Performance is evaluated

using recall (a), F1 score (b) and precision (c). Cell types are arranged in the order of increasing sample count from left to right based on healthy reference.

We thank the reviewer for their comment regarding the computational cost of Hydra's ensemble framework. To address this, we assessed runtime and GPU memory consumption across varying dataset sizes for both single-cell transcriptomic (Madissoon et al., 2019) and single-cell multiome data (Ma et al., 2020, Stephenson et al., 2021). Our evaluation shows that Hydra completed training in under 10 minutes across all benchmarked datasets (Fig. 6). Regarding GPU memory consumption, Hydra exhibited higher peak memory usage compared to baseline methods across all datasets. This can be attributed to its ensemble architecture which trains multiple variational autoencoder models to perform feature selection and improve prediction performance. These results demonstrate that Hydra's computational cost is feasible for adoption by the research community. We have now included these results in the revised manuscript.

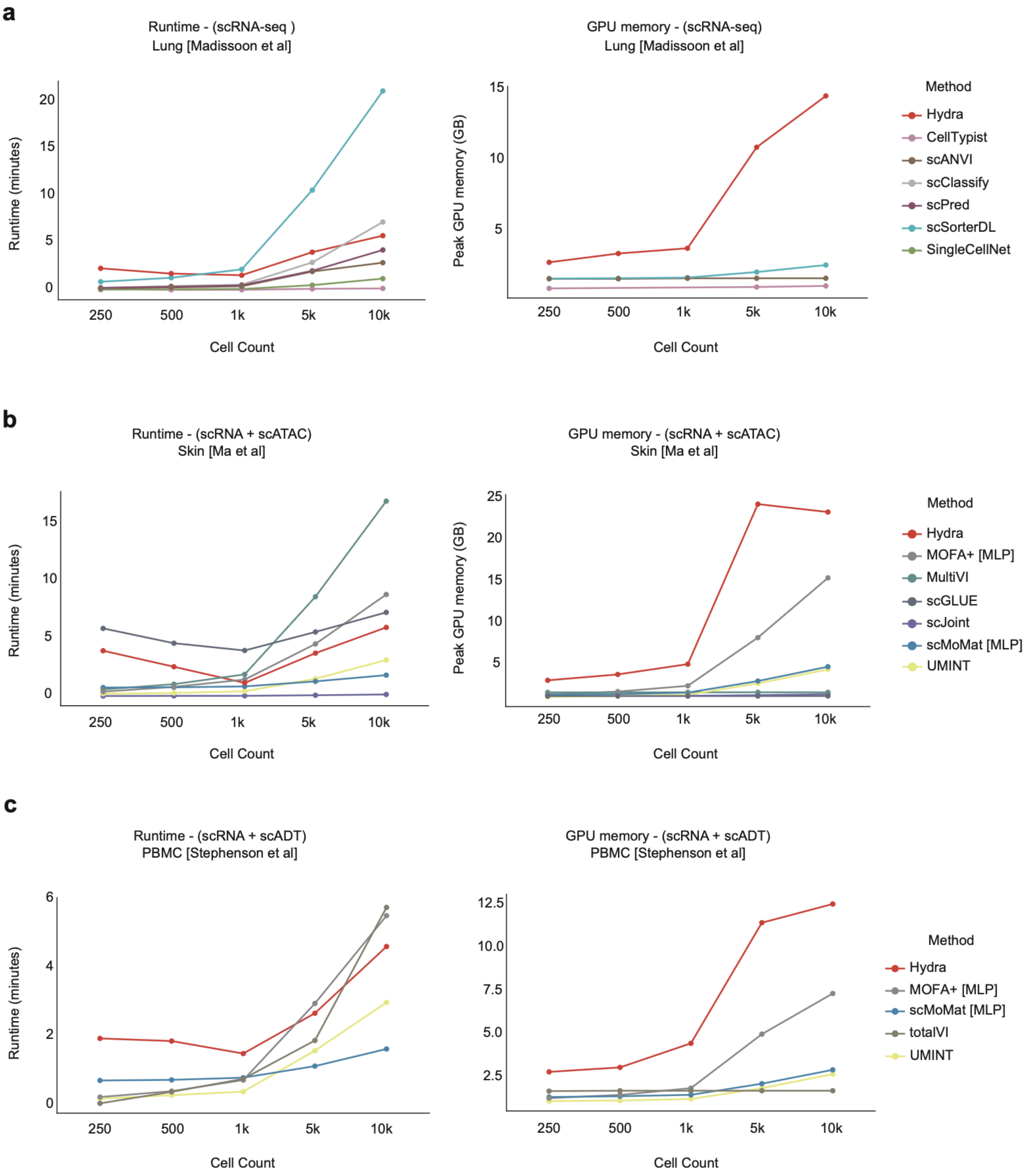

(a-c) Line plots showing runtime (left panel) and GPU consumption (right panel) of different methods across varying cell counts (250, 500, 1000, 5000, 10000). Evaluations were performed on single-cell transcriptomic data (a), joint profiling of single-cell transcriptomic and chromatin accessibility data (b) and joint profiling of single-cell transcriptomic and surface protein data (c)

4. Hydra's central claim lies in its ability to integrate and annotate multi-omic data. However, the multimodal benchmarking includes only MOFA+, UMINT, and scMoMaT-tools primarily designed for unsupervised embedding or factorization rather than supervised annotation or marker discovery. Several high-impact, state-of-the-art frameworks directly relevant to multi-omic integration and cell type annotation are omitted. These include scGLUE (Nat Biotechnol 2022), scCross (Genome Biol 2024), scJoint (Nat Biotechnol 2022), totalVI / MultiVI (Nat Methods 2020/2023). Benchmarking against these methods would provide a more rigorous and contemporary comparison, allowing Hydra's multi-omic claims to be evaluated in the context of current standards.

Response:

We thank the reviewer for this suggestion. To strengthen the benchmarking and better reflect the current state of the field, we have now expanded our comparisons to include the suggested state-of-the-art methods for single-cell multiome cell type prediction. Specifically, we have now additionally included scGLUE (Cao et al., 2022), scJoint (Lin et al., 2022), totalVI (Gayoso et al., 2021), and MultiVI (Ashuach et al., 2023) in our evaluation. We were unable to include scCross in our benchmarking as it required prohibitively long computational time that made systematic evaluation across multiple datasets not feasible. All results and figures have been updated in the revised manuscript to reflect these changes. We thank the reviewer again for encouraging this broader evaluation, which has strengthened our benchmarking.

5. The evaluation framework relies almost entirely on overall cell type classification accuracy. While this metric is intuitive, it is insufficient to support the breadth of claims made in the manuscript. Overall accuracy tends to be dominated by abundant populations and does not capture performance on rare or underrepresented cell types-one of Hydra's stated strengths. Reporting per-class precision, recall, or F1-scores would yield a more balanced assessment. For cross-dataset evaluations, metrics such as Adjusted Rand Index (ARI) or Normalized Mutual Information (NMI) would better quantify label concordance and generalization. Moreover, given Hydra's emphasis on multi-omic integration, standard integration-quality measures such as LISI, silhouette width, or graph connectivity should be included to assess modality alignment. Finally, for interpretability, a quantitative comparison of Hydra-identified markers against known cell-type gene sets or enrichment results would strengthen biological validation.

Response:

We appreciate the reviewer’s comment regarding the evaluation framework and the need for stronger biological validation of features selected by Hydra. This concern is addressed by our evaluation framework, as detailed in our response to point 3. Briefly, the primary metric used throughout the manuscript is balanced accuracy, defined as the average of per-cell-type recall, ensuring that rare and abundant populations contribute equally. In addition, precision and F1-scores are now reported to provide a more complete assessment of model performance (Fig. 3-5, Appendix Fig. S5-S14).

We clarify that Hydra is not designed to optimize low-dimensional embeddings and does not perform modality alignment. Rather, its use of multimodal data is intended to enable multimodal feature selection. For this reason, the evaluation of embedding using metrics such as ARI or NMI is not directly aligned with Hydra's design objective. Instead, as described in our response to point 4, we have expanded the benchmarking to include more recent state-of-the-art multimodal methods and evaluated all methods using additional performance metrics. Finally, to strengthen biological validation as suggested, we have now conducted gene ontology enrichment analysis on features selected by Hydra for three representative cell types - naive B cells, neutrophils, and mesenchymal cells, using the Tabula Muris dataset (Tabula Muris Consortium et al., 2018). The results demonstrated that Hydra captures functionally coherent biological processes that align with the established roles of each cell type. We have now discussed this in the Results section of the manuscript. The added text reads as follows:

"*Finally, to evaluate whether features selected by Hydra capture biologically meaningful molecular processes, we performed gene ontology enrichment analysis for three representative cell types - naive B cells, neutrophils, and mesenchymal cells. Specifically, we used Enrichr (Kuleshov et al., 2016) on the top 100 features from each cell type and tested for enrichment (Appendix Fig. S4). For naive B cells, some of the relevant enriched terms included B cell activation, B cell receptor signaling pathway, and regulation of B cell proliferation, consistent with their adaptive immune functions. For neutrophils, enriched terms included neutrophil migration, neutrophil chemotaxis, inflammatory response and granulocyte chemotaxis, reflecting innate immune activation processes. Mesenchymal cells showed enrichment for extracellular matrix organization, collagen fibril organization, and skeletal system development, consistent with their structural role. These results demonstrate Hydra's ability to select functionally relevant and interpretable signatures that reflect core cell type-specific functions.*"

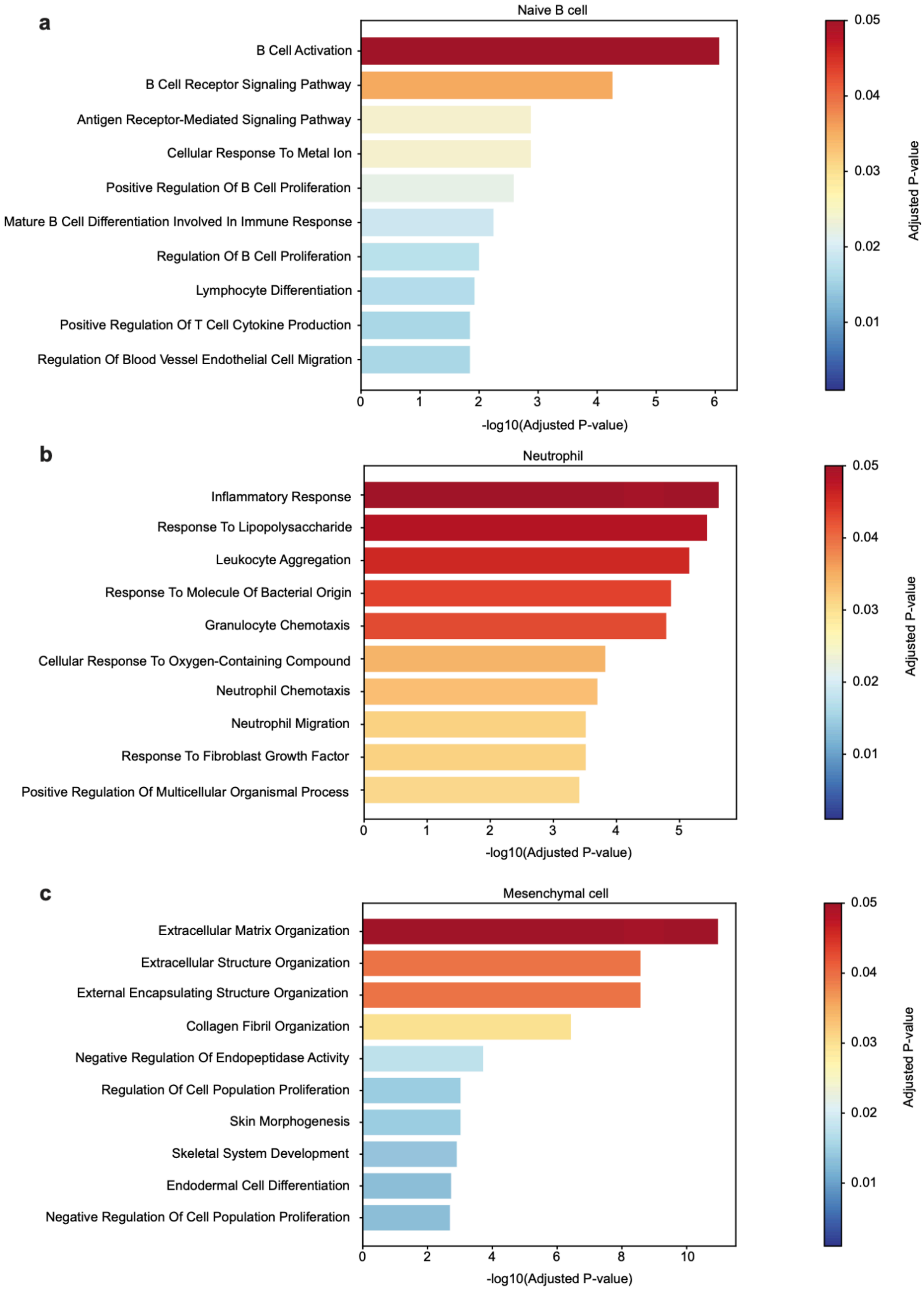
a
Naive B cell
B Cell Activation
B Cell Receptor Signaling Pathway
Antigen Receptor-Mediated Signaling Pathway
Cellular Response To Metal Ion
Positive Regulation Of B Cell Proliferation
Mature B Cell Differentiation Involved In Immune Response
Regulation Of B Cell Proliferation
Lymphocyte Differentiation
Positive Regulation Of T Cell Cytokine Production
Regulation Of Blood Vessel Endothelial Cell Migration
-log10(Adjusted P-value)
Adjusted P-value
b
Neutrophil
Inflammatory Response
Response To Lipopolysaccharide
Leukocyte Aggregation
Response To Molecule Of Bacterial Origin
Granulocyte Chemotaxis
Cellular Response To Oxygen-Containing Compound
Neutrophil Chemotaxis
Neutrophil Migration
Response To Fibroblast Growth Factor
Positive Regulation Of Multicellular Organismal Process
-log10(Adjusted P-value)
Adjusted P-value
c
Mesenchymal cell
Extracellular Matrix Organization
Extracellular Structure Organization
External Encapsulating Structure Organization
Collagen Fibril Organization
Negative Regulation Of Endopeptidase Activity
Regulation Of Cell Population Proliferation
Skin Morphogenesis
Skeletal System Development
Endodermal Cell Differentiation
Negative Regulation Of Cell Population Proliferation
-log10(Adjusted P-value)
Adjusted P-value

(a-c) Horizontal bar plots showing the top 10 significantly enriched terms (adjusted p-value < 0.05) for naive B cells (a), neutrophils (b), and mesenchymal cells (c). Enrichment analysis was performed using the top 100 features selected by Hydra for each cell type. Bar length represents -log10(adjusted p-value), and color intensity represents the significance level of enrichment.

6. The manuscript does not clearly describe how the reference cell-type labels were defined across multi-omic datasets. If these annotations originate primarily from the RNA modality-as is typical for multimodal assays such as 10x Multiome or CITE-seq-then the evaluation is inherently biased toward RNA-driven models. This introduces a conceptual problem: models that genuinely integrate additional modalities (e.g., ATAC or ADT) may deviate from RNA-based labels even when capturing more comprehensive biological information. Consequently, better integration of multi-omic signals could paradoxically reduce apparent "annotation accuracy," since the ground-truth labels reflect only one modality. The authors should clarify the provenance of the reference annotations and discuss the potential for such bias. An analysis showing how each modality contributes to Hydra's predictions would help disentangle genuine cross-modal integration from RNA-centric agreement.

Response:

We thank the reviewer for this thoughtful comment. We acknowledge that the current single-cell multiome evaluations used in the study can be biased toward RNA-based annotations, as reference labels in our benchmarked datasets are derived primarily from transcriptomic profiles. As such, benchmarking methods against these labels can introduce an inherent RNA-centric bias.

To examine how individual modalities contribute to Hydra's predictions under this constraint, we performed an ablation analysis using the 10X Multiome Brain medial frontal cortex dataset (Xiong et al., 2023). Specifically, we trained Hydra using RNA-only, ATAC-only, and joint RNA+ATAC inputs, while evaluating predictions against the RNA-derived reference labels. As expected given the provenance of the labels, ATAC-only models achieved substantially lower overall accuracy and failed to reliably identify most cell types. In contrast, joint RNA+ATAC models showed comparable overall performance to RNA-only models but yielded improved prediction accuracy for a subset of rare cell populations (n < 100), including *Inh FBN2 EPB41L4A*, *Inh GPC5 RIT2*, and *capEndo*. This suggests that chromatin accessibility may provide complementary discriminative information that can enhance the identification of low-abundance populations, even when evaluation is anchored to RNA-based labels.

As the reviewer correctly points out, methods that more fully integrate additional modalities may diverge from RNA-centric annotations, thereby potentially underestimating their true performance under current evaluation frameworks. We have now discussed the limitations of RNA-centric ground truth labels in the Discussion section of the revised manuscript.

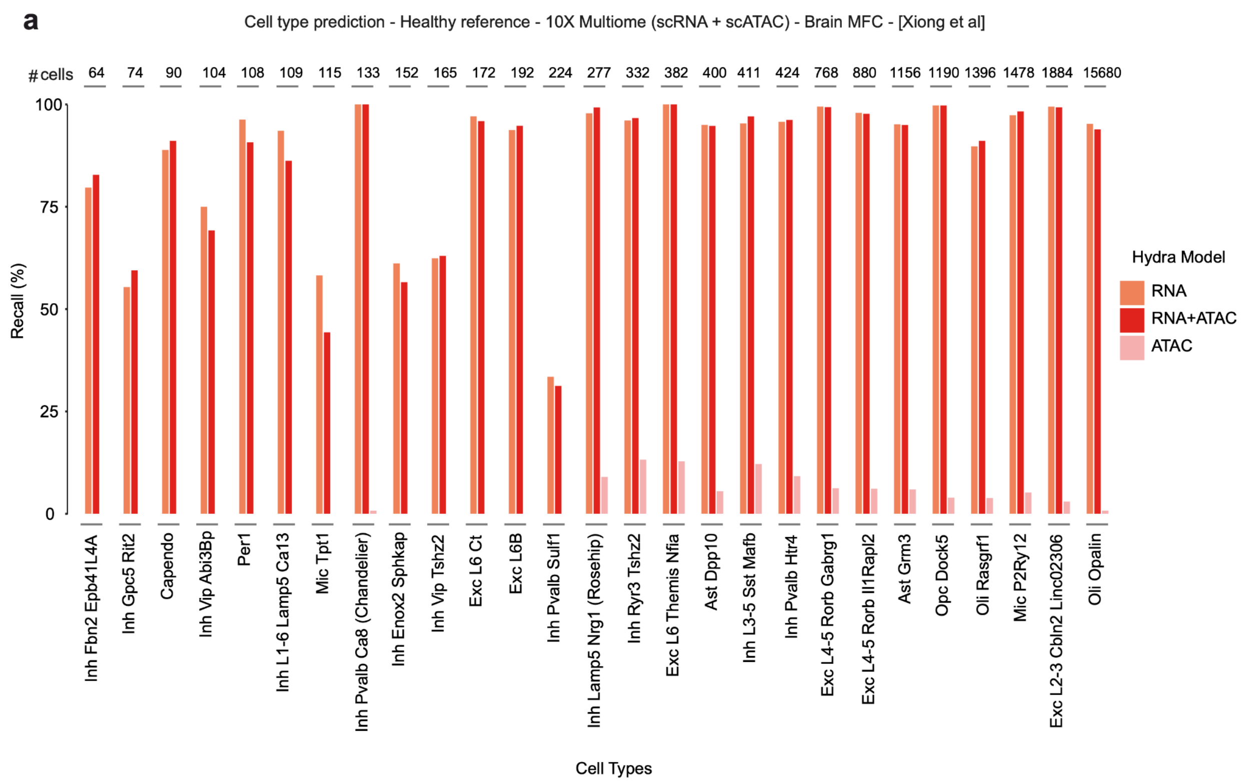

a) Bar plot illustrating cell type prediction performance of Hydra model variants (RNA, RNA+ATAC, ATAC) using healthy medial frontal cortex (MFC) brain dataset. This dataset comprises approximately 28,000 cells across 27 cell types. Prediction performance is displayed for all cell types and cell types are arranged in the order of increasing sample count from left to right.

Minor comments:

1. The equations for the variational autoencoder (VAE) objective are structurally correct but inconsistently formatted. The manuscript alternates between x, x(m), and xi to denote input data without clear definition. Each modality (e.g., RNA, ATAC, ADT) should be explicitly specified in the loss formulation, and the relationship between modality-specific encoders and decoders should be clarified for precision.

Response:

We thank the reviewer for this suggestion. We have now revised the text in the Methods section of the manuscript to explicitly define each input modality. The added text reads as follows:

"For each cell i, let x_i^{RNA} , x_i^{ATAC}, and x_i^{ADT} denote the feature vectors for RNA, ATAC, and ADT modalities respectively where available. Each modality has feature dimensionality d_{RNA}, d_{ATAC}, or d_{ADT}.

For unimodal single-cell transcriptomic data, the the input x_i^{RNA} is first projected through a linear layer with weight matrix $W_{enc}^{RNA} \in R^{hRNA \times d^{RNA}}$ and bias vector $b_{enc}^{RNA} \in R^{hRNA}$, followed by Mish

activation (Misra) and batch normalization (BN) (Ioffe and Szegedy), with a dropout rate of 0.2. This is followed by a second linear transformation to reduce the dimensionality to the latent space z. The reparameterization trick (Kingma and Welling) is applied to sample from z during optimization.

$$\mathbf{h}_i^{\mathrm{RNA}} = \mathrm{Dropout}_{0.2}\left(\mathrm{BN}\left(\mathrm{Mish}(\mathbf{W}_{\mathrm{enc}}^{\mathrm{RNA}}\mathbf{x}_i^{\mathrm{RNA}} + \mathbf{b}_{\mathrm{enc}}^{\mathrm{RNA}})\right)\right)$$

For single-cell multiome data, Hydra can handle up to three modalities (e.g., RNA, ATAC, and ADT) by employing modality-specific encoders and decoders. For a dataset simultaneously profiling all three modalities: $x_i = [x_i^{RNA};x_i^{ATAC};x_i^{ADT}] \in R^{dRNA+dATAC+dADT}$ *, where* $x_i^{RNA} \in R^{dRNA}$ *,* $x_i^{ATAC} \in R^{dATAC}$ *, and* $x_i^{ADT} \in R^{dADT}$ *represent the feature vectors for RNA, ATAC, and ADT modalities respectively. The input is processed by modality-specific encoders* E^{RNA} *,* E^{ATAC}*, and* E^{ADT}*:*

$$\begin{aligned}
\mathbf{h}_i^{\mathrm{RNA}} &= E^{\mathrm{RNA}}(\mathbf{x}_i^{\mathrm{RNA}}) \\
&= \mathrm{Dropout}_{0.2}\left(\mathrm{BN}\left(\mathrm{Mish}(\mathbf{W}_{\mathrm{enc}}^{\mathrm{RNA}}\mathbf{x}_i^{\mathrm{RNA}} + \mathbf{b}_{\mathrm{enc}}^{\mathrm{RNA}})\right)\right) \\
\mathbf{h}_i^{\mathrm{ATAC}} &= E^{\mathrm{ATAC}}(\mathbf{x}_i^{\mathrm{ATAC}}) \\
&= \mathrm{Dropout}_{0.2}\left(\mathrm{BN}\left(\mathrm{Mish}(\mathbf{W}_{\mathrm{enc}}^{\mathrm{ATAC}}\mathbf{x}_i^{\mathrm{ATAC}} + \mathbf{b}_{\mathrm{enc}}^{\mathrm{ATAC}})\right)\right) \\
\mathbf{h}_i^{\mathrm{ADT}} &= E^{\mathrm{ADT}}(\mathbf{x}_i^{\mathrm{ADT}}) \\
&= \mathrm{Dropout}_{0.2}\left(\mathrm{BN}\left(\mathrm{Mish}(\mathbf{W}_{\mathrm{enc}}^{\mathrm{ADT}}\mathbf{x}_i^{\mathrm{ADT}} + \mathbf{b}_{\mathrm{enc}}^{\mathrm{ADT}})\right)\right)
\end{aligned}$$

The modality-specific representations are then concatenated and processed through a fully connected layer to create the shared latent variable z for joint learning. In the decoder, the latent variable z_i *is mapped back to the original feature space. In the decoder, the shared latent variable* z_i *is passed through modality-specific decoders* D^{RNA} *,* D^{ATAC}*, and* D^{ADT}*, where each modality undergoes similar transformations to reconstruct the original inputs.*

$$\begin{aligned}
\hat{\mathbf{x}}_i^{\mathrm{RNA}} &= D^{\mathrm{RNA}}(\mathbf{z}_i) \\
&= \mathrm{Dropout}_{0.2}\left(\mathrm{BN}\left(\mathrm{Mish}(\mathbf{W}_{\mathrm{dec}}^{\mathrm{RNA}}\mathbf{z}_i + \mathbf{b}_{\mathrm{dec}}^{\mathrm{RNA}})\right)\right) \\
\hat{\mathbf{x}}_i^{\mathrm{ATAC}} &= D^{\mathrm{ATAC}}(\mathbf{z}_i) \\
&= \mathrm{Dropout}_{0.2}\left(\mathrm{BN}\left(\mathrm{Mish}(\mathbf{W}_{\mathrm{dec}}^{\mathrm{ATAC}}\mathbf{z}_i + \mathbf{b}_{\mathrm{dec}}^{\mathrm{ATAC}})\right)\right) \\
\hat{\mathbf{x}}_i^{\mathrm{ADT}} &= D^{\mathrm{ADT}}(\mathbf{z}_i) \\
&= \mathrm{Dropout}_{0.2}\left(\mathrm{BN}\left(\mathrm{Mish}(\mathbf{W}_{\mathrm{dec}}^{\mathrm{ADT}}\mathbf{z}_i + \mathbf{b}_{\mathrm{dec}}^{\mathrm{ADT}})\right)\right)
\end{aligned}$$

where $W_{dec}^{RNA} \in R^{dRNA \times dz}$*,* $W_{dec}^{ATAC} \in R^{dATAC \times dz}$ *,* $W_{dec}^{ADT} \in R^{dADT \times dz}$ *and* b_{dec}^{RNA}*,* b_{dec}^{ATAC}*,* b_{dec}^{ADT} *are modality-specific decoder weight matrices and bias vectors. The latent variable* z_i *is also fed into multiple cell type classification heads. The number of classification heads* C_m *is user-configurable, with a default of 25 set empirically."*

2. The loss term LHydra=LVAE+λLCE includes a weighting parameter λ, but its value and tuning procedure are not described. Because this parameter balances reconstruction and classification, even modest changes could alter the results. The manuscript should explain how λ\lambdaλ was chosen-whether by grid search, cross-validation, or fixed heuristics-and whether it was held constant across datasets

Response:

We thank the reviewer for this comment. Hydra extends on the variational autoencoder (VAE) architecture from our previous work (Liu et al., 2023) by incorporating an ensemble framework with multi-task learning for feature selection and cell type prediction. The total loss function combines reconstruction and classification objectives as:

$$L\_total = L\_recon + \lambda\ L\_class$$

We adopt λ = 0.9 as the default value, which was empirically determined in our prior work (Liu et al., 2023) and this value was held constant across all 21 datasets in our experiments. We have now added this in the Methods section of the manuscript.

3. Hydra's ensemble strategy is a central part of the framework, yet the manuscript does not specify how outputs from multiple VAEs are combined. It should be clarified whether ensemble predictions are averaged, majority-voted, or weighted, and how feature rankings are consolidated across ensemble members. A concise mathematical expression or pseudocode would improve clarity.

Response:

We thank the reviewer for this suggestion. We have now added a mathematical expression in the Methods section of the manuscript to clarify how outputs from multiple variational autoencoders are combined for feature ranking. The added text reads as follows:

"*Finally, we obtain a consensus cell type-specific feature ranking by averaging the importance scores across all refined models. Specifically, for a given feature i in cell type c, the consensus importance score is computed as:*

$$\bar{\alpha}_i^c = \frac{1}{M}\sum_{m=1}^{M} \alpha_{m,i}^c$$

where $\alpha^c{}_{m,i}$ *represents the importance score of feature i for cell type c from refined model m, and M is the total number of ensemble models.*"

4. The process for generating synthetic samples from minority cell types is described conceptually but lacks implementation detail. Information about how many synthetic cells are generated per cluster, how latent variables are sampled, and how variance is controlled would make this component more transparent. Including a schematic or short equation would be beneficial.

Response:

We thank the reviewer for this suggestion. We have now clarified the process of generating synthetic cells for minor cell types with a short explanation and mathematical equation in the Methods section of the manuscript. The revised text reads as follows:

"*During the refinement stage, we address class imbalance by generating balanced datasets from the original input single-cell data. We do this by categorizing cell types into major and minor groups based on the median sample size across all cell types. To create a balanced dataset, we randomly down-sample the major cell types and generate additional samples for minor cell types using the original trained model. Specifically, for a minor cell type c with N_c samples (where $N_c < N_{median}$), we generate $N_{syn} = N_{median} - N_c$ synthetic samples, where N_{median} is the median sample size across all cell types. Synthetic cells are generated by sampling from the learned latent distribution of the variational autoencoder:*

$$z_{\text{syn}} \sim \mathcal{N}(\mu_c, \sigma_c^2), \quad x_{\text{syn}} = \text{Decoder}(z_{\text{syn}})$$

where μ_c and $\sigma^2{}_c$ are the mean and variance of the latent representations computed from real cells of type c, and the decoder reconstructs the synthetic sample in the original feature space. For major cell types (where $N_c \geq N_{median}$), we perform random down-sampling to N_{median} samples."

5. Figure 4 uses dataset abbreviations such as "10x-MO," "CITE," and "TEA" without defining them in the caption. These abbreviations should be expanded or defined once in the main figure legend to ensure clarity for readers unfamiliar with the datasets.

Response:

We appreciate the reviewer for this suggestion. We have now revised the text in Figure 4 legend to define the dataset abbreviations to ensure clarity for readers. The updated legend reads as follows:

"*Fig. 4. Evaluation of cell type prediction performance of Hydra using single-cell multiome datasets*

(a) Bar plot with error bars illustrating the 5-fold intra-dataset cell type prediction performance of Hydra against existing methods using the SHARE-seq (Simultaneous High-throughput ATAC and RNA Expression with sequencing) Skin dataset, comprising approximately 25,000 cells. Prediction accuracy for the three smallest and three largest cell types is displayed, followed by the overall performance across all 22 cell types in the dataset (highlighted with a gray background).

(b) Five-time repeated random subsampling intra-dataset cell type prediction accuracy of Hydra against existing methods using the SNARE-seq (Single-Nucleus Chromatin Accessibility and mRNA Expression sequencing) Brain dataset, comprising approximately 8,000 cells. The overall prediction performance across all 23 cell types is indicated with a gray background.

(c) Comparison of five-time repeated random subsampling intra-dataset cell type prediction accuracy of Hydra against other methods using the sciCAR-seq (Single-Cell Combinatorial Indexing chromatin accessibility and RNA sequencing) Kidney dataset, comprising approximately 7,000 cells. Overall performance across all 14 cell types in the dataset is highlighted with a gray background.

(d) Five-time repeated random subsampling intra-dataset cell type prediction performance using the CITE-seq (Cellular Indexing of Transcriptomes and Epitopes by sequencing) PBMC dataset, comprising approximately 6,000 cells. The overall performance across all 26 cell types in the dataset is highlighted with a gray background.

(e) Visualization of the inter-dataset cell type prediction performance of various methods across four distinct single-cell multiome studies: 10X Multiome (10X Chromium Epi Multiome ATAC + Gene Expression), CITE-seq, and TEA-seq (Transcriptome, Epitope, and Accessibility sequencing), spanning 28 train-test splits. The bubble plot ranks methods based on their overall performance."

6. Key training parameters-including optimizer type, learning rate, batch size, number of epochs, ensemble size, and any regularization factors-are not described systematically. These details are essential for reproducibility and fair comparison. A concise summary table of all hyperparameters would be helpful, ideally placed in the Appendix Notes or Methods Appendix to maintain readability in the main text.

Response:

We thank the reviewer for this suggestion. We have now added a table (Table 2) in the revised manuscript summarizing all key training parameters used in the feature ranking and cell type annotation modules of Hydra:

Parameter	Default value
Feature Ranking Module (VAE)	
Learning rate	0.02
Batch size	512
Epochs (Initial training)	40
Epochs (Model refinement)	30-50
Epochs (Early stopping)	10
Ensemble size	25
Latent dimension (z)	100
Hidden layer (RNA)	185
Hidden layer (ADT)	30
Hidden layer (ATAC)	185
Cell Type Annotation Module	
Learning rate	0.01
Batch size	64
Epochs	5
Hidden layer dimension	128
Ensemble size	25

7. Minor grammatical and stylistic edits are recommended for precision and tone (e.g., "rare population" → "rare populations"; "a deep model" → "deep models"). The writing could also be slightly moderated-phrases such as "significantly outperforms" should be reserved for results supported by explicit statistical testing.

Response:

We appreciate the reviewer for this comment. We have revised the manuscript to incorporate the suggested edits. We have also replaced instances of "significantly outperforms" with simply "outperforms", where formal statistical testing was not performed. We thank the reviewer again for these suggestions.

Reviewer #2:

The authors present Hydra, a new deep learning framework for analyzing single-cell omics data. The work aims to tackle two significant challenges: the difficulty of annotating rare cell types and the "black box" nature of many deep learning models. The method uses an ensemble of variational autoencoders (VAEs) to learn the data's structure and then generates synthetic data for rare cell populations to create balanced datasets for training. It incorporates a technique called Integrated Gradients to identify the key molecular features defining each cell type, which makes the model interpretable. Finally, an ensemble of simple neural networks is trained on these top ranking features. The authors tested Hydra on 21 different datasets, showing it

generally performs as well as or better than other state-of-the-art tools, with a clear advantage in identifying rare cells. The study is rounded out with a case study on Alzheimer's disease data, demonstrating the tool's robustness for complex biological questions. The authors have performed a comprehensive and rigorous evaluation of Hydra. Using 21 distinct datasets that span multiple technologies, modalities, and tissues provides strong evidence for the method's generalizability and robustness. The paper is well-written and the figures clearly illustrate the framework.

Response: We thank the reviewer for the thoughtful assessment and appreciate their recognition of Hydra's ability to identify rare cell types and its robust evaluation across diverse datasets. We address the points raised below.

1. The comparison baselines for the scRNA-seq annotation task could be updated to strengthen the claims of superiority. Among the baselines, the most recent method, scClassify, was published in 2020. The field is fast-moving, and including comparisons against more recent, state-of-the-art annotation methods would make the performance claims more persuasive.

Response:

We have now updated our benchmarking to include more recent methods. Specifically, we replaced scVI with scANVI (Xu et al., 2021), a semi-supervised extension that builds upon scVI and leverages labeled data for supervised cell type prediction. We have also included CellTypist (Domínguez et al., 2022), a widely used automated cell type annotation tool that utilizes logistic regression to robustly predict cell types in query datasets. Additionally, we have included a more recent ensemble deep learning framework called scSorterDL (Bai et al., 2025) that integrates deep neural networks, linear discriminant analysis and swarm learning for cell type identification.

As shown in our analyses below, across 13 single-cell transcriptomic datasets spanning diverse tissues and studies, we find that overall Hydra achieved the highest balanced accuracy outperforming all other state-of-the-art tools in both intra-dataset and inter-dataset cell type identification tasks (Fig. 3, Appendix Fig. S5-S7). We have now included these results and figures in the revised manuscript.

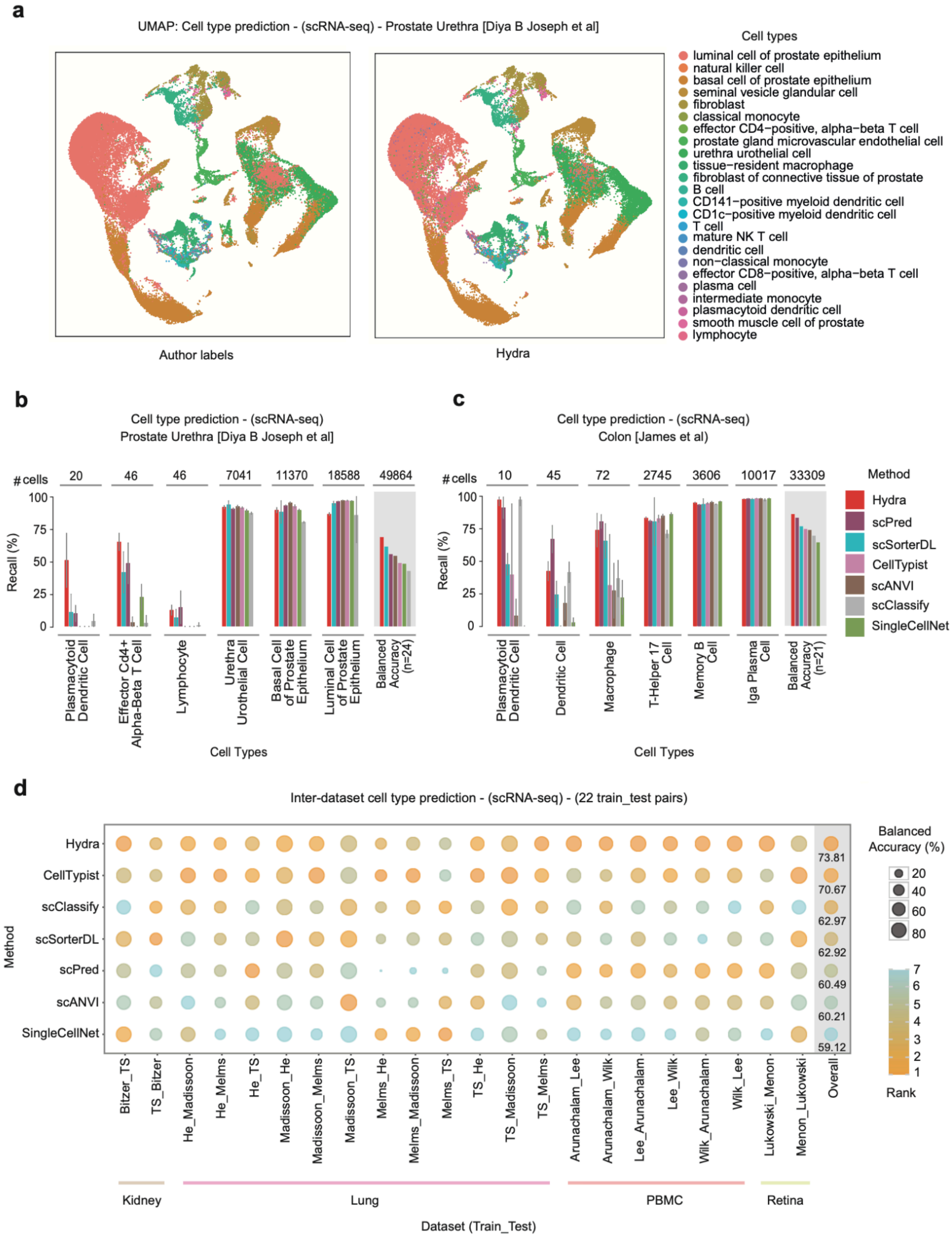

(a) Uniform Manifold Approximation and Projection (UMAP) plots for the single-cell transcriptomic prostate dataset, comprising 24 cell types and approximately 49,000 cells. The

left panel shows the original labels as provided by the authors of the study, while the right panel shows the cell types predicted by Hydra.

(b) Bar plot with error bars illustrating the five-time repeated random subsampling intra-dataset cell type prediction performance of various methods using the single-cell transcriptomic prostate dataset. The three smallest and three largest cell types are displayed, arranged in order of increasing sample count from left to right, followed by the overall performance across all cell types in the dataset (highlighted with a gray background).

(c) Bar plot with error bars representing the five-time repeated random subsampling intra-dataset cell type prediction performance of different methods using a single-cell transcriptomic colon dataset comprising approximately 33,000 cells. The three smallest and three largest cell types are displayed, arranged in order of increasing sample count from left to right, followed by the overall performance across all 21 cell types in the dataset (highlighted with a gray background).

(d) Bubble plot illustrating the inter-dataset cell type prediction performance of various methods across four tissues (Kidney, Lung, PBMC, and Retina) and 22 train-test pairs. Methods are ranked based on their overall balanced accuracy across all train-test pairs.

2. The use of deep ensemble models raises significant questions about computational efficiency. The manuscript lacks an analysis of the computational resources (CPU/GPU time, memory) required to train and run Hydra, especially in comparison to the benchmarked methods. This is a critical practical limitation for its adoption by the research community.

Response:

We thank the reviewer for this valuable suggestion. To address this, we compared the computational cost of running Hydra against all baseline methods included in our benchmarking analysis across varying dataset sizes for both single-cell transcriptomic (Madissoon et al., 2019) and single-cell multiome data (Ma et al., 2020, Stephenson et al., 2021). Our results show that Hydra completed training in under 10 minutes across all evaluated datasets and cell counts (Fig. 6). We found that Hydra had a higher GPU consumption compared to other methods, which is attributed to its ensemble framework for VAE training and feature selection. Nevertheless, these findings demonstrate that Hydra is computationally feasible for real-world applications in single-cell omics analysis. We have now included these results in the revised manuscript.

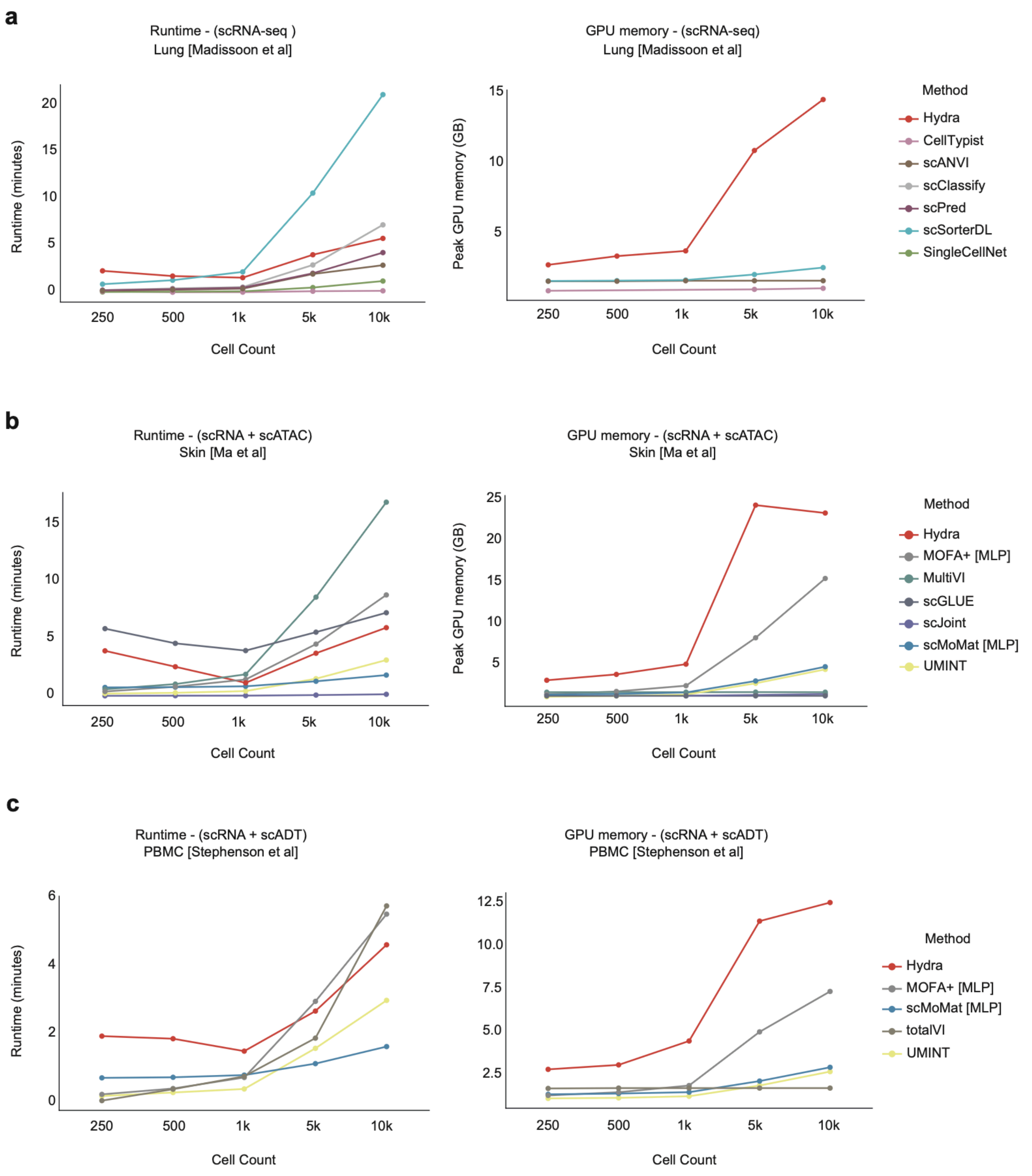

(a-c) Line plots showing runtime (left panel) and GPU consumption (right panel) of different methods across varying cell counts (250, 500, 1000, 5000, 10000). Evaluations were performed on single-cell transcriptomic data (a), joint profiling of single-cell transcriptomic and chromatin accessibility data (b) and joint profiling of single-cell transcriptomic and surface protein data (c)

3. A cornerstone of the method is the VAE-based generation of synthetic cells to augment rare populations. However, the quality of these synthetic cells is not evaluated. It is essential to demonstrate that the generated data faithfully recapitulates the biological identity of the rare cells without introducing artifacts.

Response:

We thank the reviewer for this suggestion. To address this, we evaluated the quality of synthetic cells generated by our VAE-based augmentation approach. We generated 100 synthetic cells for each of the 20 cell types and performed the following two complementary analyses:

1) Spatial co-localization: To assess whether our method generates synthetic cells in a cell type-specific manner, we visualized both real and synthetic cells on UMAP. We found that synthetic cells localized in the same regions as their corresponding real cells, indicating that the model learned appropriate cell type identities.

2) Preservation of gene co-expression structure: To assess whether synthetic cells preserve the biological relationships between cell types, we compared the correlation structure of gene expression profiles across all 20 cell types. Specifically, we computed pairwise Pearson correlations between expression profiles for both real and synthetic cells. The high positive correlation demonstrates that synthetic cells preserve the coordinated expression patterns and inter-cellular relationships observed in real cells.

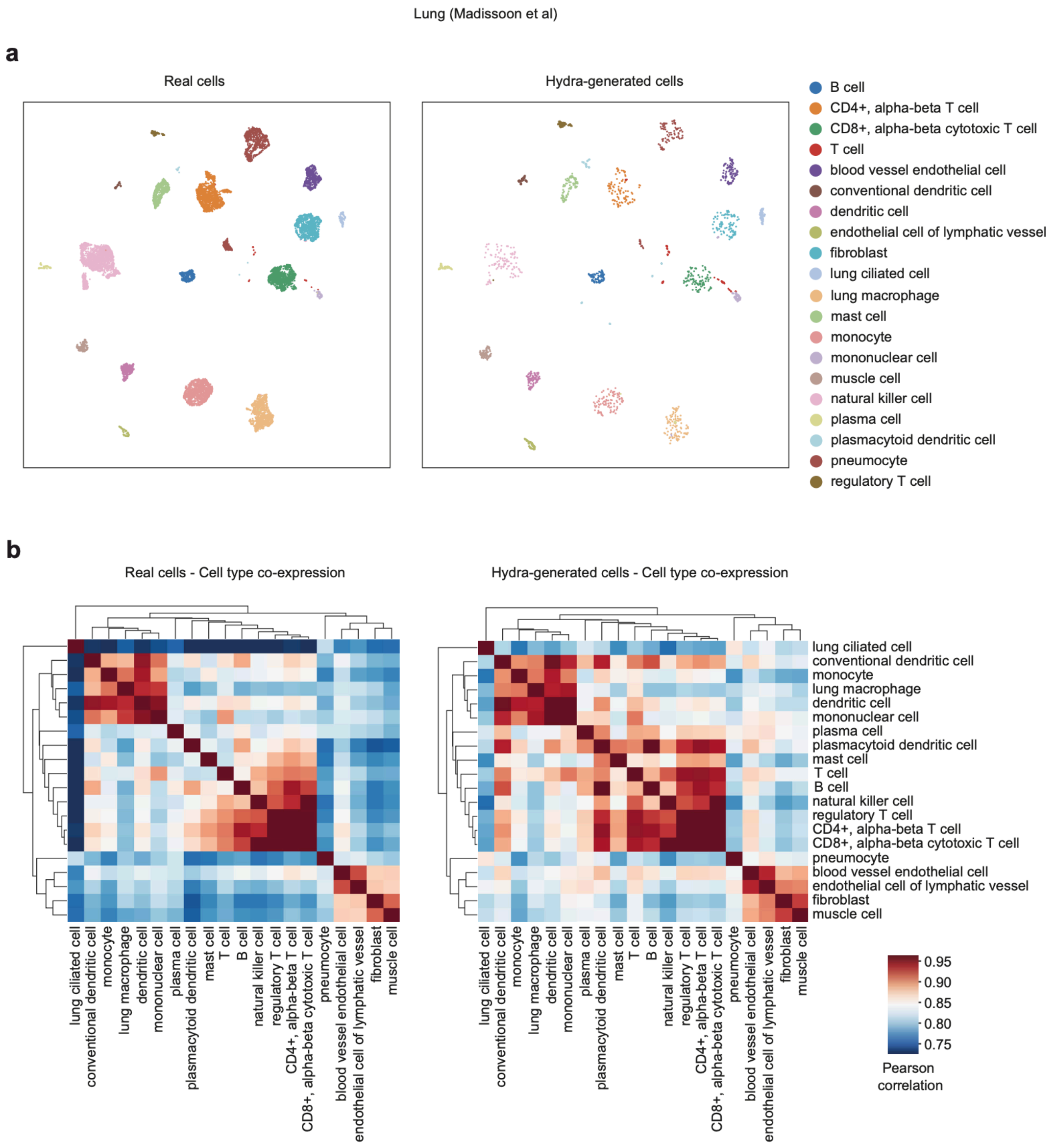

(a) Uniform Manifold Approximation and Projection (UMAP) visualization of real cells (left panel) and Hydra-generated synthetic cells (right panel) using the lung dataset for all cell types.

(b) Heatmaps showing pairwise Pearson correlation coefficients between gene expression profiles across all cell types for real cells (left panel) and Hydra-generated synthetic cells (right panel).

4. The tool is set up for supervised annotation, meaning it can only find cell types it was trained on. A major real-world challenge is the presence of novel or unexpected cell types in a query sample. The manuscript does not discuss how Hydra would handle such cases or address this limitation.

Response: We thank the reviewer for highlighting this important point. We acknowledge that handling novel or unknown cell types is a major challenge in single-cell omics analysis. As Hydra is currently designed as a supervised framework, it is restricted to predicting cell types present in the training reference. We have updated the Discussion section of the manuscript to acknowledge this limitation and highlight the need for discovering novel cell populations in the future work. The added paragraph reads as follows:

"Although Hydra demonstrates robust performance across diverse datasets and biological conditions, we acknowledge limitations inherent to its supervised framework. First, the model is restricted to predicting cell types present in the training reference and does not currently support the discovery of novel or unannotated cell populations in query datasets. Second, as with most supervised tools, Hydra relies on the quality of input cell type labels in the reference dataset and significant label noise could potentially affect its prediction performance. Future extensions of the framework would therefore focus on integrating approaches to detect unseen cell populations and conduct systematic sensitivity analyses to address label noise."

Minor issues:

1. The authors should point out that it's a "cell type classification head" in Figure 1. The current description in Figure 1 and in the session "Overview of Hydra framework" is not clear for the classification head.

Response:

We thank the reviewer for this suggestion. We have now revised Figure 1 and the "Overview of Hydra framework" section in the manuscript to explicitly describe these as "cell type classification heads."

Specifically, we now state the following in the main text:

"*First, the VAE architecture is jointly trained with an ensemble of cell type classification heads (Fig. 1a).*"

We have also updated the Figure 1a legend to read:

"*Graphical illustration of key architectural components in the Hydra framework, including the variational autoencoder (VAE) and the ensemble of cell type classification heads.*"

2. What are the differences between ensemble models 1 to M?

Response:

We thank the reviewer for requesting this clarification. The ensemble models 1 to M refer to the distinct cell type classification heads (C_1, C_2, ..., C.) illustrated in Figure 1a. Specifically, M is a user-configurable parameter that determines the ensemble size of the Hydra framework. A default value of 25 is established empirically in our stability analysis (Fig. 2a-b, Appendix Fig 1a-b).

3. Since Integrated Gradients (IG) is a key concept in the feature ranking module, the authors should introduce this concept briefly in the Methods section.

Response:

We thank the reviewer for this valuable suggestion. We have now added the following introduction to Integrated Gradients in the methodology section of the manuscript. The revised text reads as follows:

"Integrated Gradients is an axiomatic feature attribution method designed to explain predictions of deep neural networks by quantifying the contribution of each input feature to the output. Specifically, it computes feature attributions by accumulating gradients along a straightline path from a baseline input to the actual input. This approach satisfies two fundamental properties - (i) sensitivity, which assigns non-zero importance to features that influence predictions, and (ii) implementation invariance, which ensures consistent attributions for functionally equivalent networks (Sundararajan et al., 2017, Ancona et al., 2017). Mathematically, for an input x_i and baseline x_i', the Integrated Gradients attribution for feature k is defined as:

$$\mathrm{IG}_k(\mathbf{x}_i) = (x_{i,k} - x'_{i,k}) \int_{\alpha=0}^{1} \frac{\partial F(\mathbf{x}'_i + \alpha \times (\mathbf{x}_i - \mathbf{x}'_i))}{\partial x_{i,k}} d\alpha$$

where α is the interpolation parameter ranging from 0 to 1, F represents the output of the cell type classification head for a given cell type, and $x_{i,k}$ denotes the k-th feature of cell i. Using Riemann summation, the integral is approximated numerically over discrete steps along the interpolation path. To improve computational efficiency, we adopt a batch attribution approach on all cells belonging to a given cell type. For a given cell type c and model m, the feature importance scores $\theta_c^m \in R^d$ is computed by averaging the absolute values of the Integrated Gradients attributions over all cells of type c:

$$\theta^m_{c,k} = \frac{1}{N_c} \sum_{i \in \mathcal{C}_c} |\mathrm{IG}_k(f_m, \mathbf{x}_i, \mathrm{target} = c)|$$

where N_c is the total number of cells of type c, C_c = {i : cell i has type c} is the set of cell indices with type c, $\theta_{c,k}^m$ is the importance score for feature k in cell type c from model m, and f_m is the model comprising the encoder and classification head C_m from the refined model m. The features are then ranked considering the biological directionality. Finally, we obtain a consensus

cell type-specific feature ranking by averaging the importance scores across all refined models. Specifically, for a given feature k in cell type c, the consensus importance score is computed as:

$$\bar{\theta}_{c,k} = \frac{1}{M} \sum_{m=1}^{M} \theta_{c,k}^{m}$$

where $\theta_{c,k}^{m}$ represents the importance score of feature k for cell type c from refined model m, and M is the total number of ensemble models."

4. A thorough proofread is recommended to catch minor typos. For example, there is a typo in "... a single model where no ensemble is not used."

Response:

We thank the reviewer for pointing out this typo. We have corrected the phrase "where no ensemble is not used" to "*where no ensemble is used*" in the manuscript. Additionally, we have conducted a proofread of the manuscript to address other minor typographical inconsistencies.

Reviewer #3:

Hydra presents an ensemble VAE framework for interpretable single-cell feature selection and annotation. The method performs well empirically, especially on rare cell types, but the manuscript suffers from conceptual and methodological ambiguities which have to be clarified.

We sincerely thank the reviewer for their thoughtful assessment of our manuscript and valuable suggestions. Please find our responses below.

1. Sampling of Rare Populations

The approach to sampling rare cell populations for intra-dataset evaluation is not entirely clear. If it oversampled rare cell types, does this risk overfitting to rare cell types? Have the authors compared to across dataset and study evaluations?

Response:

We thank the reviewer for this comment. For intra-dataset evaluations, rare cell populations were balanced only within the training procedure to mitigate class imbalance, while performance was always assessed on held-out data stratified by the original cell type proportions, thereby reducing the risk of overfitting to rare populations. Importantly, we also performed extensive inter-dataset evaluations, in which models trained on one dataset were applied to independent datasets (e.g., batch, study). These analyses, described in the Results section (Fig. 3–4), demonstrate that Hydra generalizes well across datasets, indicating that the observed performance gains are not driven by overfitting to rare cell populations.

2. Rank Aggregation via IG Method

The choice of the IG (Integrated Gradients) method for rank aggregation is intriguing. Could the authors elaborate on why this particular method was selected

Response:

We thank the reviewer for their comment. We selected Integrated Gradients (Sundararajan et al., 2017) as the default feature attribution approach based on its higher stability in the selected cell type-specific features. Specifically, we compared Integrated Gradients against three alternative attribution techniques, mainly, Saliency (Simonyan et al., 2013), GradientSHAP (Lundberg et al., 2017), and DeepLIFT (Shrikumar et al., 2017). As shown in Appendix Fig. S2, all four approaches yield comparable cell type prediction performance. However, Integrated Gradients consistently demonstrates higher stability and lower variability in the ranked cell type-specific features across datasets and cell types. Feature stability is critical for Hydra's objective of identifying reproducible and interpretable cell type markers, particularly for rare populations where sampling variability is high. For these reasons, we adopt Integrated Gradients as the default attribution method in Hydra. We have now clarified this rationale in the Results section of the revised manuscript.

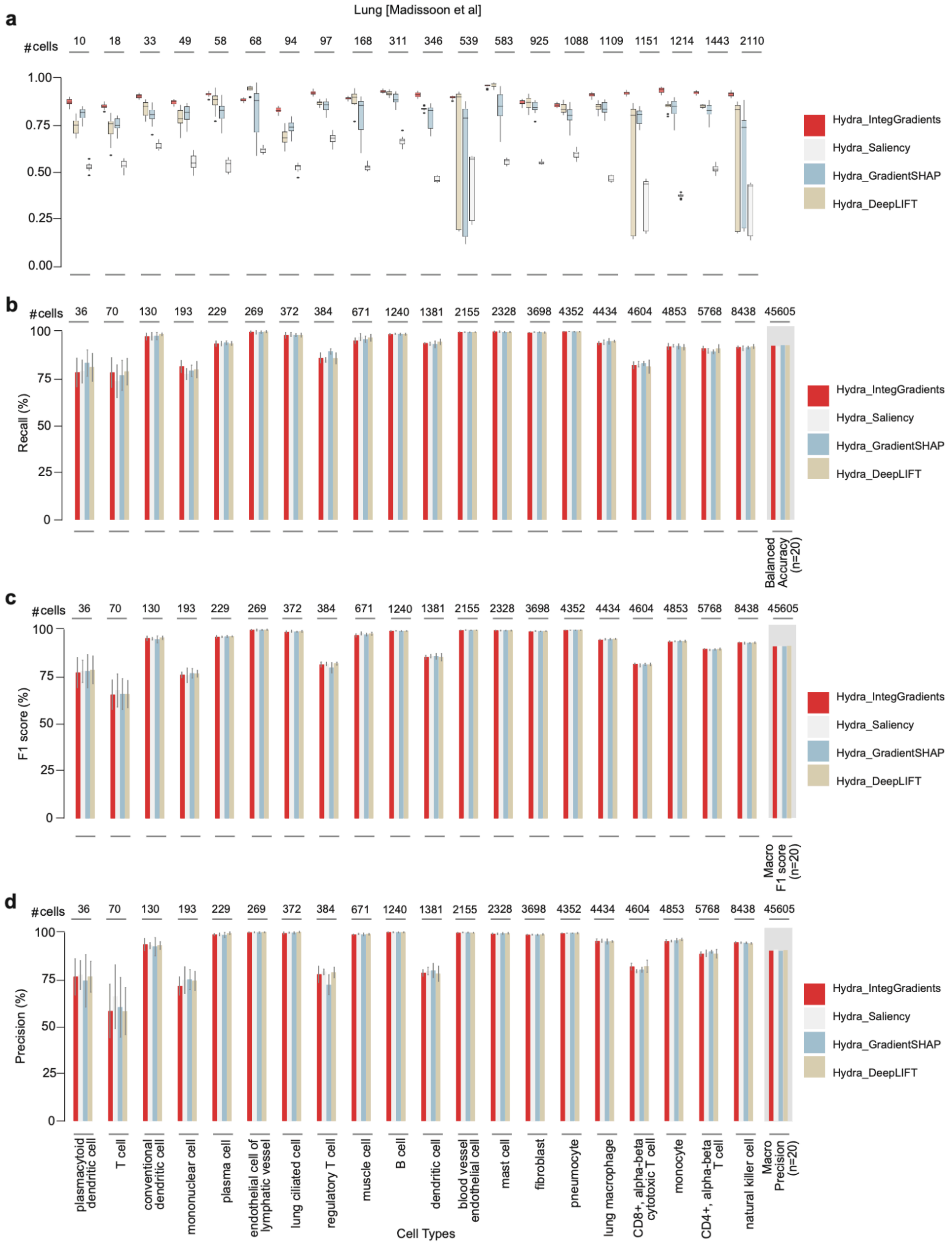

(a) Stability of features selected by different attribution approaches in Hydra (Integrated

Gradients, Saliency, GradientSHAP, and DeepLIFT) for all cell types in the lung dataset (n=20), quantified by Pearson correlation coefficients. Cell types are arranged in the order of increasing sample count from left to right.

(b-d) Cell type prediction performance of different attribution approaches in Hydra evaluated using recall (b), F1 score (c) and precision (d). Cell types are arranged in the order of increasing sample count from left to right, followed by the overall performance (highlighted with a gray background)

3. Multi-Omics Data Processing

For multi-omics data, please clarify how the tool handles multiple modalities. Are modalities concatenated or linked through shared features? Does the model employ separate encoder-decoder structures for each modality, or a shared architecture?

Response:

We thank the reviewer for requesting this clarification. For single-cell multi-omics data, Hydra employs modality-specific encoder layers before being projected into a shared latent space for joint learning. Similarly, the decoder utilizes modality-specific layers for reconstruction. We have expanded the Methods section of the manuscript to provide details on this. The text reads as follows:

"For single-cell multiome data, Hydra can handle up to three modalities (e.g., RNA, ATAC, and ADT) by employing modality-specific encoders and decoders. For a dataset simultaneously profiling all three modalities: $x_i = [x_i^{RNA}; x_i^{ATAC}; x_i^{ADT}] \in R^{dRNA+dATAC+dADT}$ *, where* $x_i^{RNA} \in R^{dRNA}$ *,* $x_i^{ATAC} \in R^{dATAC}$ *, and* $x_i^{ADT} \in R^{dADT}$ *represent the feature vectors for RNA, ATAC, and ADT modalities respectively. The input is processed by modality-specific encoders* E^{RNA} *,* E^{ATAC}*, and* E^{ADT}*:*

$$
\begin{aligned}
\mathbf{h}_i^{\mathrm{RNA}} &= E^{\mathrm{RNA}}(\mathbf{x}_i^{\mathrm{RNA}}) \\
&= \mathrm{Dropout}_{0.2}\left(\mathrm{BN}\left(\mathrm{Mish}(\mathbf{W}_{\mathrm{enc}}^{\mathrm{RNA}}\mathbf{x}_i^{\mathrm{RNA}} + \mathbf{b}_{\mathrm{enc}}^{\mathrm{RNA}})\right)\right) \\
\mathbf{h}_i^{\mathrm{ATAC}} &= E^{\mathrm{ATAC}}(\mathbf{x}_i^{\mathrm{ATAC}}) \\
&= \mathrm{Dropout}_{0.2}\left(\mathrm{BN}\left(\mathrm{Mish}(\mathbf{W}_{\mathrm{enc}}^{\mathrm{ATAC}}\mathbf{x}_i^{\mathrm{ATAC}} + \mathbf{b}_{\mathrm{enc}}^{\mathrm{ATAC}})\right)\right) \\
\mathbf{h}_i^{\mathrm{ADT}} &= E^{\mathrm{ADT}}(\mathbf{x}_i^{\mathrm{ADT}}) \\
&= \mathrm{Dropout}_{0.2}\left(\mathrm{BN}\left(\mathrm{Mish}(\mathbf{W}_{\mathrm{enc}}^{\mathrm{ADT}}\mathbf{x}_i^{\mathrm{ADT}} + \mathbf{b}_{\mathrm{enc}}^{\mathrm{ADT}})\right)\right)
\end{aligned}
$$

The modality-specific representations are then concatenated and processed through a fully connected layer to create the shared latent variable z for joint learning. In the decoder, the latent variable z_i *is mapped back to the original feature space. In the decoder, the shared latent variable* z_i *is passed through modality-specific decoders* D^{RNA} *,* D^{ATAC}*, and* D^{ADT}*, where each modality undergoes similar transformations to reconstruct the original inputs.*

$$
\begin{aligned}
\hat{\mathbf{x}}_i^{\mathrm{RNA}} &= D^{\mathrm{RNA}}(\mathbf{z}_i) \\
&= \mathrm{Dropout}_{0.2}\left(\mathrm{BN}\left(\mathrm{Mish}(\mathbf{W}_{\mathrm{dec}}^{\mathrm{RNA}}\mathbf{z}_i + \mathbf{b}_{\mathrm{dec}}^{\mathrm{RNA}})\right)\right) \\
\hat{\mathbf{x}}_i^{\mathrm{ATAC}} &= D^{\mathrm{ATAC}}(\mathbf{z}_i) \\
&= \mathrm{Dropout}_{0.2}\left(\mathrm{BN}\left(\mathrm{Mish}(\mathbf{W}_{\mathrm{dec}}^{\mathrm{ATAC}}\mathbf{z}_i + \mathbf{b}_{\mathrm{dec}}^{\mathrm{ATAC}})\right)\right) \\
\hat{\mathbf{x}}_i^{\mathrm{ADT}} &= D^{\mathrm{ADT}}(\mathbf{z}_i) \\
&= \mathrm{Dropout}_{0.2}\left(\mathrm{BN}\left(\mathrm{Mish}(\mathbf{W}_{\mathrm{dec}}^{\mathrm{ADT}}\mathbf{z}_i + \mathbf{b}_{\mathrm{dec}}^{\mathrm{ADT}})\right)\right)
\end{aligned}
$$

where $W_{dec}{}^{RNA} \in R^{dRNA \times dz}$, $W_{dec}{}^{ATAC} \in R^{dATAC \times dz}$, $W_{dec}{}^{ADT} \in R^{dADT \times dz}$ and $b_{dec}{}^{RNA}$, $b_{dec}{}^{ATAC}$, $b_{dec}{}^{ADT}$ are modality-specific decoder weight matrices and bias vectors. The latent variable z_i is also fed into multiple cell type classification heads. The number of classification heads C_m is user-configurable, with a default of 25 set empirically."

4. Interpretability and Top-Ranked Features

The interpretability aspect seems primarily based on identifying top-ranked features. Interpretability for deep learning models based on purely feature ranking seems an over-claim: the challenging of interpret deep learning models with existing techniques are not about feature selection (SHAP-value, etc, can be used seamlessly) but how they interact to produce the final predictions.

Response:

We thank the reviewer for this important comment. We acknowledge that post-hoc feature attribution approaches such as Integrated Gradients identify the most important features driving predictions rather than complex interaction between features to produce final outputs. We have now revised the manuscript to clarify the distinction in the Discussion section of the manuscript. Specifically, we note that attributions provide a ranked list of biologically meaningful cell type-specific features rather than complete mechanistic explanations. The revised text reads as follows:

"*Hydra attempts to address the challenge of interpretability of traditional deep learning models through post-hoc feature attribution via Integrated Gradients (Sundararajan et al., 2017). We note that with post-hoc interpretability, additional analyses are performed on trained neural networks to explain the model. This differs from intrinsic interpretability, where prior knowledge or biologically interpretable designs are directly incorporated into the model architecture (Lipton et al., 2018, Murdoch et al., 2019, Wagle et al., 2024). Further, post-hoc feature attribution techniques identify the most important features influencing predictions and not how features interact within the network to produce final outputs. Thus, the attribution approach employed in Hydra provides a ranked list of biologically meaningful cell type-specific features rather than complete mechanistic explanations of the model's internal computations. Nevertheless, these features retain practical utility for distinguishing cell populations and the ensemble deep learning approach further improves feature stability.*"

5. Comparison with Recently Proposed Methods

Several recently proposed methods, such as the following, may serve as valuable benchmarks for comparison:

o https://www.biorxiv.org/content/10.1101/2025.05.06.652331v1.abstract

ohttps://assets-eu.researchsquare.com/files/rs-5754185/v1_covered_91fcffb4-96c9-430d-8c9d-ea32682e66cf.pdf

Response:

We thank the reviewer for this valuable suggestion. To the best of our knowledge, UNICORN (Liu et al., 2025) is a sequence-to-expression prediction model that predicts gene expression levels from DNA sequences using foundation model embeddings and not specifically designed for cell type annotation or cell type-specific feature identification from single-cell omics data, which is the focus of our current study. While UniCell (Hu et al., 2025) is a hierarchical cell type identification method, it leverages cell ontology structure for predicting cell types in single-cell transcriptomic data. This requires standardized ontology labels for hundreds of cell types in our benchmarked datasets and manually assigning such ontology IDs to author labels is currently not feasible and would introduce additional assumptions beyond the scope of this evaluation.

Nevertheless, to strengthen the benchmarking and better reflect the current state of the field, we have expanded our comparisons to include more recent state-of-the-art methods. For single-cell transcriptomic annotation tasks, we have now included scANVI (Xu et al., 2021), CellTypist (Domínguez et al., 2022), and scSorterDL (Bai et al., 2025). For single-cell multiome cell type prediction, we additionally included scGLUE (Cao et al., 2022), scJoint (Lin et al., 2022), totalVI (Gayoso et al., 2021), and MultiVI (Ashuach et al., 2023). We found that Hydra consistently achieved high balanced accuracy, outperforming all 13 methods in both intra-dataset and inter-dataset cell type identification scenarios. All results and figures have been updated in the revised manuscript (Fig. 3-5, Appendix Fig. S5-S14). We thank the reviewer again for encouraging broader comparison with recent approaches.

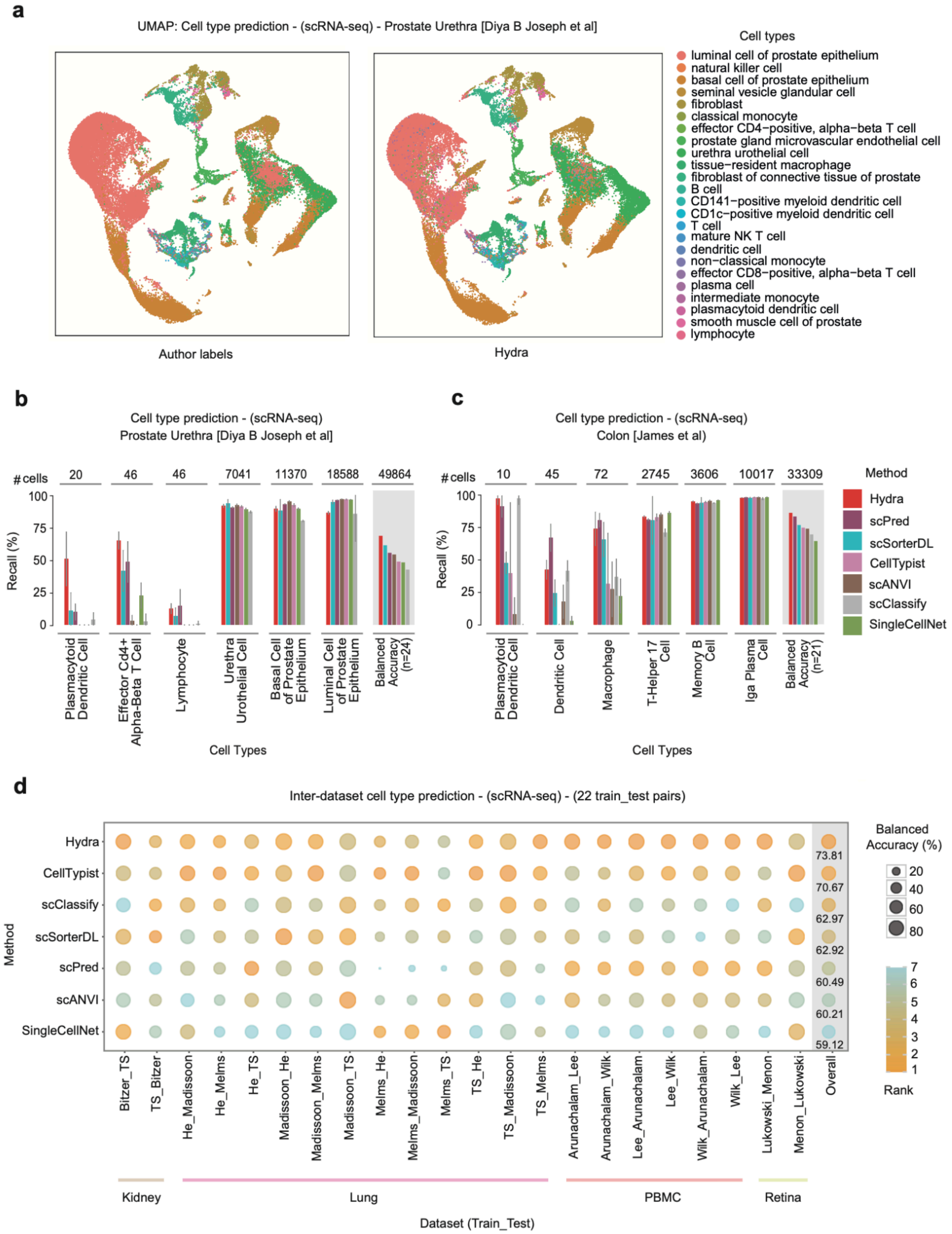

(a) Uniform Manifold Approximation and Projection (UMAP) plots for the single-cell transcriptomic prostate dataset, comprising 24 cell types and approximately 49,000 cells. The

left panel shows the original labels as provided by the authors of the study, while the right panel shows the cell types predicted by Hydra.

(b) Bar plot with error bars illustrating the five-time repeated random subsampling intra-dataset cell type prediction performance of various methods using the single-cell transcriptomic prostate dataset. The three smallest and three largest cell types are displayed, arranged in order of increasing sample count from left to right, followed by the overall performance across all cell types in the dataset (highlighted with a gray background).

(c) Bar plot with error bars representing the five-time repeated random subsampling intra-dataset cell type prediction performance of different methods using a single-cell transcriptomic colon dataset comprising approximately 33,000 cells. The three smallest and three largest cell types are displayed, arranged in order of increasing sample count from left to right, followed by the overall performance across all 21 cell types in the dataset (highlighted with a gray background).

(d) Bubble plot illustrating the inter-dataset cell type prediction performance of various methods across four tissues (Kidney, Lung, PBMC, and Retina) and 22 train-test pairs. Methods are ranked based on their overall balanced accuracy across all train-test pairs.

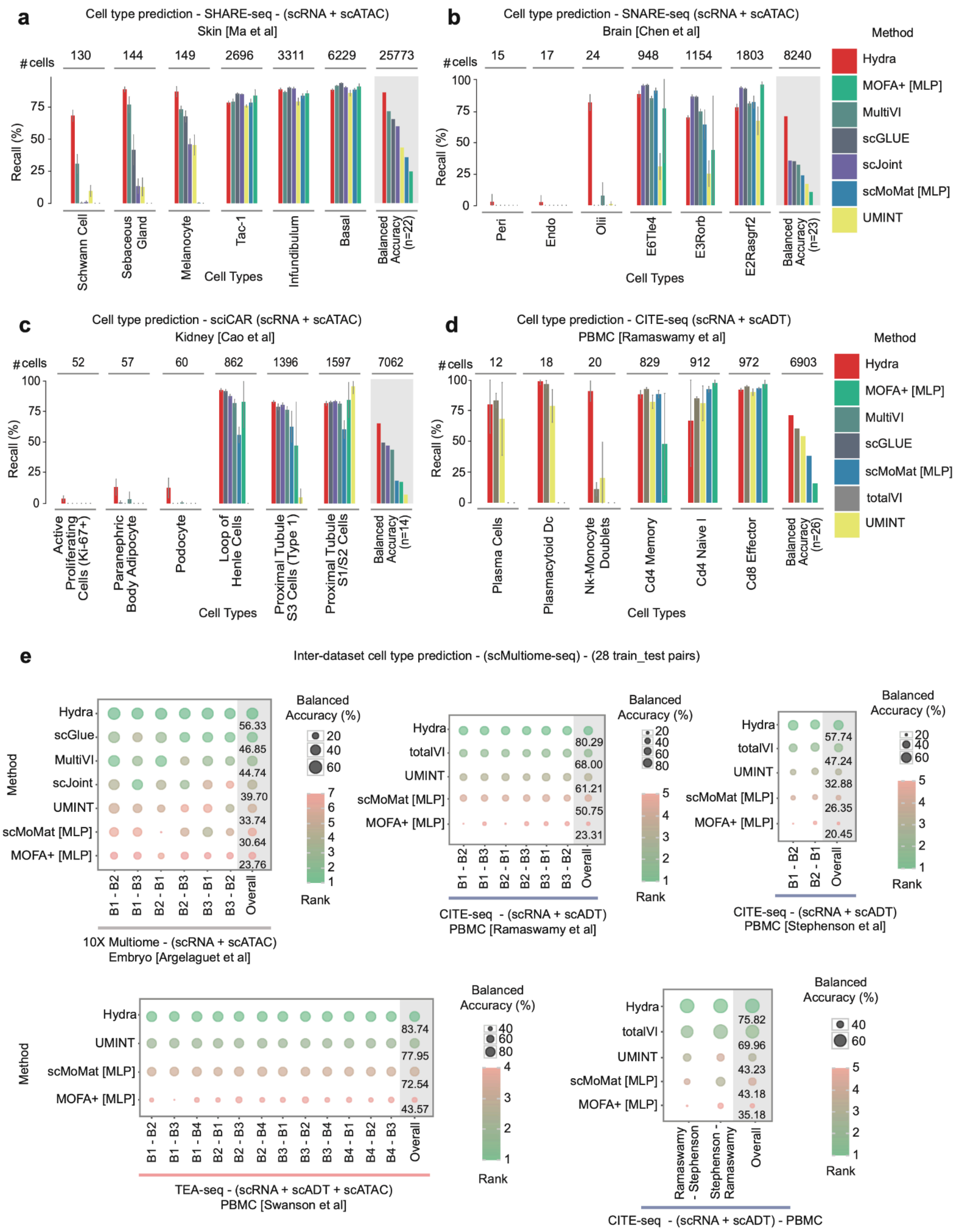
a
Cell type prediction - SHARE-seq - (scRNA + scATAC)
Skin [Ma et al]
cells
130
144
149
2696
3311
6229
25773
Recall (%)
Schwann Cell
Sebaceous Gland
Melanocyte
Tac-1
Infundibulum
Basal
Balanced Accuracy (n=22)
Cell Types
b
Cell type prediction - SNARE-seq (scRNA + scATAC)
Brain [Chen et al]
cells
15
17
24
948
1154
1803
8240
Recall (%)
Peri
Endo
Oli
E6Tle4
E3Rorb
E2Rasgrf2
Balanced Accuracy (n=23)
Cell Types
Method
Hydra
MOFA+ [MLP]
MultiVI
scGLUE
scJoint
scMoMat [MLP]
UMINT
c
Cell type prediction - sciCAR (scRNA + scATAC)
Kidney [Cao et al]
cells
52
57
60
862
1396
1597
7062
Recall (%)
Active Proliferating Cells (Ki-67+)
Paranephric Body Adipocyte
Podocyte
Loop of Henle Cells
Proximal Tubule S3 Cells (Type 1)
Proximal Tubule S1/S2 Cells
Balanced Accuracy (n=14)
Cell Types
d
Cell type prediction - CITE-seq (scRNA + scADT)
PBMC [Ramaswamy et al]
cells
12
18
20
829
912
972
6903
Recall (%)
Plasma Cells
Plasmacytoid Dc
Nk-Monocyte Doublets
Cd4 Memory
Cd4 Naive I
Cd8 Effector
Balanced Accuracy (n=26)
Cell Types
Method
Hydra
MOFA+ [MLP]
MultiVI
scGLUE
scMoMat [MLP]
totalVI
UMINT
e
Inter-dataset cell type prediction - (scMultiome-seq) - (28 train_test pairs)
Method
Hydra 56.33
scGlue 46.85
MultiVI 44.74
scJoint 39.70
UMINT 33.74
scMoMat [MLP] 30.64
MOFA+ [MLP] 23.76
B1 - B2
B1 - B3
B2 - B1
B2 - B3
B3 - B1
B3 - B2
Overall
Balanced Accuracy (%)
Rank
10X Multiome - (scRNA + scATAC)
Embryo [Argelaguet et al]
Hydra 80.29
totalVI 68.00
UMINT 61.21
scMoMat [MLP] 50.75
MOFA+ [MLP] 23.31
CITE-seq - (scRNA + scADT)
PBMC [Ramaswamy et al]
Hydra 57.74
totalVI 47.24
UMINT 32.88
scMoMat [MLP] 26.35
MOFA+ [MLP] 20.45
CITE-seq - (scRNA + scADT)
PBMC [Stephenson et al]
Hydra 83.74
UMINT 77.95
scMoMat [MLP] 72.54
MOFA+ [MLP] 43.57
B1 - B2
B1 - B3
B1 - B4
B2 - B1
B2 - B3
B2 - B4
B3 - B1
B3 - B2
B3 - B4
B4 - B1
B4 - B2
B4 - B3
Overall
TEA-seq - (scRNA + scADT + scATAC)
PBMC [Swanson et al]
Hydra 75.82
totalVI 69.96
UMINT 43.23
scMoMat [MLP] 43.18
MOFA+ [MLP] 35.18
Ramaswamy - Stephenson
Stephenson - Ramaswamy
Overall
CITE-seq - (scRNA + scADT) - PBMC

(a) Bar plot with error bars illustrating the 5-fold intra-dataset cell type prediction performance of Hydra against existing methods using the SHARE-seq (Simultaneous High-throughput ATAC and RNA Expression with sequencing) Skin dataset, comprising approximately 25,000 cells. Prediction accuracy for the three smallest and three largest cell types is displayed, followed by the overall performance across all 22 cell types in the dataset (highlighted with a gray background).

(b) Five-time repeated random subsampling intra-dataset cell type prediction accuracy of Hydra against existing methods using the SNARE-seq (Single-Nucleus Chromatin Accessibility and mRNA Expression sequencing) Brain dataset, comprising approximately 8,000 cells. The overall prediction performance across all 23 cell types is indicated with a gray background.

(c) Comparison of five-time repeated random subsampling intra-dataset cell type prediction accuracy of Hydra against other methods using the sciCAR-seq (Single-Cell Combinatorial Indexing chromatin accessibility and RNA sequencing) Kidney dataset, comprising approximately 7,000 cells. Overall performance across all 14 cell types in the dataset is highlighted with a gray background.

(d) Five-time repeated random subsampling intra-dataset cell type prediction performance using the CITE-seq (Cellular Indexing of Transcriptomes and Epitopes by sequencing) PBMC dataset, comprising approximately 6,000 cells. The overall performance across all 26 cell types in the dataset is highlighted with a gray background.

(e) Visualization of the inter-dataset cell type prediction performance of various methods across four distinct single-cell multiome studies: 10X Multiome (10X Chromium Epi Multiome ATAC + Gene Expression), CITE-seq, and TEA-seq (Transcriptome, Epitope, and Accessibility sequencing), spanning 28 train-test splits. The bubble plot ranks methods based on their overall performance.

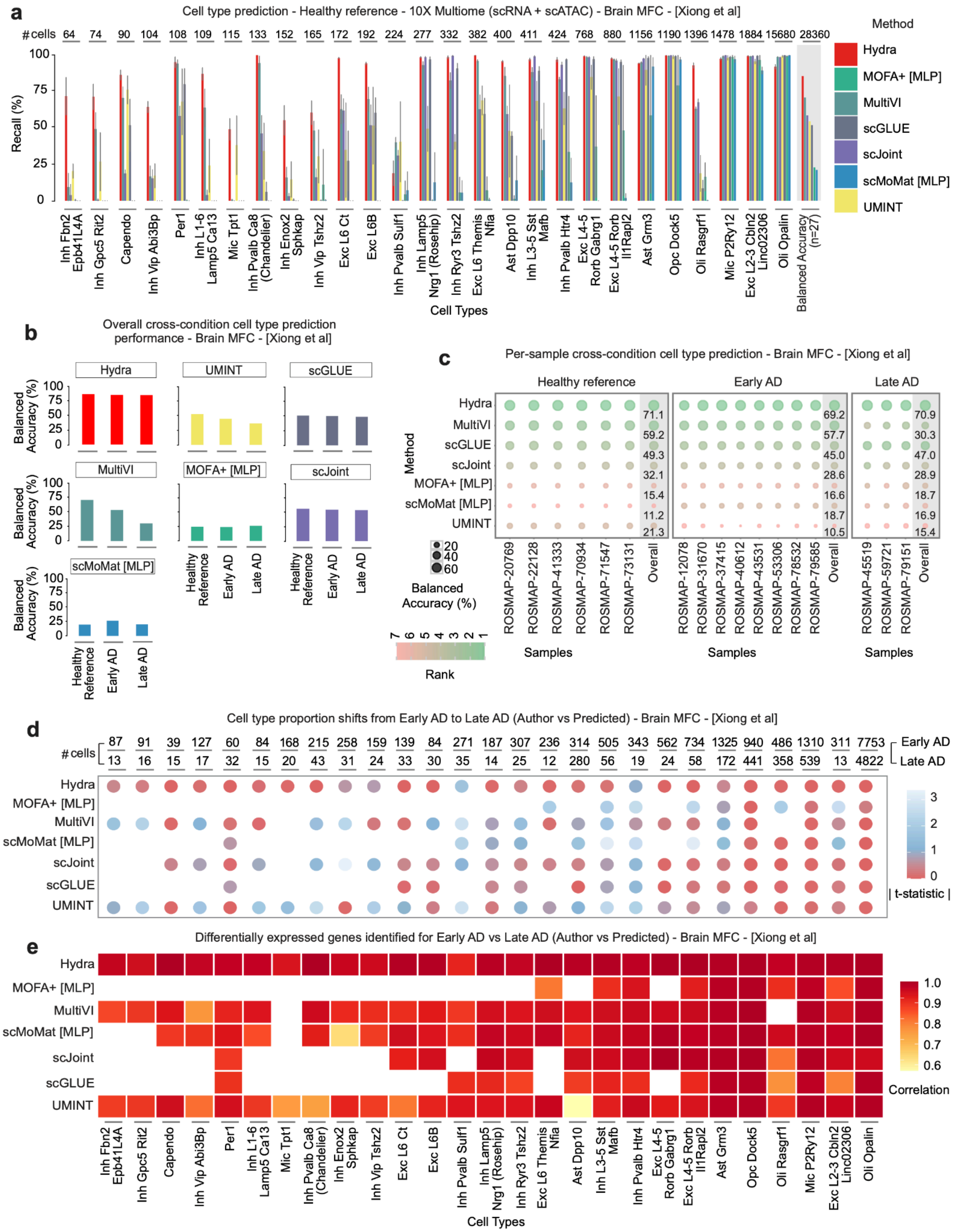

(a) Bar plot with error bars illustrating the 5-fold intra-dataset cell type prediction performance of Hydra against different single-cell multiome methods using healthy medial frontal cortex (MFC)

brain dataset. This dataset profiles RNA and ATAC modalities and comprises approximately 28,000 cells across 27 cell types. Prediction performance is displayed for all cell types, followed by the overall performance across all cell types in the dataset (highlighted with a gray background). Cell types are arranged in the order of increasing sample count from left to right.

(b) Faceted bar plots showing overall cell type prediction performance of different single-cell multiome methods across conditions trained on a healthy reference dataset, stratified by original cell type proportions. Error bars represent the variability in prediction accuracy across individual cell types.

(c) Bubble plot illustrating per-sample cross-condition identification of cell types using different single-cell multiome methods trained on healthy samples. The plot is faceted by conditions namely - healthy reference comprising held out healthy samples, early- and late-stage Alzheimer's disease held out sample. Methods are ranked based on overall performance across all conditions.

(d) Bubble plot showing absolute t-statistics quantifying cell type proportion changes from early to late stages of Alzheimer's disease. Each circle represents the magnitude of proportion shift differences between author label and predicted label for each cell type.

(e) Heatmap illustrating Spearman correlation coefficients between differential gene expression t-statistics derived from author annotations and method predictions for early versus late AD.

6. Inter-Dataset Comparison and Dataset Division

The inter-dataset comparison setup appears somewhat unclear. Based on Table 1, if training is performed on batch 1, each query dataset may still have partial overlap with the training data. Can the authors perform prediction on a completely unseen dataset to better evaluate cross-dataset generalization? If this is not the intended setup, please clarify the division scheme.

Response:

We thank the reviewer for this comment. Our inter-dataset comparison evaluates across both batches and studies. For single-cell transcriptomic datasets, we performed cross-study predictions across four tissues from different studies (Fig. 3). For single-cell multiome datasets, we have now additionally included a cross-study evaluation using CITE-seq datasets (Stephenson et al., 2021, Ramaswamy et al., 2021) (Fig. 4). Across both benchmarks, Hydra consistently demonstrated high balanced accuracy overall, outperforming other state-of-the-art methods. We have now clarified in the manuscript that inter-dataset evaluation refers to both batch- and study-level comparisons to avoid ambiguity.

7. Biological Meaningfulness

It would strengthen the work to provide clearer biological interpretations or examples demonstrating that the identified patterns or features are biologically meaningful.

Response:

We thank the reviewer for this suggestion. We have now performed gene ontology enrichment analysis on features selected by Hydra for three representative cell types including naive B cells, neutrophils, and mesenchymal cells, using the Tabula Muris dataset (Tabula Muris Consortium, et al., 2018). The enrichment results reveal functionally coherent processes specific to known biological roles of each cell type. We have now discussed this in the Results section of the manuscript. The added text reads as follows:

"*Finally, to evaluate whether features selected by Hydra capture biologically meaningful molecular processes, we performed gene ontology enrichment analysis for three representative cell types - naive B cells, neutrophils, and mesenchymal cells. Specifically, we used Enrichr (Kuleshov et al., 2016) on the top 100 features from each cell type and tested for enrichment (Appendix Fig. S4). For naive B cells, some of the relevant enriched terms included B cell activation, B cell receptor signaling pathway, and regulation of B cell proliferation, consistent with their adaptive immune functions. For neutrophils, enriched terms included neutrophil migration, neutrophil chemotaxis, inflammatory response and granulocyte chemotaxis, reflecting innate immune activation processes. Mesenchymal cells showed enrichment for extracellular matrix organization, collagen fibril organization, and skeletal system development, consistent with their structural role. These results demonstrate Hydra's ability to select functionally relevant and interpretable signatures that reflect core cell type-specific functions.*"

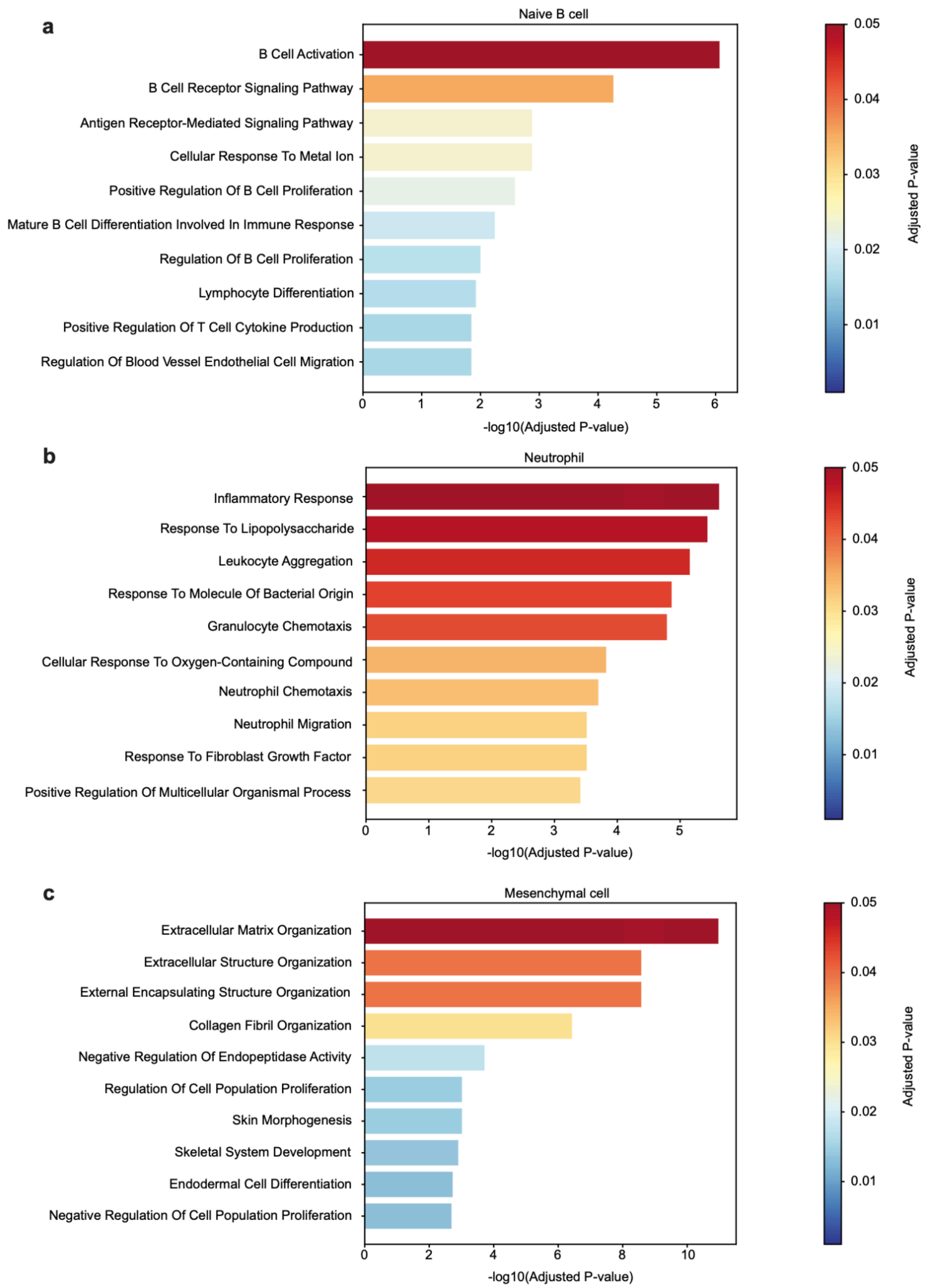
a
Naive B cell
B Cell Activation
B Cell Receptor Signaling Pathway
Antigen Receptor-Mediated Signaling Pathway
Cellular Response To Metal Ion
Positive Regulation Of B Cell Proliferation
Mature B Cell Differentiation Involved In Immune Response
Regulation Of B Cell Proliferation
Lymphocyte Differentiation
Positive Regulation Of T Cell Cytokine Production
Regulation Of Blood Vessel Endothelial Cell Migration
0 1 2 3 4 5 6
-log10(Adjusted P-value)
0.05 0.04 0.03 0.02 0.01
Adjusted P-value
b
Neutrophil
Inflammatory Response
Response To Lipopolysaccharide
Leukocyte Aggregation
Response To Molecule Of Bacterial Origin
Granulocyte Chemotaxis
Cellular Response To Oxygen-Containing Compound
Neutrophil Chemotaxis
Neutrophil Migration
Response To Fibroblast Growth Factor
Positive Regulation Of Multicellular Organismal Process
0 1 2 3 4 5
-log10(Adjusted P-value)
0.05 0.04 0.03 0.02 0.01
Adjusted P-value
c
Mesenchymal cell
Extracellular Matrix Organization
Extracellular Structure Organization
External Encapsulating Structure Organization
Collagen Fibril Organization
Negative Regulation Of Endopeptidase Activity
Regulation Of Cell Population Proliferation
Skin Morphogenesis
Skeletal System Development
Endodermal Cell Differentiation
Negative Regulation Of Cell Population Proliferation
0 2 4 6 8 10
-log10(Adjusted P-value)
0.05 0.04 0.03 0.02 0.01
Adjusted P-value

(a-c) Horizontal bar plots showing the top 10 significantly enriched terms (adjusted p-value < 0.05) for naive B cells (a), neutrophils (b), and mesenchymal cells (c). Enrichment analysis was performed using the top 100 features selected by Hydra for each cell type. Bar length represents -log10(adjusted p-value), and color intensity represents the significance level of enrichment.

8. Scalability
Please comment on the computational scalability of the proposed method. How does runtime or memory usage scale with increasing cell numbers or modalities compared with baseline methods?

Response:

We thank the reviewer for this valuable suggestion. To evaluate the computational scalability of Hydra, we measured both runtime and GPU memory consumption across varying dataset sizes for both single-cell transcriptomic (Madissoon et al., 2019) and single-cell multiome data (Ma et al., 2020, Stephenson et al., 2021). Our analysis shows that Hydra maintains consistent runtime performance, completing training in under 10 minutes across all dataset sizes and modalities (Fig. 6). Regarding GPU memory usage, Hydra exhibited higher peak memory consumption compared to baseline methods. Despite this, Hydra's computational cost remains within practical limits for its adoption in single-cell omics analysis. We have now included these results in the revised manuscript.

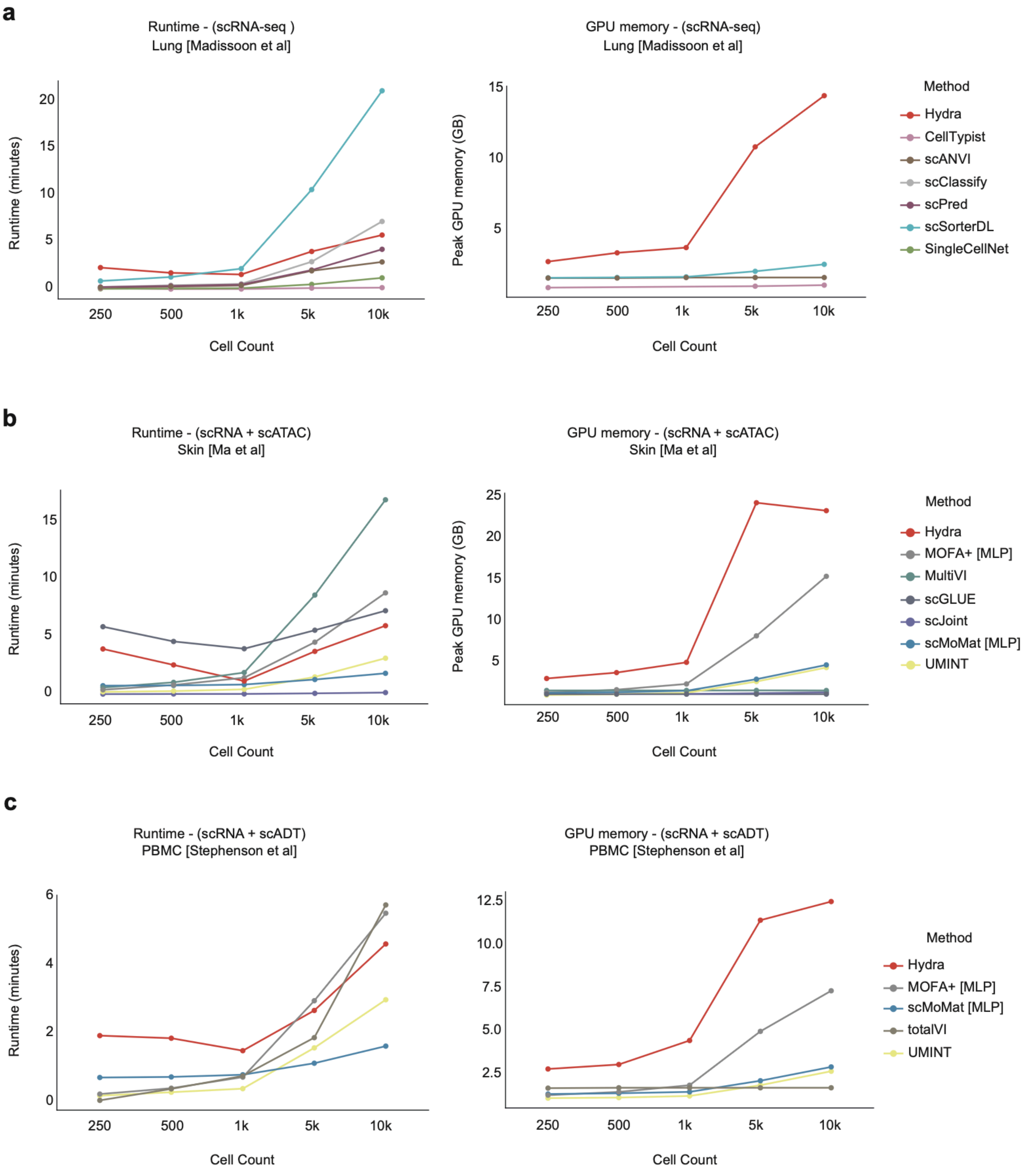

(a-c) Line plots showing runtime (left panel) and GPU consumption (right panel) of different methods across varying cell counts (250, 500, 1000, 5000, 10000). Evaluations were performed on single-cell transcriptomic data (a), joint profiling of single-cell transcriptomic and chromatin accessibility data (b) and joint profiling of single-cell transcriptomic and surface protein data (c)

9. Label Robustness

How robust is the method to label noise or annotation errors? Since many real-world datasets contain imperfect labels, an experiment introducing controlled label perturbations could be informative.

Response:

We thank the reviewer for their insightful comment regarding label noise. We acknowledge that cell type annotation errors are indeed prevalent in real-world single-cell omics datasets, often arising from subtle distinctions between biologically related cell types. Hydra is designed primarily as a supervised framework for feature selection and cell type annotation, rather than as a method for detecting or correcting label noise. While its ensemble design may partially mitigate the impact of minor annotation inconsistencies (Cao et al., 2020), we agree that a systematic evaluation using controlled label perturbations would be informative. We have updated the Discussion section of the manuscript to formally note this limitation and the necessity of incorporating sensitivity analyses in future work. The added paragraph reads as follows:

"*Although Hydra demonstrates robust performance across diverse datasets and biological conditions, we acknowledge limitations inherent to its supervised framework. First, the model is restricted to predicting cell types present in the training reference and does not currently support the discovery of novel or unannotated cell populations in query datasets. Second, as with most supervised tools, Hydra relies on the quality of input cell type labels in the reference dataset and significant label noise could potentially affect its prediction performance. Future extensions of the framework would therefore focus on integrating approaches to detect unseen cell populations and conduct systematic sensitivity analyses to address label noise.*"

10. Clarification on scVI vs. scANVI

Since scVI does not utilize labels, how are predictions generated for labeled tasks? Is the comparison actually with scANVI?

Response:

We thank the reviewer for raising this point. We acknowledge that scVI is an unsupervised method that does not utilize labels during training. In our analysis, predictions were generated by using scVI latent embeddings followed by Leiden clustering. The cluster identities were then assigned via majority voting against author labels. Since scANVI (Xu et al., 2021) is a semi-supervised extension that builds upon scVI, and leverages labeled data for supervised cell type prediction, we have now updated our evaluation to use scANVI. All results and figures in the manuscript have been updated to reflect this.

11. Single-Model Ablation Study and Downsampling

The section on single-model ablation could be clarified. Did the authors perform downsampling scheme? It may help to compare one model trained with downsampling and another without (original dataset) to avoid the ambiguity

Response:

We thank the reviewer for this comment. In our evaluation, all models were trained using the same cell type balancing approach, including the single-model version of Hydra. As suggested, we have now performed an additional comparison using the Lung dataset (Madissoon et al, 2019), in which we trained a single Hydra model with balancing (“HydraSingleModel”) and a single Hydra model without balancing (“Hydra_SingleModel_Original”; i.e., trained directly on the original dataset with imbalanced cell type proportions). We found that both single-model variants showed higher variability in feature stability and lower cell type prediction performance compared to the ensemble version of Hydra. These results demonstrate the respective contributions of class balancing and ensembling, demonstrating that while balancing provides modest gains, the ensemble design is the primary driver of Hydra’s robustness.

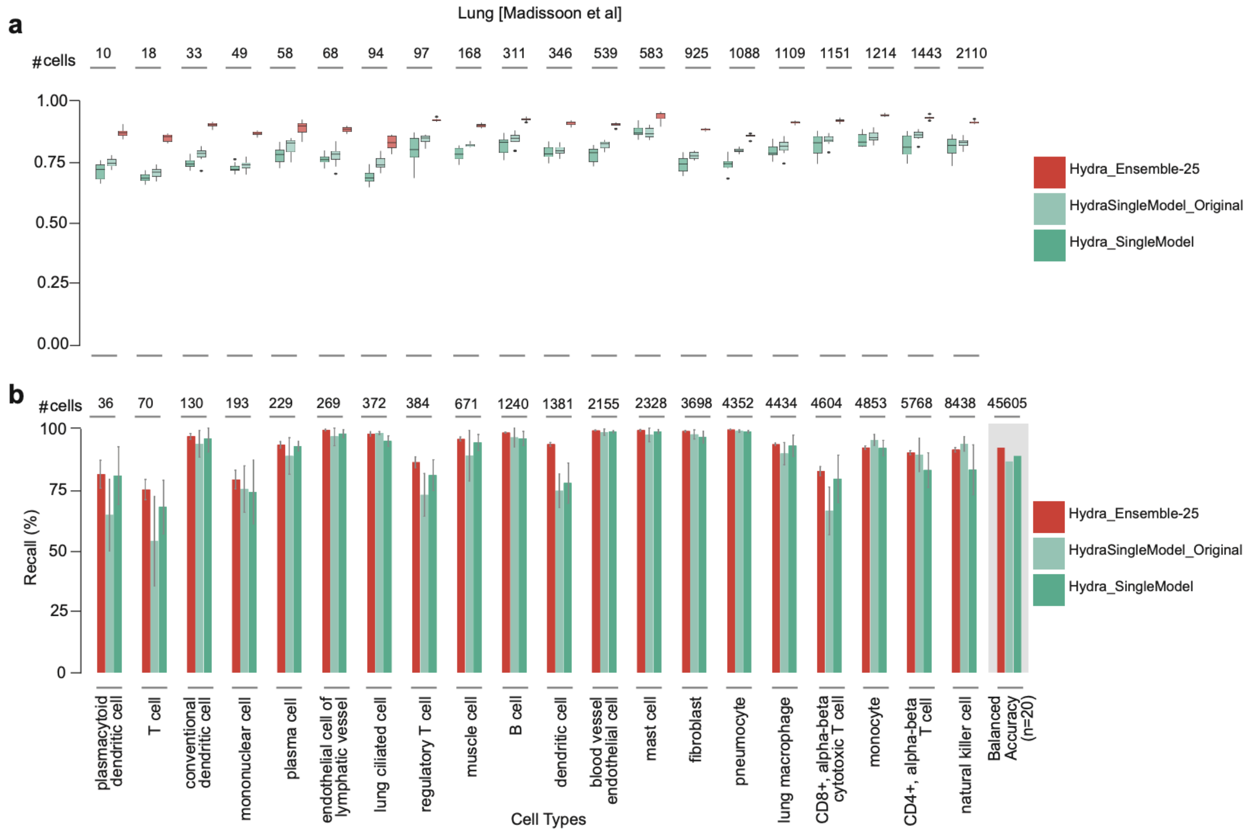

(a) Stability of features selected by Hydra model variants for all cell types in the lung dataset (n=20), quantified by Pearson correlation coefficients. Cell types are arranged in the order of increasing sample count from left to right.

(b) Cell type prediction performance of Hydra model variants for all cell types. Cell types are arranged in the order of increasing sample count from left to right, followed by the overall performance (highlighted with a gray background)

References:

- Ancona, Marco, et al. "Towards better understanding of gradient-based attribution methods for deep neural networks." arXiv preprint arXiv:1711.06104 (2017).
- Ashuach, Tal, et al. "MultiVI: deep generative model for the integration of multimodal data." Nature Methods 20.8 (2023): 1222-1231.
- Bai, Kailun, et al. "scSorterDL: a deep neural network-enhanced ensemble LDAs for single cell classifications." Briefings in Bioinformatics 26.5 (2025): bbaf446.
- Cao, Zhi-Jie, et al. "Multi-omics single-cell data integration and regulatory inference with graph-linked embedding." Nature Biotechnology 40.10 (2022): 1458-1466.
- Cao, Yue, et al. "Ensemble deep learning in bioinformatics." Nature Machine Intelligence 2.9 (2020): 500-508.
- Domínguez Conde, C., et al. "Cross-tissue immune cell analysis reveals tissue-specific features in humans." Science 376.6594 (2022): eabl5197.
- Gayoso, Adam, et al. "Joint probabilistic modeling of single-cell multi-omic data with totalVI." Nature methods 18.3 (2021): 272-282.
- Kuleshov, Maxim V., et al. "Enrichr: a comprehensive gene set enrichment analysis web server 2016 update." Nucleic acids research 44.W1 (2016): W90-W97.
- Hu, Luni, et al. "UniCell: Towards a Unified Solution for Cell Annotation, Nomenclature Harmonization, Atlas Construction in Single-Cell Transcriptomics." bioRxiv (2025): 2025-05.
- Lin, Yingxin, et al. "scJoint integrates atlas-scale single-cell RNA-seq and ATAC-seq data with transfer learning." Nature biotechnology 40.5 (2022): 703-710.
- Lipton, Zachary C. "The mythos of model interpretability: In machine learning, the concept of interpretability is both important and slippery." Queue 16.3 (2018): 31-57.
- Liu, Chunlei, et al. "Multi-task learning from multimodal single-cell omics with Matilda." Nucleic acids research 51.8 (2023): e45-e45.
- Liu, Tianyu, et al. "UNICORN: Towards universal cellular expression prediction with a multi-task learning framework." Nature Communications 16.1 (2025): 9455.
- Lundberg, Scott M., et al. "A unified approach to interpreting model predictions." Advances in neural information processing systems 30 (2017).
- Madissoon, E. et al. scrna-seq assessment of the human lung, spleen, and esophagus

tissue stability after cold preservation. Genome biology 21, 1 (2019).

- Ma, Sai, et al. "Chromatin potential identified by shared single-cell profiling of RNA and chromatin." Cell 183.4 (2020): 1103-1116.
- Murdoch, W. James, et al. "Definitions, methods, and applications in interpretable machine learning." Proceedings of the National Academy of Sciences 116.44 (2019): 22071-22080.
- Ramaswamy, Anjali, et al. "Immune dysregulation and autoreactivity correlate with disease severity in SARS-CoV-2-associated multisystem inflammatory syndrome in children." Immunity 54.5 (2021): 1083-1095.
- Simonyan, Karen, et al. "Deep inside convolutional networks: Visualising image classification models and saliency maps." arXiv preprint arXiv:1312.6034 (2013).
- Shrikumar, Avanti, et al. "Learning important features through propagating activation differences." International conference on machine learning. PMIR, 2017.
- Stephenson, Emily, et al. "Single-cell multi-omics analysis of the immune response in COVID-19." Nature medicine 27.5 (2021): 904-916.
- Sundararajan, Mukund, et al. "Axiomatic attribution for deep networks." International conference on machine learning. PMLR, (2017).
- Tabula Muris Consortium, et al. "Single-cell transcriptomics of 20 mouse organs creates a Tabula Muris." Nature 562.7727 (2018): 367-372.
- Wagle, Manoj M., et al. "Interpretable deep learning in single-cell omics." Bioinformatics 40.6 (2024): btae374.
- Xiong, Xushen, et al. "Epigenomic dissection of Alzheimer's disease pinpoints causal variants and reveals epigenome erosion." Cell 186.20 (2023): 4422-4437.
- Xu, Chenling, et al. "Probabilistic harmonization and annotation of single-cell transcriptomics data with deep generative models." Molecular systems biology 17.1 (2021): e9620.

6th Mar 2026

Manuscript Number: MSB-2025-13327R
Title: Interpretable deep generative ensemble learning for single-cell omics with Hydra

Dear Prof Yang,

Thank you for the submission of your revised manuscript to Molecular Systems Biology. We have now received the enclosed reports from the referees that were asked to re-assess it. As you will see the reviewers are now globally supportive and I am pleased to inform you that we will be able to accept your manuscript pending the following final amendments:

1) We require all corresponding authors to provide an ORCID ID in our manuscript submission system. Currently Ellis Patrick does not have an ORCID ID linked to their profile. A request with instructions was sent on 04.02.2026.
2) In the main manuscript file, please correct the first author's name, which is currently listed as Manoj M Author instead of Manoj M Wagle.
3) Email addresses of contributing authors is not needed on the manuscript title page, please remove.
4) Please combine the Code availability statement into the Data availability section and format according to the example below.
"The datasets and computer code produced in this study are available in the following databases:
- Chip-Seq data: Gene Expression Omnibus GSE46748 (https://www.ncbi.nlm.nih.gov/geo/query/acc.cgi?acc=GSE46748)
- Modeling computer scripts: GitHub (https://github.com/SysBioChalmers/GECKO/releases/tag/v1.0)
- [data type]: [full name of the resource] [accession number/identifier] ([doi or URL or identifiers.org/DATABASE:ACCESSION])"
5) Only newly generated datasets and code should be included in the Data Availability section. Information on publicly available datasets that have been analyzed should be provided in the Methods (the Appendix Table listing the datasets is fine). We also encourage authors to use Data citations (see below).
6) Please rename "Competing Interests" to "Disclosure and competing interests statement". We updated our journal's competing interests policy in January 2022 and request authors to consider both actual and perceived competing interests. Please review the policy https://link.springer.com/partners/embo-press/editorial-policies#Competing%20interest%20disclosures and update your competing interests if necessary.
7) Please correct the reference citation in the reference list to be alphabetical (not numerical). Where there are more than 10 authors on a paper, only the first 10 should be listed, followed by "et al.". Please check "Author Guidelines" for more information: https://link.springer.com/journal/44320/submission-guidelines#cms-Reference-guidelines
8) Our journal encourages inclusion of *data citations in the reference list* to directly cite datasets that were re-used and obtained from public databases. Data citations in the article text are distinct from normal bibliographical citations and should directly link to the database records from which the data can be accessed. In the main text, data citations are formatted as follows: "Data ref: Smith et al, 2001" or "Data ref: NCBI Sequence Read Archive PRJNA342805, 2017". In the Reference list, data citations must be labeled with "[DATASET]". A data reference must provide the database name, accession number/identifiers and a resolvable link to the landing page from which the data can be accessed at the end of the reference. Further instructions are available at .
9) All Materials and Methods need to be described in the main text using our 'Structured Methods' format. According to this format, the Methods section includes a Reagents and Tools Table (listing key reagents, experimental models, software and relevant equipment and including their sources and relevant identifiers) followed by a Methods and Protocols section describing the methods, ideally using a step-by-step protocol format. The aim is to facilitate adoption of the methodologies across labs. Please download and fill our Reagents and Tools Table template (.docx), which you can find in our author guidelines: https://www.embopress.org/page/journal/14693178/authorguide#structuredmethods.
When submitting your revised manuscript, please do not include the Reagents and Tools Table in the Methods section of the manuscript but upload it as a separate file choosing the file type "Reagent Table".
10) Please place individual sections of the manuscript in the following order: Title page - Abstract & Keywords - Introduction - Results - Discussion - Methods - Data Availability - Acknowledgements - Disclosure and Competing Interests Statement - References - Figure Legends - Expanded View Figure Legends.
11) For the figures and figure legends, please take care of the following:
- Please move the legend for Figure 1 to the end of the manuscript, together with all of the other legends.
- Please make sure to update the callouts of all figures in the main manuscript text. Currently figure callouts are missing for Figures 2B and 6A-C.
- Please place the heading "Figure Legends" before listing the figure legends
- Please rectify the labels in Appendix Figure S7 caption (a-d instead of a-b)
- Please note that the box plots need to be defined in terms of minima, maxima, centre, bounds of box and whiskers, and percentile in the legends of figures 2A, B
- Please note that the error bars are not defined in the legends of figures 3B, C; 4A-D; 5A
12) Please remove the following section from the manuscript: 'Supplementary information. All supplementary information are provided in the appendix.'
13) In the Appendix file, please ensure that the title page has the title "Appendix for + manuscript title".
14) Please ensure that all funding sources that are entered into the manuscript submission system are also listed in the

manuscript. Currently grant 1173472 is not listed in the manuscript.
15) Synopsis:
- Synopsis image: Please provide a graphic that summarises the main findings of the manuscript on a glance and upload it as a high-resolution jpeg file 550 pixels wide x (300-600) pixels high.
- Synopsis text: Please provide a separate word document including a short standfirst (maximum of 300 characters, including spaces) and up to 5 bullet points to summarise the key NEW findings. They should be designed to be complementary to the abstract - i.e. not repeat the same text. We encourage inclusion of key acronyms and quantitative information (maximum of 30 words / bullet point). Please use the passive voice.
- Please check your synopsis text and image before submission with your revised manuscript. Please be aware that in the proof stage minor corrections only are allowed (e.g., typos).
16) Source Data: Source Data should be organized as a single source data file (zipped) per figure for main figures (all EV and/or Appendix figure Source Data can be included in a single folder), with the panels clearly visible in the folder structure instead of a single excel file for all Source Data. e.g. all the Source data files for figure 1 need to be saved in a single folder and this needs to be zipped and then uploaded as "SD figure 1.zip" file, rather than all Source Data zipped together in 1 folder. Please also update the Source Data checklist to indicate that Source Data for Figure 2D has been provided.
17) As part of the EMBO Publications transparent editorial process initiative (see our policy here: https://www.embopress.org/transparent-process#Review_Process), Molecular Systems Biology will publish online a Peer Review File (PRF) to accompany accepted manuscripts. This file will be published in conjunction with your paper and will include the anonymous referee reports, your point-by-point response and all pertinent correspondence relating to the manuscript. Let us know whether you agree with the publication of the PRF and as here, if you want to remove or not any figures from it prior to publication. Please note that the Authors checklist will be published at the end of the PRF.
18) After your paper is published, we may promote it on social media. If you have any handles or hashtags for Bluesky you would like included, please let us know.
19) Please provide a point-by-point letter INCLUDING my comments and your detailed responses (as Word file).

I look forward to reading a new revised version of your manuscript as soon as possible.

Yours sincerely,

Poonam Bheda, PhD
Scientific Editor
Molecular Systems Biology

Reviewer #1:

The authors have satisfactorily addressed the major concerns that I raised in the previous round. The revisions substantially improve methodological clarity, benchmarking rigor, interpretability framing, and transparency. I have no further major concerns.

Reviewer #2:

The authors have responded to my questions clearly, and I have no further inquiries.

Reviewer #3:

The authors have addressed all my comments.

Response Letter

1) We require all corresponding authors to provide an ORCID ID in our manuscript submission system. Currently Ellis Patrick does not have an ORCID ID linked to their profile. A request with instructions was sent on 04.02.2026.

Response: We have now linked the ORCID ID for Ellis Patrick in the manuscript submission system.

2) In the main manuscript file, please correct the first author's name, which is currently listed as Manoj M Author instead of Manoj M Wagle.

Response: We have corrected the first author's name to "Manoj M Wagle" in the main manuscript file.

3) Email addresses of contributing authors is not needed on the manuscript title page, please remove.

Response: We have removed the email addresses of the contributing authors from the manuscript title page.

4) Please combine the Code availability statement into the Data availability section and format according to the example below.

"The datasets and computer code produced in this study are available in the following databases:

- Chip-Seq data: Gene Expression Omnibus GSE46748 (https://www.ncbi.nlm.nih.gov/geo/query/acc.cgi?acc=GSE46748)

- Modeling computer scripts: GitHub (https://github.com/SysBioChalmers/GECKO/releases/tag/v1.0)

- [data type]: [full name of the resource] [accession number/identifier] ([doi or URL or identifiers.org/DATABASE:ACCESSION])"

Response: We have incorporated the Code availability statement into the Data availability section, following the above recommended format.

5) Only newly generated datasets and code should be included in the Data Availability section. Information on publicly available datasets that have been analyzed should be provided in the Methods (the Appendix Table listing the datasets is fine). We also encourage authors to use Data citations (see below).

Response: The data statement for publicly available datasets has been removed from the Data availability section. All datasets analyzed in this study are listed in the Appendix table and referenced in the Methods section.

6) Please rename "Competing Interests" to "Disclosure and competing interests statement". We

updated our journal's competing interests policy in January 2022 and request authors to consider both actual and perceived competing interests. Please review the policy https://link.springer.com/partners/embo-press/editorial-policies#Competing%20interest%20disclosures and update your competing interests if necessary.

Response: We have renamed the "Competing Interests" section to "Disclosure and Competing Interests Statement" in the manuscript.

7) Please correct the reference citation in the reference list to be alphabetical (not numerical). Where there are more than 10 authors on a paper, only the first 10 should be listed, followed by "et al.". Please check "Author Guidelines" for more information: https://link.springer.com/journal/44320/submission-guidelines#cms-Reference-guidelines

Response: We have corrected the reference list to follow alphabetical ordering. For papers with more than 10 authors, we have listed only the first 10 authors followed by "et al."

8) Our journal encourages inclusion of *data citations in the reference list* to directly cite datasets that were re-used and obtained from public databases. Data citations in the article text are distinct from normal bibliographical citations and should directly link to the database records from which the data can be accessed. In the main text, data citations are formatted as follows: "Data ref: Smith et al, 2001" or "Data ref: NCBI Sequence Read Archive PRJNA342805, 2017". In the Reference list, data citations must be labeled with "[DATASET]". A data reference must provide the database name, accession number/identifiers and a resolvable link to the landing page from which the data can be accessed at the end of the reference. Further instructions are available at <https://www.embopress.org/page/journal/17574684/authorguide#referencesformat>.

Response: We have included all datasets utilized in the study in the Appendix table and cited these in the manuscript.

9) All Materials and Methods need to be described in the main text using our 'Structured Methods' format. According to this format, the Methods section includes a Reagents and Tools Table (listing key reagents, experimental models, software and relevant equipment and including their sources and relevant identifiers) followed by a Methods and Protocols section describing the methods, ideally using a step-by-step protocol format. The aim is to facilitate adoption of the methodologies across labs.

Please download and fill our Reagents and Tools Table template (.docx), which you can find in our author guidelines: https://www.embopress.org/page/journal/14693178/authorguide#structuredmethods.

When submitting your revised manuscript, please do not include the Reagents and Tools Table in the Methods section of the manuscript but upload it as a separate file choosing the file type "Reagent Table".

Response: We have filled in and uploaded the Reagents Table as a separate file.

10) Please place individual sections of the manuscript in the following order: Title page -

Abstract & Keywords - Introduction - Results - Discussion - Methods - Data Availability - Acknowledgements - Disclosure and Competing Interests Statement - References - Figure Legends - Expanded View Figure Legends.

Response: We have reordered the individual sections of the manuscript following the recommended order.

11) For the figures and figure legends, please take care of the following:

- Please move the legend for Figure 1 to the end of the manuscript, together with all of the other legends.

Response: We have moved the legends for all figures to the end of the manuscript.

- Please make sure to update the callouts of all figures in the main manuscript text. Currently figure callouts are missing for Figures 2B and 6A-C.

Response: We have updated the missing callouts for Figures 2B and 6A-C in the main text.

- Please place the heading "Figure Legends" before listing the figure legends

Response: We have placed the heading "Figure Legends" before listing the figure legends.

- Please rectify the labels in Appendix Figure S7 caption (a-d instead of a-b)

Response: We have corrected the Appendix Figure S7 caption.

- Please note that the box plots need to be defined in terms of minima, maxima, centre, bounds of box and whiskers, and percentile in the legends of figures 2A, B

Response: We have defined the box plots in the legends of Figures 2A and 2B.

- Please note that the error bars are not defined in the legends of figures 3B, C; 4A-D; 5A

Response: We have defined the error bars in the legends of Figures 3B-C, 4A-D, and 5A.

12) Please remove the following section from the manuscript: 'Supplementary information. All supplementary information are provided in the appendix.'

Response: We have removed the section "Supplementary information" from the manuscript.

13) In the Appendix file, please ensure that the title page has the title "Appendix for + manuscript title".

Response: We have updated the Appendix title page to read: "Appendix for Interpretable Deep

Generative Ensemble Learning for Single-Cell Omics with Hydra".

14) Please ensure that all funding sources that are entered into the manuscript submission system are also listed in the manuscript. Currently grant 1173472 is not listed in the manuscript.

Response: We note that the grant number was incorrectly entered in the submission system. We have added the following statement to the Acknowledgments section: "This work is supported by scholarships from The University of Sydney and Children's Medical Research Institute to M.M.W, The University of Sydney Robinson Fellowship and National Health and Medical Research Council (NHMRC) Investigator Grant (1173469) to P.Y.."

15) Synopsis:

- Synopsis image: Please provide a graphic that summarises the main findings of the manuscript on a glance and upload it as a high-resolution jpeg file 550 pixels wide x (300-600) pixels high.

- Synopsis text: Please provide a separate word document including a short standfirst (maximum of 300 characters, including spaces) and up to 5 bullet points to summarise the key NEW findings. They should be designed to be complementary to the abstract - i.e. not repeat the same text. We encourage inclusion of key acronyms and quantitative information (maximum of 30 words / bullet point). Please use the passive voice.

- Please check your synopsis text and image before submission with your revised manuscript. Please be aware that in the proof stage minor corrections only are allowed (e.g., typos).

Response: We have uploaded a synopsis image summarising the main findings of the manuscript. We have also uploaded the synopsis text, including the standfirst and bullet points summarising the key findings of the study.

16) Source Data: Source Data should be organized as a single source data file (zipped) per figure for main figures (all EV and/or Appendix figure Source Data can be included in a single folder), with the panels clearly visible in the folder structure instead of a single excel file for all Source Data. e.g. all the Source data files for figure 1 need to be saved in a single folder and this needs to be zipped and then uploaded as "SD figure 1.zip" file, rather than all Source Data zipped together in 1 folder. Please also update the Source Data checklist to indicate that Source Data for Figure 2D has been provided.

Response: We have ensured that the source data for each main figure is organised into individual folders and uploaded as separate zipped files. We have also updated the Source Data checklist to indicate that Source Data for Figure 2D has been provided.

17) As part of the EMBO Publications transparent editorial process initiative (see our policy here: https://www.embopress.org/transparent-process#Review_Process), Molecular Systems Biology will publish online a Peer Review File (PRF) to accompany accepted manuscripts. This file will be published in conjunction with your paper and will include the anonymous referee reports, your point-by-point response and all pertinent correspondence relating to the manuscript. Let us know whether you agree with the publication of the PRF and as here, if you want to remove or not any figures from it prior to publication. Please note that the Authors checklist will be published at the end of the PRF.

Response: We agree to the publication of the Peer Review File.

18) After your paper is published, we may promote it on social media. If you have any handles or hashtags for Bluesky you would like included, please let us know.

Response: We have a X handle @PengyiYang82. We do not have any Bluesky handles to include.

19) Please provide a point-by-point letter INCLUDING my comments and your detailed responses (as Word file).

Response: We have attached this point-by-point response letter.

24th Mar 2026
Manuscript number: MSB-2025-13327RR
Title: Interpretable deep generative ensemble learning for single-cell omics with Hydra

Dear Prof Yang,

Thank you again for sending us your revised manuscript. We are now satisfied with the modifications made and I am pleased to inform you that your paper has been accepted for publication.

Your manuscript will be processed for publication by EMBO Press. It will be copy edited and you will receive page proofs prior to publication. Please note that you will be contacted by Springer Nature Author Services to complete licensing and payment information.

You may qualify for financial assistance for your publication charges - either via a Springer Nature fully open access agreement or an EMBO initiative. Check your eligibility: https://link.springer.com/journal/44320/how-to-publish-with-us

Should you be planning a Press Release on your article, please get in contact with embo_production@springernature.com as early as possible in order to coordinate publication and release dates.

If you have any questions, please do not hesitate to contact the Editorial Office. Thank you for your contribution to Molecular Systems Biology.

Yours sincerely,

Poonam Bheda, PhD
Scientific Editor
Molecular Systems Biology

>>> Please note that it is Molecular Systems Biology policy for the transcript of the editorial process (containing referee reports and your response letter) to be published as an online supplement to each paper. If you do NOT want this, you will need to inform the Editorial Office via email immediately. More information is available here: https://link.springer.com/partners/embo-press/editorial-policies#Peer%20review

EMBO Press Author Checklist

Corresponding Author Name: Ellis Patrick; Pengyi Yang
Journal Submitted to: Molecular Systems Biology
Manuscript Number: MSB-2025-13327R

USEFUL LINKS FOR COMPLETING THIS FORM
The EMBO Journal - Author Guidelines
EMBO Reports - Author Guidelines
Molecular Systems Biology - Author Guidelines
EMBO Molecular Medicine - Author Guidelines

Reporting Checklist for Life Science Articles (updated January
This checklist is adapted from Materials Design Analysis Reporting (MDAR) Checklist for Authors. MDAR establishes a minimum set of requirements in transparent reporting in the life sciences (see Statement of Task: 10.31222/osf.io/9sm4x). Please follow the journal's guidelines in preparing your manuscript.
Please note that a copy of this checklist will be published alongside your article.

Abridged guidelines for figures
1. Data
The data shown in figures should satisfy the following conditions:
- the data were obtained and processed according to the field's best practice and are presented to reflect the results of the experiments in an accurate and unbiased manner.
- ideally, figure panels should include only measurements that are directly comparable to each other and obtained with the same assay.
- plots include clearly labeled error bars for independent experiments and sample sizes. Unless justified, error bars should not be shown for technical
- if n<5, the individual data points from each experiment should be plotted. Any statistical test employed should be justified.
- Source Data should be included to report the data underlying figures according to the guidelines set out in the authorship guidelines on Data

2. Captions
Each figure caption should contain the following information, for each panel where they are relevant:
- a specification of the experimental system investigated (eg cell line, species name).
- the assay(s) and method(s) used to carry out the reported observations and measurements.
- an explicit mention of the biological and chemical entity(ies) that are being measured.
- an explicit mention of the biological and chemical entity(ies) that are altered/varied/perturbed in a controlled manner.
- the exact sample size (n) for each experimental group/condition, given as a number, not a range;
- a description of the sample collection allowing the reader to understand whether the samples represent technical or biological replicates (including how many animals, litters, cultures, etc.).
- a statement of how many times the experiment shown was independently replicated in the laboratory.
- definitions of statistical methods and measures:
  - common tests, such as t-test (please specify whether paired vs. unpaired), simple χ2 tests, Wilcoxon and Mann-Whitney tests, can be unambiguously identified by name only, but more complex techniques should be described in the methods section;
  - are tests one-sided or two-sided?
  - are there adjustments for multiple comparisons?
  - exact statistical test results, e.g., P values = x but not P values < x;
  - definition of 'center values' as median or average;
  - definition of error bars as s.d. or s.e.m.

Please complete ALL of the questions below.
Select "Not Applicable" only when the requested information is not relevant for your study.

Materials

Newly Created Materials	Information included in the manuscript?	In which section is the information available? (Reagents and Tools Table, Materials and Methods, Figures, Data Availability Section)
New materials and reagents need to be available; do any restrictions apply?	Not Applicable	

Antibodies	Information included in the manuscript?	In which section is the information available? (Reagents and Tools Table, Materials and Methods, Figures, Data Availability Section)
For **antibodies** provide the following information:<br>- Commercial antibodies: RRID (if possible) or supplier name, catalogue number and or/clone number<br>- Non-commercial: RRID or citation	Not Applicable	

DNA and RNA sequences	Information included in the manuscript?	In which section is the information available? (Reagents and Tools Table, Materials and Methods, Figures, Data Availability Section)
**Short novel DNA or RNA including primers, probes**: provide the sequences.	Not Applicable	

Cell materials	Information included in the manuscript?	In which section is the information available? (Reagents and Tools Table, Materials and Methods, Figures, Data Availability Section)
**Cell lines:** Provide species information, strain. Provide accession number in repository **OR** supplier name, catalog number, clone number, and/**OR** RRID.	Not Applicable	
**Primary cultures:** Provide species, strain, sex of origin, genetic modification status.	Not Applicable	
Report if the cell lines were recently **authenticated** (e.g., by STR profiling) and tested for mycoplasma contamination.	Not Applicable	

Experimental animals	Information included in the manuscript?	In which section is the information available? (Reagents and Tools Table, Materials and Methods, Figures, Data Availability Section)
**Laboratory animals or Model organisms:** Provide species, strain, sex, age, genetic modification status. Provide accession number in repository **OR** supplier name, catalog number, clone number, **OR** RRID.	Not Applicable	
**Animal observed in or captured from the field:** Provide species, sex, and age where possible**.**	Not Applicable	
Please detail **housing and husbandry conditions**.	Not Applicable	

Plants and microbes	Information included in the manuscript?	In which section is the information available? (Reagents and Tools Table, Materials and Methods, Figures, Data Availability Section)
**Plants:** provide species and strain, ecotype and cultivar where relevant, unique accession number if available, and source (including location for collected wild specimens).	Not Applicable	
**Microbes:** provide species and strain, unique accession number if available, and source.	Not Applicable	

Human research participants	Information included in the manuscript?	In which section is the information available? (Reagents and Tools Table, Materials and Methods, Figures, Data Availability Section)
If collected and within the bounds of privacy constraints report on age, sex and gender or ethnicity for all study participants.	Not Applicable	

Core facilities	Information included in the manuscript?	In which section is the information available? (Reagents and Tools Table, Materials and Methods, Figures, Data Availability Section)
If your work benefited from core facilities, was their service mentioned in the acknowledgments section?	Yes	Acknowledgments

Design

Study protocol	Information included in the manuscript?	In which section is the information available? (Reagents and Tools Table, Materials and Methods, Figures, Data Availability Section)
If study protocol has been **pre-registered, provide DOI in the manuscript**. For clinical trials, provide the trial registration number **OR** cite DOI.	Not Applicable	
Report the **clinical trial registration number** (at ClinicalTrials.gov or equivalent), where applicable.	Not Applicable	

Laboratory protocol	Information included in the manuscript?	In which section is the information available? (Reagents and Tools Table, Materials and Methods, Figures, Data Availability Section)
Provide DOI OR other citation details if **external detailed step-by-step protocols** are available.	Not Applicable	

Experimental study design and statistics	Information included in the manuscript?	In which section is the information available? (Reagents and Tools Table, Materials and Methods, Figures, Data Availability Section)
Include a statement about **sample size** estimate even if no statistical methods were used.	Not Applicable	
Were any steps taken to minimize the effects of subjective bias when allocating animals/samples to treatment (e.g. **randomization procedure**)? If yes, have they been described?	Not Applicable	
Include a statement about **blinding** even if no blinding was done.	Not Applicable	
Describe **inclusion/exclusion criteria** if samples or animals were excluded from the analysis. Were the criteria pre-established?<br><br>If sample or data points were omitted from analysis, report if this was due to attrition or intentional exclusion and provide justification.	Not Applicable	
For every figure, are **statistical tests** justified as appropriate? Do the data meet the assumptions of the tests (e.g., normal distribution)? Describe any methods used to assess it. Is there an estimate of variation within each group of data? Is the variance similar between the groups that are being statistically compared?	Not Applicable	

Sample definition and in-laboratory replication	Information included in the manuscript?	In which section is the information available? (Reagents and Tools Table, Materials and Methods, Figures, Data Availability Section)
In the figure legends: state number of times the experiment was **replicated** in laboratory.	Not Applicable	
In the figure legends: define whether data describe **technical or biological replicates**.	Not Applicable	

Ethics

Ethics	Information included in the manuscript?	In which section is the information available? (Reagents and Tools Table, Materials and Methods, Figures, Data Availability Section)
Studies involving **human participants**: State details of **authority granting ethics approval** (IRB or equivalent committee(s), provide reference number for approval.	Not Applicable	
Studies involving **human participants**: Include a statement confirming that **informed consent** was obtained from all subjects and that the experiments conformed to the principles set out in the WMA Declaration of Helsinki and the Department of Health and Human Services Belmont Report.	Not Applicable	
Studies involving **human participants:** For publication of **patient photos**, include a statement confirming that consent to publish was obtained.	Not Applicable	
Studies involving experimental **animals**: State details of **authority granting ethics approva**l (IRB or equivalent committee(s), provide reference number for approval. Include a statement of compliance with ethical regulations.	Not Applicable	
Studies involving **specimen and field samples:** State if relevant **permits** obtained, provide details of authority approving study; if none were required, explain why.	Not Applicable	

Dual Use Research of Concern (DURC)	Information included in the manuscript?	In which section is the information available? (Reagents and Tools Table, Materials and Methods, Figures, Data Availability Section)
Could your study fall under dual use research restrictions? Please check biosecurity documents and list of **select agents and toxins** (CDC): https://www.selectagents.gov/sat/list.htm	Not Applicable	
If you used a select agent, is the security level of the lab appropriate and reported in the manuscript?	Not Applicable	
If a study is subject to dual use research of concern regulations, is the name of the **authority granting approval and reference number** for the regulatory approval provided in the manuscript?	Not Applicable	

Reporting

The MDAR framework recommends adoption of discipline-specific guidelines, established and endorsed through community initiatives. Journals have their own policy about requiring specific guidelines and recommendations to complement MDAR.

Adherence to community standards	Information included in the manuscript?	In which section is the information available? (Reagents and Tools Table, Materials and Methods, Figures, Data Availability Section)
State if relevant guidelines or checklists (e.g., **ICMJE, MIBBI, ARRIVE, PRISMA**) have been followed or provided.	Not Applicable	
For **tumor marker prognostic studies,** we recommend that you follow the **REMARK** reporting guidelines (see link list at top right). See author guidelines, under 'Reporting Guidelines'. Please confirm you have followed these guidelines.	Not Applicable	
For **phase II and III randomized controlled trials**, please refer to the **CONSORT** flow diagram (see link list at top right) and submit the CONSORT checklist (see link list at top right) with your submission. See author guidelines, under 'Reporting Guidelines'. Please confirm you have submitted this list.	Not Applicable	

Data Availability

Data availability	Information included in the manuscript?	In which section is the information available? (Reagents and Tools Table, Materials and Methods, Figures, Data Availability Section)
Have **primary datasets** been deposited according to the journal's guidelines (see 'Data Deposition' section) and the respective accession numbers provided in the Data Availability Section?	Not Applicable	
Were **human clinical and genomic datasets** deposited in a public access-controlled repository in accordance to ethical obligations to the patients and to the applicable consent agreement?	Not Applicable	
Are **computational models** that are central and integral to a study available without restrictions in a machine-readable form? Were the relevant accession numbers or links provided?	Yes	Data availability
If publicly available data were reused, provide the respective **data citations in the reference list**.	Yes	References
